# An evolutionary analysis of cAMP-specific Phosphodiesterase 4 alternative splicing

**DOI:** 10.1186/1471-2148-10-247

**Published:** 2010-08-11

**Authors:** Keven R Johnson, Jessie Nicodemus-Johnson, Robert S Danziger

**Affiliations:** 1 Department of Physiology and Biophysics, University of Illinois at Chicago 835 S. Wolcott Avenue, M/C 901, Chicago, IL 60612-7342 USA; 2 Department of Human Genetics, University of Chicago 5812 S. Ellis Avenue Chicago, IL 60637 USA; 3 Department of Research and Development, Jesse Brown VA Medical Center 820 S. Damen Avenue, Chicago IL 60612 USA; 4 Department of Medicine, Division of Cardiology, University of Illinois at Chicago 840 S. Wood Street, M/C 715 Chicago IL 60612 USA

## Abstract

**Background:**

Cyclic nucleotide phosphodiesterases (PDEs) hydrolyze the intracellular second messengers: cyclic adenosine monophosphate (cAMP) and cyclic guanine monophosphate (cGMP). The cAMP-specific PDE family 4 (PDE4) is widely expressed in vertebrates. Each of the four PDE4 gene isoforms (PDE4 A-D) undergo extensive alternative splicing via alternative transcription initiation sites, producing unique amino termini and yielding multiple splice variant forms from each gene isoform termed long, short, super-short and truncated super-short. Many species across the vertebrate lineage contain multiple splice variants of each gene type, which are characterized by length and amino termini.

**Results:**

A phylogenetic approach was used to visualize splice variant form genesis and identify conserved splice variants (genome conservation with EST support) across the vertebrate taxa. Bayesian and maximum likelihood phylogenetic inference indicated PDE4 gene duplication occurred at the base of the vertebrate lineage and reveals additional gene duplications specific to the teleost lineage. Phylogenetic inference and PDE4 splice variant presence, or absence as determined by EST screens, were further supported by the genomic analysis of select vertebrate taxa. Two conserved PDE4 long form splice variants were found in each of the PDE4A, PDE4B, and PDE4C genes, and eight conserved long forms from the PDE4 D gene. Conserved short and super-short splice variants were found from each of the PDE4A, PDE4B, and PDE4 D genes, while truncated super-short variants were found from the PDE4C and PDE4 D genes. PDE4 long form splice variants were found in all taxa sampled (invertebrate through mammals); short, super-short, and truncated super-short are detected primarily in tetrapods and mammals, indicating an increasing complexity in both alternative splicing and cAMP metabolism through vertebrate evolution.

**Conclusions:**

There was a progressive independent incorporation of multiple PDE4 splice variant forms and amino termini, increasing PDE4 proteome complexity from primitive vertebrates to humans. While PDE4 gene isoform duplicates with limited alternative splicing were found in teleosts, an expansion of both PDE4 splice variant forms, and alternatively spliced amino termini predominantly occurs in mammals. Since amino termini have been linked to intracellular targeting of the PDE4 enzymes, the conservation of amino termini in PDE4 splice variants in evolution highlights the importance of compartmentalization of PDE4-mediated cAMP hydrolysis.

## Background

Cyclic nucleotide phosphodiesterases (PDEs) catalyze the hydrolysis of cyclic nucleotide second messengers; cyclic adenosine monophosphate (cAMP) and cyclic guanosine monophosphate (cGMP) [1,2]. The PDE4 family is one of three cAMP-specific PDE families. PDE4 s have been shown to regulate several cellular physiological processes such as; protein phosphorylation via cAMP-dependent protein kinase A (PKA), gene transcription through cAMP response elements, and cyclic nucleotide gated ion channels [3-6]. These processes have been linked to cognitive function, depression, schitzophrenia, hypertension, and an integral involvement in modulating cardiomyocyte contractility [7,8].

**Figure 1:**
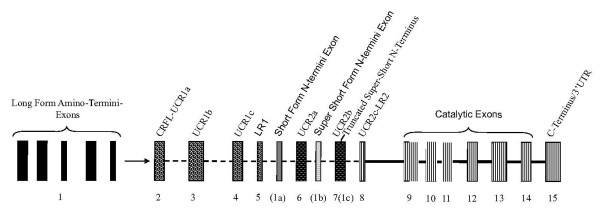
**Overview of representative PDE4 gene structure**. PDE4 genes are composed of multiple exons connected by either a dashed line (facultative exons), or solid line (constitutive exons). The PDE4 long form amino-termini specifying exons (1), are located in the upstream region of the genes, each under different promoter control. The Upstream conserved region-1 (UCR1) is composed of three exons (UCR1a, UCR1b, and UCR1c). The PDE4 short form amino termini specifying exon(s) (1a) are located downstream of the linker region 1 (LR1) exon. Upstream conserved region 2 (UCR2) is composed of three exons (UCR2a, UCR2b and UCR2c), which are interrupted by the super-short form amino-terminus specifying exon (1b). The amino-terminus of truncated super-short PDE4 splice variants is found within the UCR2b exon (1c). The enzymatic core of PDE4 is encoded by several constitutive exons (found in all isoforms) located in the farthest downstream regions of the gene.

The PDE4 gene family is composed of four gene isoforms; *PDE4A*, *PDE4B*, *PDE4C*, and *PDE4 D *which arose via a gene duplication event in a common eukaryotic ancestor before the separation of sponges and eumetazoans [9]. Although PDE4 s have not been extensively studied in teleosts, amphibians, or reptiles, transcripts from all four PDE4 gene isoforms have been detected in several mammalian species; for review, [8]. Mammalian PDE4 splice variant forms produced from each gene isoform (A-D) have been classified as: long, short, super-short, or truncated super-short [7,10] (Figure 1). PDE4 splice variant synthesis proceeds through alternative splicing via the incorporation of distinct promoters (alternative transcription start sites) that drive differential tissue expression and transcriptional regulation [11-13]. The long form transcripts consist of exons encoding the amino termini (i.e., 5' exon), upstream conserved region-1 (UCR-1), linker region-1 (LR-1), UCR-2, LR2, catalytic, and carboxy-terminal domains (Figure 1). Each PDE4 gene isoforms produces multiple PDE4 long forms with unique amino termini. The PDE4 amino terminal protein region is proposed to be responsible for variant-specific protein-protein interactions providing subcellular localizations for PDE4 splice variants [14-16]. The UCR-1 and UCR-2 facilitate long form activation via PKA phosphorylation [17] and homodimerization [18,19]. The LR2 in the super-short splice variant PDE4A4 has been shown to bind SRC tyrosyl kinase LYN though an SH3 domain [20]. PDE4 short, super-short, and truncated super-short splice variants have unique amino termini and differ from long forms by the exclusion in entirety of UCR1 (i.e., in short, super-short, and truncated super-short forms), and segments of UCR2 (in super-short and truncated super-short forms). These variations affect how PDE4 splice variant forms are regulated by eliminating UCR1-mediated dimerization and PKA-dependent phosphorylation, providing a functional dichotomy between the different PDE4 splice variant forms.

The PDE4 proteome is expansive, from four gene isoforms there are greater than twenty known PDE4 splice variants that have been identified in various mammals (primarily rats, mice and humans). In this study we first established the timing of PDE4 gene and splice variant form evolution in vertebrates using NCBI EST and ENSEMBL genome databases. We then identified evolutionary conserved splice variants (based on amino terminus conservation) within the vertebrate lineage that may likely form the functional foundation of PDE4 mediated cAMP degradation.

## Results & Discussion

### Evolutionary Conservation of the metal-dependent phosphohydrolase domain in PDE4 Splice Variants

We identified 162 PDE4 ESTs from 15 species encompassing the vertebrate phyla and one invertebrate (Additional File 1). All PDE4 s share high sequence identity in the catalytic domains. Residues H361, H397, D398, and D479, are found within the 'HD' motif of the phosphohydrolase domain and are known to confer PDE4 activity. These residues are invariant from Ciona to Humans (Additional File 2), with the exception of D479 (which is an asparagine in Ciona). This suggests that all PDE4 s included in our study are catalytically active.

### PDE4 splice variant classification

In order to classify newly identified PDE4 splice variants obtained from NCBI and ENSEMBL database screens into the proposed PDE4 classification system and verify previously annotated PDE4 splice variant classifications, a phylogenetic tree displaying the relationships of all splice variants to each other was constructed (based on the catalytic domain) for all PDE4 splice variants used in this study (Additional File 3).

**Figure 2:**
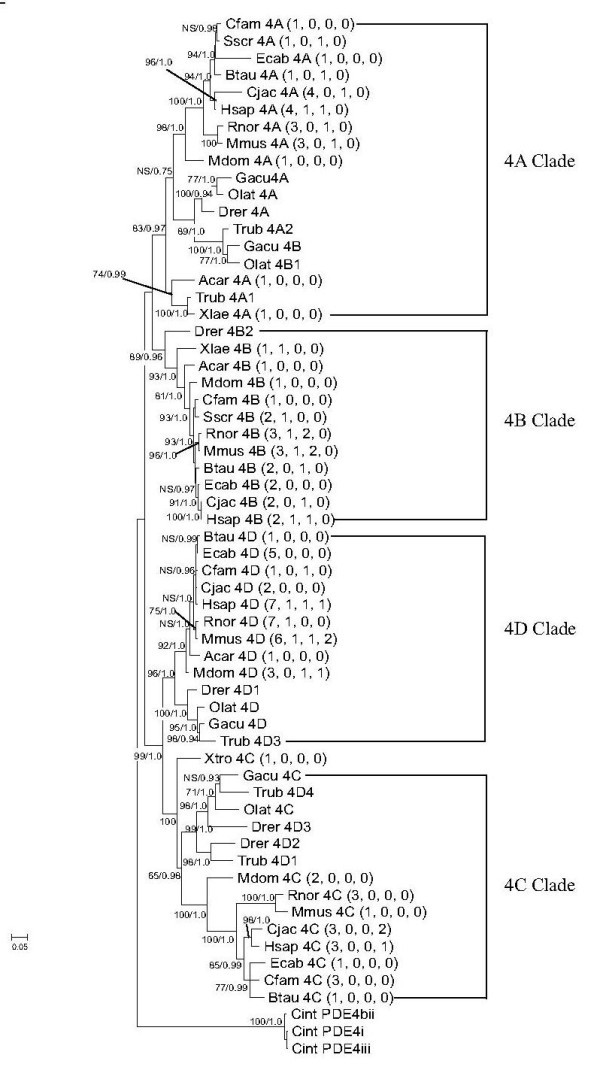
**PDE4 gene phylogeny**. Maximum likelihood and Bayesian phylogenetic trees constructed using amino acid sequences from UCR-1, UCR-2 and the catalytic domain from each PDE4 gene isoform of each taxa. Branch support following 1000 replicates is shown in bootstrap values at each divergence point. The trees were rooted with *Ciona intestinalis*. Maximum likelihood bootstrap values (top) and Bayesian posterior probabilities (below) are indicated at each node. Taxa are represented by the first letter of the genus name and first three letters of species name. NS = not supported. The number of splice variants for each gene isoform are indicated in parenthesis as; (long, short, super-short, and truncated super-short forms), respectively. Each PDE4 gene isoform clade is indicated by brackets.

All PDE4 splice variants segregate into four distinct subclades (Additional File 3), representative of the four known PDE4 gene isoforms. Surprisingly, teleost transcripts form two subclades for *PDE4A*, *PDE4B*, and *PDE4C *gene isoforms (Additional File 3), (Figure 2). In all PDE4 clades, rodents (i.e., mouse and rat) group out from other mammals. Several teleost PDE4 splice variants have been mis-annotated including; Stickleback PDE4B (ENSGACT00000025861) and Medaka PDE4B1 (ENSORLT00000012530) which group into the "*PDE4A*" clade, Fugu PDE4D4 (ENSTRUT00000034690), PDE4D1 (ENSTRUT00000034687), and Zebrafish 4D2 (XM_688538) and 4D3 (XM_001332019), all group into the "*PDE4C*" clade (Additional File 3, Additional File 1). Several un-annotated PDE4 splice variants were also identified. These newly identified NCBI ESTs and BLASTP proteins include: *X. tropicalis *PDE4C (BC129020), *E. caballus *PDE4A1 (cDNA acc. # XR_044570), *R. norvegicus *4A (protein acc. # EDL78317), *M. musculus *4A (protein acc. # EDL25164) and 4 D (protein acc. # CAQ51655), *H. sapiens *PDE4C (ENSP00000336624). Splice variants grouped according to PDE4 gene isoform type and not splice variant form (Additional File 3), because all isoform splice variants share the same exon pool (i.e. catalytic domain exons). The incorporation of all splice variants (long, short, super-short, truncated super-short) in our analysis allowed the detection of splice variant NCBI database transcript isoform mis-annotation and classification of newly discovered transcripts from NCBI EST libraries.

In this study, the multi-species alignments show several orthologous PDE4 splice variants that have different nomenclature designations either assigned automatically by computational annotation or through the literature. As rodents are typically the preferred animal models for studies of human pathologies our analyses in this section are limited to mice and rats, however several mis-annotations occur in other species as well. In the *PDE4A *gene, several orthologous splice variants have been given different designations between human and rodent in nomenclature (Additional File 4). These splice variants include the human long form PDE4A1 (NP_001104777) which has a homologous amino terminus to mouse PDE4A5 (NP_899668) and the rat PDE4A4 (NP_037233). The human long form PDE4A3 has two designations; PDE4A3 (NP_001104779) and PDE4A10 (AAD34217), and has a homologous amino terminus to mouse and rat PDE4A10 (EDL25164 and ENSRNOP00000057814, respectively). The human super-short form PDE4A4 (NP_006193) is homologous to mouse PDE4A1 (NP_062772) and rat PDE4A1 (AAA56859). Several of the homologous PDE4 D amino termini have also been assigned different nomenclature designations in human and rodent (Additional File 4). In the PDE4 D long forms, human PDE4D8 (AF53677) is homologous to mouse (ENSMUSP00000119583) and rat PDE4D4 (NM_001113334). Human PDE4D7 (AF536976) is homologous to rat PDE4D2 (NM_001113329). As individual PDE4 splice variants likely serve discrete and highly localized functions, it is important to remain consistent and accurate when referring to each splice variant. For the purposes of this study we refer to PDE4 splice variants in reference to the human ortholog if present.

### PDE4 Gene Isoform Evolution

The PDE4 phylogeny was constructed from one representative long form splice variant sequence for each of our 17 vertebrate species (Additional File 3, Additional File 1) and one invertebrate (*Ciona intestinalis*). All teleost long form transcripts were incorporated, as the catalytic domain phylogeny (Additional File 3) suggested the presence of two teleost *PDE4A*, *PDE4B*, and *PDE4C *clades. Within the long form PDE4 phylogeny four distinct gene types were again present in all vertebrates sampled (except *A. carolinensis *PDE4C) (Figure 2). PreENSEMBL genome assembly and PCR screens of reverse transcribed mRNA (data not shown) from the sea lamprey (*P. marinus*) suggest more than one PDE4 gene type is present in this species. *Ciona intestinalis *(outgroup) root placement established two main clades composed of *PDE4A *and *PDE4B*, and PDEs *4C *and *4 D *genes present in vertebrates studied (Figure 2) (Additional File 3). ENSEMBL genome database screens for PDE4 s show all teleosts tested (*D. rerio, O. lapites, G. aculeatus*, and *T. rubripes*) contain two *PDE4B *and *PDE4C *genes, however only *O. lapites, G. aculeatus, and T. rubripes *contain two *PDE4A *genes (Additional File 3, Additional File 1). PDE4 gene duplication occurred after the divergence of primitive chordates (Myxiniformes) and before teleost divergence to form present day PDE4 gene isoforms A-D (Figure 2), suggesting the expansion of this gene family and fixation of four distinct PDE4 isoforms occurred early in vertebrate evolution. After PDE4 gene duplication to form the four present day gene isoforms, teleosts appear to have undergone additional gene duplications detectable in *PDE4A*, *PDE4B*, and *PDE4C *genes. The symmetrical tree topology follows the (2R) hypothesis of Ohno [21,22], indicating that present day PDE4 gene number may be derived from two rounds of genome duplication occurring early in the vertebrate lineage. Although many genes are lost after genome duplication events, the homeostatic necessity for, and phylogenetic maintenance of the PDE4 genes attests to the importance of this gene family.

### Evolutionary Perspectives on PDE4 Splice Variant Form Genesis

**Figure 3:**
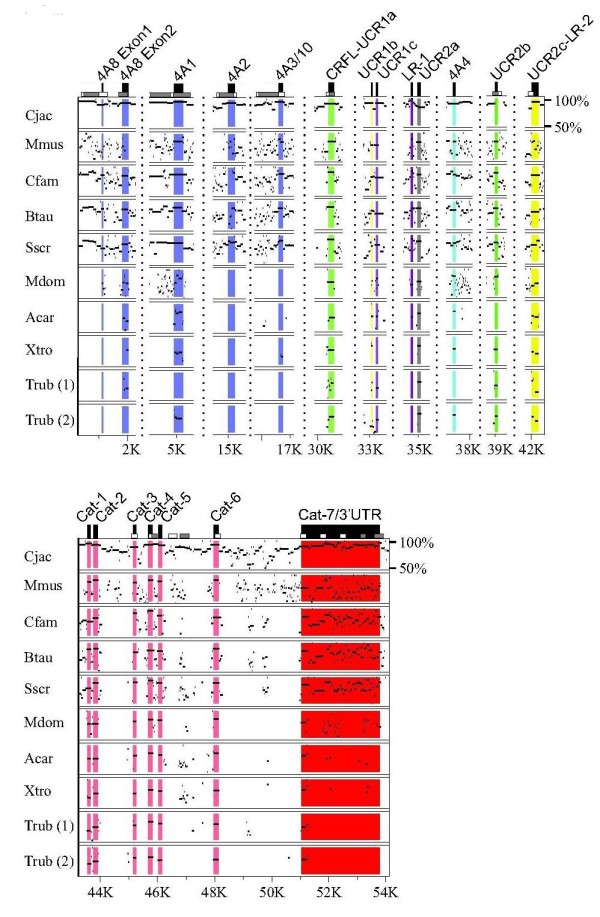
**Nucleotide Percent Identity Plot (PIP) for the PDE4A gene isoform**. PDE4A gene sequences from multiple species were aligned to human PDE4A using MultiPIP maker. Both gene duplicates are shown for the Trub PDE4A gene isoform. PIP output files were cropped to condense the alignment and display only the exons of interest. The 5' exons for the PDE4A long form splice variants PDE4A8, PDE4A1, PDE4A2, PDE4A3/10 are colored blue, while the super-short form PDE4A4 5' exon is colored cyan. Vertical dotted lines represent breaks in the nucleotide sequence, and nucleotide positions as referenced to Hsap PDE4A along the bottom scale. The range of nucleotide percent identity displayed (between 50% and 100%) is indicated in the upper right corner of the plot.

**Figure 4:**
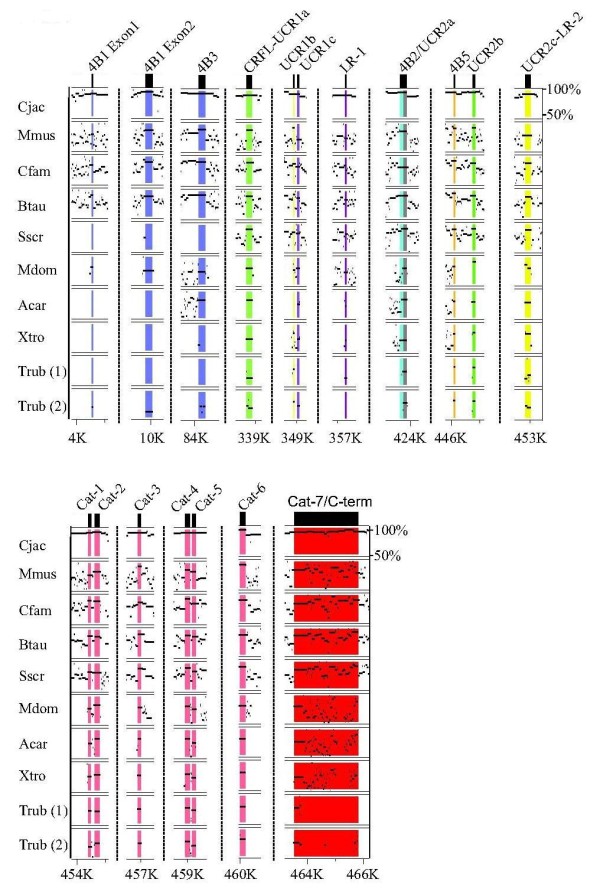
**Multi-species nucleotide PIP for the PDE4B gene**. PDE4B gene sequences from multiple species were aligned to human PDE4B using MultiPIP maker. Both duplicates are shown for the Trub PDE4B gene isoform. PIP output files were cropped to display only the exons of interest. The 5' exons for the PDE4B long forms PDE4B1 and PDE4B3 are colored blue, the short form PDE4B2 is colored cyan, and the super-short form PDE4B5 is colored orange. Vertical dotted lines represent breaks in the nucleotide sequence, and nucleotide positions as referenced to Hsap PDE4B along the bottom scale. The range of nucleotide percent identities shown (between 50% and 100%) is indicated in the upper right corner of the plot.

**Figure 5:**
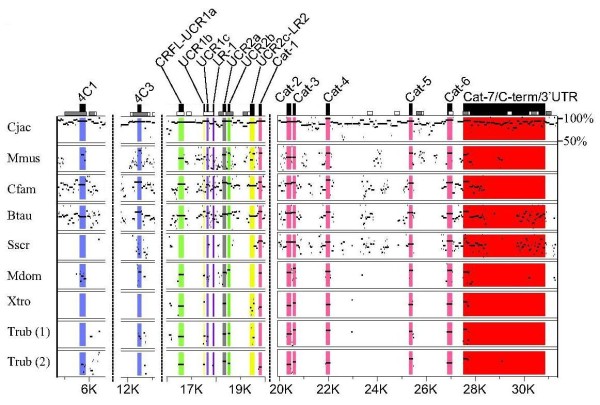
**Multi-species nucleotide PIP for the PDE4C gene**. PDE4C gene sequences from multiple species were aligned to human PDE4C using MultiPIP maker. Both gene duplicates are shown for the Trub PDE4C gene isoform. PIP output files were cropped to display only the exons of interest. 5' exons for PDE4C long form splice variants PDE4C1 and PDE4C3 are colored blue. Non-long form splice variant exons were not identified in this study. Vertical dotted lines represent breaks in the nucleotide sequence, and nucleotide positions as referenced to Hsap PDE4C along the bottom scale. The range of nucleotide percent identities shown (between 50% and 100%) is indicated in the upper right corner of the plot.

**Figure 6:**
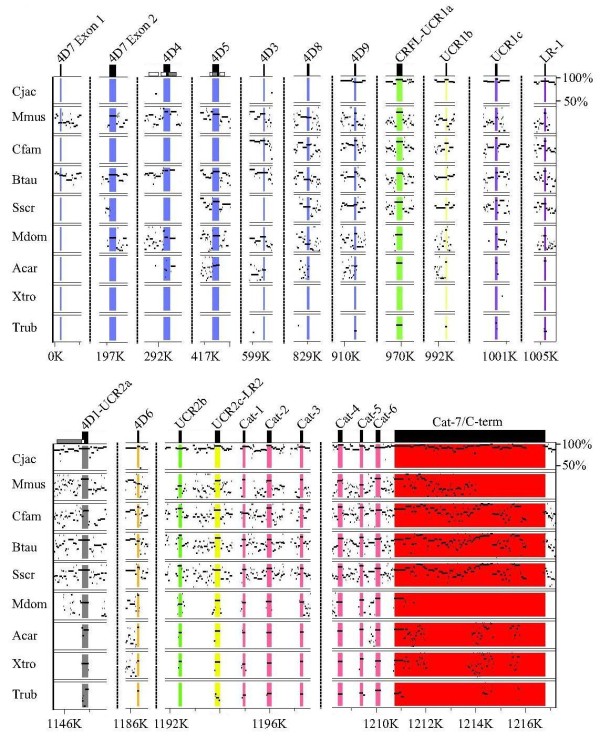
**Multi-species nucleotide PIP for the PDE4 D gene**. PDE4 D gene sequences from multiple species were aligned to human PDE4 D using MultiPIP maker. PIP output files were cropped to display only the exons of interest. 5' exons for PDE4 D long forms PDE4D7, PDE4D4, PDE4D5, PDE4D3, PDE4D8, and PDE4D9 are colored blue, the short form PDE4 D is colored gray, the super-short form PDE4D6 is colored orange. Vertical dotted lines represent breaks in the nucleotide sequence, and nucleotide positions as referenced to Hsap PDE4 D along the bottom scale. The range of nucleotide percent identities shown (between 50% and 100%) is indicated in the upper right corner of the plot.

The intron-exon structure of each PDE4 gene is conserved between isoforms and across species. An exception was found in *A. carolinensis*, where the *PDE4C *gene isoform was not detectable. The exons encoding for the amino-termini of each splice variant form (long, short, super-short) are located in tandem within intronic regions between the same domain junctions for all species examined (Figures. 1, 3, 4, 5, 6). PDE4 long form 5' exons are all located upstream of the CRFL-UCR1a exon, short form 5' exons are between the LR-1 exon and UCR2a exon. The super-short 5' exons are all found between the UCR2a and UCR2b exons. The amino termini of truncated super-short forms are initiated at an internal Methionine in the UCR2 region (Additional File 2, residue number 174), in the species examined. PDE4 long form splice variants from all four gene isoforms were detected in all organisms (*C. intestinalis *through mammals, with the exception of *A. carolinensis *PDE4C) tested using Ensembl and NCBI EST databases (Figure 2). PDE4A super-short form splice variant transcripts were detectable in EST libraries of *S. scrofa*, *B. taurus, R. norvegicus, M. musculus, C. jacchus*, and *H. sapiens *(Figure 2, Additional File 1). Amino termini corresponding to super-short form splice variants were only detectable in the *PDE4A *genes of *S. scrofa *through *H. sapiens*. Neither short nor truncated super-short PDE4A transcripts were identified from EST libraries screened. Amino termini corresponding to a short form transcript were detectable in the *PDE4B *genes of Xenopus (NP_001072863) through humans (NP_001032416), but not in teleosts. PDE4B super-short form transcripts were detectable in EST libraries from teleosts (ENSTRUP00000002631) through humans (EF595686). PDE4B truncated super-short forms were not detectable in any EST libraries. PDE4C short and super-short transcripts were not detectable in any EST libraries screened. A PDE4C truncated super-short form splice variant transcript and genomic amino terminus exon were identified in both *C. jacchus *(ENSCJAP00000025959) and *H. sapiens *(ENSP00000336624), but absent in non-primates analyzed in this study (Additional File 1).

Transcripts from the *PDE4 D *gene isoform for each of the short, super-short, and truncated super-short forms were identified in organisms ranging from rodents to humans. The disparities in timing and type of splice variant form generation (Figure 2) are likely due to differences in the formation the 5' exons and their corresponding alternative transcription start sites. Genomic analysis of orthologous gene regions performed between taxa indicated that transcription start sites and 5' exons within each PDE4 gene isoform developed through sequence drift (Additional File 5). Upon incorporation by sequence drift, all derived taxa encode the amino termini (Figures 3, 4, 5, 6).

The intracellular targeting and microdomain diversity of the PDE4 family suggests a functional expansion through the incorporation of different splice variant forms (long, short, super-short truncated super-short), as well as the alternative splicing of multiple splice variant 5' exons from each PDE4 gene isoform (observed in mammalian PDE4s). There is very limited evidence of alternative splicing in teleost PDE4 genes. Only one alternative splicing event (PDE4B super-short) (Additional File 1) is detectable in teleosts. Teleost-specific PDE4 gene duplications (in *PDE4A*, *PDE4B*, and *PDE4C *genes) and paralogous gene conservation increased the number of long forms for PDE4A,4B, and 4C gene isoforms in *D. rerio*, and *G. aculeatus *(*O. latipes *PDE4A and 4C) through an increase in gene dosage, in lieu of long form alternative splicing. Extensive PDE4 alternative splicing is limited in non-mammalian ESTs and does not appear to be a common property of all vertebrate PDE4 gene isoforms.

PDE4 gene isoforms are both alternatively spliced (primarily in mammals) and duplicated (in teleosts), both of which can increase PDE4 proteomic diversity. In a recent study, a correlation was detected between the size of the gene family and frequency of alternative splicing events, as duplicated gene families had significantly fewer alternative splicing events than single copy or less duplicated gene families [23]. This same trend can be observed in the PDE4 family (Figure 2).

The mammalian evolution of each of the PDE4 splice variant forms (long, short, super-short, truncated super-short) appears to have occurred at the same domain junctions independently within each gene isoform (Figure 2). The loss of UCR1 (in short, super-short, truncated super-short forms) as well as segments of UCR2 (in super-short and truncated super-short) may alter PDE4 enzyme conformational states and therefore catalytic activity [17,24,25]. Furthermore, the MAP kinase ERK1/2 has been shown to activate the PDE4 short form PDE4B2, whereas this phosphorylation event inhibits catalytic activity in the PDE4 long forms PDE4B1 and PDE4C2 [26], drawing a possible functional dichotomy that is largely based on the presence or absence of UCR1. While the evolutionary and functional necessity for non-long form PDE4 splice variants remains unclear, our analysis suggests they likely have a distinct role, unique from long form splice variants in the hydrolysis of cAMP, which is directly linked to the loss of UCR1 and UCR2 domains and may not be entirely related to the incorporation of variable amino termini.

### Conservation of alternatively spliced amino-termini exons in PDE4 gene isoforms

Four long forms and one super-short form splice variant from *PDE4A *are conserved across multiple species. The amino terminus of the *PDE4A *long form PDE4A8 is composed of two exons, both of which are conserved in the genomes of *S. scrofa, B. taurus, C. familiaris*, and *C. jacchus*, while these exons are not well conserved in *M. musculus *(Figure 3). EST searches for PDE4A8 in *S. scrofa *and *C. jacchus *identified splice variants orthologous to PDE4B1, but not orthologs to PDE4A8. A human PDE4A1 long form splice variant ortholog is detectable in the transcriptomes of *D. rerio*, *T. rubripes M. domestica*, *S. scrofa*, *C. familiaris*, *R. norvegicus*, *M. musculus*, and *C. jacchus*. The corresponding amino acid sequence ranges from 61% (*D. rerio*) to 98% (*C. jacchus*) sequence homology. The 5' exon corresponding to this amino terminus (PDE4A1) is conserved in the genomes of species from *T. rubripes *to *H. sapiens *(Figure 3). A *PDE4A *long form splice variant orthologous to *H. sapiens *PDE4A2 is conserved in the genomes of *C. jacchus *and *S. scrofa *(Figure 3) (Additional File 1). The amino terminus of this splice variant (PDE4A2) is much less conserved in *M. musculus*, which has 51% sequence identity at the amino acid level between rodents and primates (not shown). The PDE4A2 splice variant has EST support in both *M. musculus *and *C. jacchus*. The human PDE4A3/10 ortholog is detectable in the transcriptomes of *B. Taurus*, *R. norvegicus*, *M. musculus*, and *C. jacchus *(Additional File 1), with 80-89% amino acid sequence identity (not shown). The 5' exon corresponding to this amino terminus is detectable in mammalian species from *S. scrofa *to *H. sapiens *(Figure 3), Transcripts corresponding to the human PDE4A4, a super-short splice variant were detected in *B. Taurus*, *R. norvegicus*, *M. musculus*, and *C. jacchus *with the corresponding amino acid sequence sharing 100% sequence identity. The 5' exon encoding this amino terminus was detected in the genomes of *S. scrofa *to *H. sapiens*. There is also high sequence identity for the PDE4A4 5' exon in *X. tropicalis*, *A. carolinensis*, and *T. rubripes*, though the 5' flanking region (i.e., promoter region) shares little sequence identity in these species (not shown).

Two long forms, one short form and one super-short form splice variant(s) from the *PDE4B *gene isoform have both detectable EST transcript and genomic conservation across multiple species (Figure 4), (Additional File 1). Long form splice variant transcripts, orthologous to human PDE4B1, can be found in *G. aculeatus*, *O. latipes*, *T. rubripes*, *S. scrofa*, *E. caballus*, *R. norvegicus*, *M. musculus*, and *C. jacchus*, with the amino acid sequence identity ranging from 94% among mammals (*S. scrofa*), to 25% in teleosts (*O. latipes*). Two exons encode this amino terminus (PDE4B1), each of which were found in the genomes of these same species. Transcripts for the second *PDE4B *long form splice variant, orthologous to *H. sapiens *PDE4B3, were detectable in transcriptomes of *X. laevis*, *S. scrofa*, *B. taurus*, *M. domestica*, *E. caballus*, *R. norvegicus*, *M. musculus*, and *C. jacchus *(Figure 4), (Additional File 1), with the amino acid sequence identity ranging from 81-98% from *X. laevis *to *C. jacchus *(not shown). The 5' exon comprising this amino terminus was found in the genomes of all species ranging from teleosts to humans (Figure 4). One short form *PDE4B *transcript orthologous to human PDE4B2 was detectable in *X. tropicalis*, *S. scrofa*, *R. norvegicus*, and *M. musculus *(Additional File 1). The 5' exon encoding this amino terminus is conserved from *X. tropicalis *to *H. sapiens*, with the corresponding amino acid sequence ranging from 69-89% identity (not shown). A *PDE4B *super-short form orthologous to human PDE4B5 has both EST and genomic conservation from *T. rubripes *to *H. sapiens*, (Figure 4) (Additional File 1). The amino terminus of this splice variant is 100% identitical (in amino acid sequence) across species (not shown).

*PDE4C *is the smallest PDE4 gene isoform, and also contains the fewest total (two) conserved splice variants (Figure 5), (Additional File 1). Transcripts for the long form PDE4C1 were found in *C. familiaris*, *E. caballus*, *C. jacchus*, and *H. sapiens*, with amino acid sequence identity ranging from 55-86% (not shown). Genomic sequence conservation of the PDE4C1 splice variant 5' exon was found in *M. domestica*, *B. Taurus*, *C. familiaris*, *C. jacchus*, and *H. sapiens*. The 5' exon sequence encoding this amino terminus (PDE4C1) is not intact in *T. rubripes*, and *M. musculus *(Figure 5). Transcripts for the second conserved long form splice variant, orthologous to human PDE4C3, were detectable in *B. taurus*, *C. jacchus*, and *H. sapiens*, with 91-97% amino acid sequence identity (not shown). Genomic conservation for the 5' exon of PDE4C3 was found in *B. taurus*, *C. familiaris*, *M. musculus*, *C. jacchus*, and *H. sapiens*. *T. rubripes *and *M. domestica *lack an ATG start site (*M. domestica *and *T. rubripes*) and 5' sequence identity (*T. rubripes*) for this amino terminus.

The *PDE4 D *gene isoform contains the greatest number of orthologous 5' exons in the PDE4 family. Long form amino termini exons identified with both genomic conservation and strong EST support are orthologous to *H. sapiens *PDE4D3, 4, 5, 7, 8 and 9 (Figure 6), (Additional File 1). With the exception of the long form PDE4D9, which has EST support (72-100% amino acid sequence identity) and genomic conservation from *T. rubripes *to *H. sapiens*, PDE4 D long form splice variant conservation is found primarily in mammals. PDE4 D long forms orthologous to human PDE4D3, 4D4, 4D7, and 4D8 all have EST support in *B. Taurus *and *M. musculus *and range from 80-100% amino acid sequence identity. The exons encoding these long form amino termini are detectable in several mammalian species (i.e., *M. musculus*, *B. Taurus*, *M. domestica*). The PDE4 D gene is the only PDE4 gene isoform with detectable transcripts for short (orthologs to *H. sapiens *PDE4D1), super-short (orthologs to *H. sapiens *PDE4D6), and truncated super-short (orthologs to *H. sapiens *PDE4D2) splice variant forms. Both the short form PDE4D1 5' exon and truncated super-short form PDE4D2 have EST evidence and genomic conservation in *R. norvegicus *and *H. sapiens *(Figure 6), (Additional File 1). The super-short form orthologous to human PDE4D6 has EST support in *M. domestica*, *C. familiaris *and *M. musculus *with 95-97% amino acid sequence identity. Genome conservation of the 5' exon for this splice variant (PDE4D6) was found in *T. rubripes*, *X. tropicalis*, *A. carolinensis*, *M. domestica*, *S. scrofa*, *B. taurus*, *C. familiaris*, *M. musculus*, and *C. jacchus*. Splice variants orthologous to human PDE4D6 and PDE4B5 have homologous amino termini to each other (i.e., 4D6 and 4B5 have the same amino termini amino acid sequence).

Our multi-species PDE4 gene isoform alignments performed using PipMaker and transcript presence determined by NCBI EST database screens showed the evolutionary conservation of select PDE4 splice variant amino termini across species. Conservation across multiple species has been recognized as a key indicator of functional importance [27,28], although PDE4 s are known to be temporally and spatially differentially regulated [14,29,30]. Thus the lack of PDE4 EST support does not necessarily suggest splice variant absence. As such, the genomic conservation of splice variant amino termini were also utilized to infer evolutionary conservation across a wide range of species and confirm the timing of PDE4 splice variant evolution inferred from transcript presence or absence. Here we have identified several PDE4 splice variants from the gene isoforms that are conserved between multiple species clades. A relatively few of these splice variants have been well described and functionally characterized; among these include PDE4A4, PDE4D3, and PDE4D5. The super-short form PDE4A4 has been shown to interact with SH3-domains of the tyrosyl kinases Lyn [20]. This splice variant (PDE4A4) also potentially allows cross-talk between cAMP, phospholipase D, and calcium signaling [31]. The long form PD4D3 has been shown to interact with the A-kinase anchoring protein (mAKAP) [32,33] and has also been suggested to serve a role in ryanodine receptor function and Ca^2+ ^"leakiness" from the sarcoplasmic reticulum (SR) during heart failure [34]. The long form PDE4D5 is perhaps the best documented PDE4, and this splice variant forms a complex with β-arrestin and serves to regulate β_2 _adrenoreceptor activity [7,35,36]. These three examples (PDE4A4, 4D3 and 4D5) represent a very small proportion of evolutionarily conserved PDE4 splice variants. The functional characterization of other conserved splice variants shown in our study may also prove important in vertebrate cAMP signaling pathways.

### The Splice Variants PDE4B5 and PDE4D6 Share Homologous Amino Termini

The 5' exon nucleotide sequences of the super-short splice variants from two different gene isoforms (PDE4B5 and PDE4D6) share high nucleotide sequence identity (77-100% between vertebrates sampled) (Additional File 6). Amino acid sequences ranged from 87-100% identity among vertebrates (Additional File 6: Supplemental Figure S4C). ESTs and/or genomic detection of the 4B5 5' exon were found from teleosts to humans (Figure 4). In contrast, ESTs for PDE4D6 were identified for *C. familiaris *to *H. sapiens*, though genome conservation for the 5' exon of PDE4D6 extends to teleosts (Figure 6), (Additional File 6: Supplemental Figure S4B). Lower vertebrate (basal to *C. familiaris*) 4D6 upstream promoter regions appear to not have "TATA" and "GC" motifs, necessary for transcriptional initiation (Additional File 6: Supplementary Figure 4A).

The splice variants PDE4B5 and PDE4D6 appear to represent an exception to the rule among PDE4 splice variant amino termini; they have homologous amino termini (Additional File 6: Supplemental Figure S4C), and are co-expressed in the brain [37,38]. The 5' exons for these super-short form splice variants appeared in their respective gene isoforms (*PDE4B *and *PDE4D*) early in vertebrate evolution (Figures 4, 6). The difference in the timing of PDE4B5 and 4D6 transcript appearance in the vertebrate lineage, is likely due to the relatively delayed development of the PDE4D6 5' flanking sequence and promoter region in teleosts (i.e., *T. rubripes*) as compared to mammals (i.e., *C. familiaris*) (Additional File 6: Supplemental Figure S4A). The "TATA" or Hogness-Goldberg box, is generally located 20-50 bp upstream of the transcription start point and controls the transcription start point. A consensus TATA box was found upstream of the initiation codon for PDE4D6 in *H. sapiens *and *C. familiaris*, but not in *M. domestica *and *T. rubripes *(Additional File 6: Supplemental Figure S4A). Consensus GC-boxes, which control the initial binding of RNA polymerase, were identified in *H. sapiens *and *C. familiaris *but not in *M. domestica *and *T. rubripes*. While neither element is uniquely essential for promoter function, the correlation of the development of these promoter elements with the appearance of PDE4D6 ESTs suggests sequence drift in the promoter region is responsible for transcription of this splice variant in mammals more derived than *M. domestica*. Detailed biochemical information regarding the protein-protein interactions of PDE4B5 and PDE4D6 are lacking, and it is unclear if this represents a case of redundancy in the PDE4 family or a specialized localization (conferred by the amino terminus) in different areas of the brain.

### Conservation and Functionality in PDE4 Regulatory Domains

In contrast to the PDE4 splice variant 5' exons (amino termini), coding regions found in the LR1, LR2, and terminal catalytic domain exons are highly conserved across species (Figures 3, 4, 5, 6) (Additional File 2). LR-1 is highly conserved (teleosts to humans for *PDE4A*, *PDE4B*, and *PDE4D*; placental mammals for PDE4C) within gene isoforms (Additional File 2). LR-2 is also only evolutionarily conserved within gene isoforms (Additional File 2). LR2 is conserved in placental mammals, tetrapods, and vertebrates for *PDE4A*, *PDE4B*, and *PDE4 D *respectively. The carboxy terminal region of the PDE4C LR2 is conserved in vertebrates, while the amino terminal region is conserved in placental mammals. Located in the carboxy-terminal region of all PDE4 splice variants, ERK2 binding domain and phosphorylation are highly conserved between and among PDE4 gene isoforms across the vertebrate lineage (Additional File 2). Minor residue variations are detectable in the carboxy-terminus of *X. laevis *4B, and *M. musculus*, *C. familiaris*, and *S. scrofa *PDE4Cs (Additional File 2).

Evolutionarily conserved motifs between diverse taxa are indicative of functional importance, and several such motifs (e.g., PKA phosphorylation of UCR1, and ERK1/2 phosphorylation of the carboxy-terminus) have been identified and described in PDE4 s [17,26,39]. We observed previously unidentified evolutionary conservation within LR1, LR2 and the c- terminus among PDE4 s that are likely indicative of functional importance. The serine-threonine kinase Erk2 has been shown to both inhibit the long form splice variants PDE4D3, PDE4B1 and PDE4C2 and activate the short forms splice variants PDE4B2 and PDE4D1 [26,39]. ERK phosphorylation of a PDE4A recombinant fragment has been demonstrated [40], however ERK-mediated phosphorylation of native *PDE4A *splice variants has not been shown to date. The observed conservation at this site across all isoforms strongly suggests ERK regulation within PDE4A splice variants as well (Additional File 2). ERK phosphorylation at this site appears to be an ancient form of PDE4 regulation, as the consensus motif is found from *C. intestinalis *to *H. sapiens*. We also observed more recent development of evolutionary conservation within the PDE4A LR2. This region has been shown to interact with the SRC tyrosyl kinase LYN through an SH3 domain in humans [20]. This highly conserved region appears to have been inserted in placental mammals (Additional File 2), increasing the potential mechanisms of PDE4A regulation. This poly-proline domain is absent from the rodent *PDE4*A LR2 region (Additional File 2) signifying a functional divergence between rodents and other mammals in this protein region and is consistent with the general rodent-mammalian divergence observed in PDE4 gene evolution (Figure 2). The LR2 in *PDE4B*, *PDE4C*, and *PDE4 D *are also highly conserved within gene isoform types, and thus may suggest an as of yet unidentified ancient functional role. LR1 is also highly conserved within PDE4 gene isoforms, but less so between gene isoforms. The functional significance of LR1 is unknown, however the high conservation observed within isoforms suggests a likely functionality related to PDE4 gene isoform-specific sequence identity.

## Conclusions

In summary, this phylogenetic analysis of PDE4 gene isoforms and alternative splicing has addressed several gaps in our understanding of the evolutionary basis for the functional diversity seen in the multitude of PDE4 splice variants. Following the divergence of invertebrates (*C. intestinalis*) and primitive vertebrates (teleosts), multiple gene duplications produced the present day PDE4 gene isoforms (A-D). The production of multiple protein products from these genes through alternative splicing is limited in primitive vertebrates. Integration of distinctive long form amino termini and expansion of splice variant type (short, super-short, truncated super-short) is largely introduced in tetrapods and greatly expanded in mammals. This growth in splice variant identity (unique amino termini) and different form types appears at different times throughout vertebrate evolution and occurs independently within each gene isoform. Sequence drift appears to be responsible for the development of PDE4 amino termini-coding exons, as well as promoter development.

The most physiologically distinguishing characteristic of PDE4 splice variants are the amino termini, and from each gene several splice variant amino termini have been highly conserved throughout evolution. These conserved splice variants likely represent the functional core of the expansive PDE4 proteome. In addition to the splice variant-specific amino termini, several regulatory domains (including PKA phosphorylation, long form dimerization and ERK phosphorylation) have remained essentially completely conserved. The conservation of these regulatory domains, which provide an additional level of functional regulation, underpins the importance of these domains in an evolutionary functional context.

## Methods

### Sequence Data Acquisition

PDE4 nucleotide and protein sequences were obtained from a search of NCBI http://www.ncbi.nlm.nih.gov and ENSEMBL http://www.ensembl.org genomic databases. The UCR-1, UCR-2, and catalytic domain nucleotide and amino acid sequences from human, rat and mouse PDE4A-D splice variants were used to identify annotated and unannoted PDE4 transcript and protein sequences in vertebrates and invertebrates (*Ciona intestinalis*) using NCBI BLASTP, TBLASTN, EST database searches and Ensembl (release 41). Select species, spanning the vertebrate lineage, used for this study include: mammals-*Homo sapiens (H. sap)*, *Callithrix jacchus (C. jac)*, *Mus musculus (M. mus)*, *Rattus norvegicus (Rnor)*, *Canis familiaris (C. fam)*, *Equus caballus (E. cab)*, *Bos Taurus (B. tau)*, *Sus scrofa (S. scr)*, *Monodelphis domestica (M. dom)*, reptiles- *Anolis carolinensis (A. car)*, amphibians- *Xenopus tropicalis (X. tro)*, teleosts-*Danio rerio (D. rer), Takifugu rubripes (T. rub), Oryzias latipes (O. lat), Gasterosteus aculeatus (G. acu)*; chordates- Sea lamprey *Petromyzon marinus (P. mar)*.

### Phylogenetic Analysis

PDE4 translated amino acid sequences were aligned using the Clustal function of Mega v4, according to the default settings. PDE4 splice variant relationships based on the catalytic domain were determined using the Neighbor Joining algorithm of Mega v4. The tree shown represents three independent runs, each with 1000 bootstrap replicates using the PAM matrix of amino acid evolution. One long form splice variant nucleotide sequence from each clade was selected for each species of interest (representative of species individual PDE4 gene isoforms, highlighted in Additional File 3), and translated in the clustal function of MEGA. Mr Bayes and PhyML were used to construct a vertebrate PDE4 gene family phylogeny using the amino acid sequences common to all PDE4 long forms (UCR-1, UCR-2, and catalytic domains). The tree represents three and four independent runs of 1000 bootstrap replicates and four million generations, in PhyML and Mr Bayes, respectively. Mr Bayes was allowed to estimate the best fixed rate model of protein evolution during the run by setting the prior for amino acid model to mixed. The best fixed rate model was Jones-Taylor Thornton (JTT) model (gamma = 0.946). PhyML analyses were run under this model as well.

### Identification of Promoter elements

Upstream flanking regions of the PDE4D6 5' exon were analyzed using TRANSFAC motif search [41]http://motif.genome.jp/, using a cutoff score of 85 for all vertebrate motifs.

### Exon conservation using Percent Identity Plots (PIP)

Multi PipMaker http://bio.cse.psu.edu[42,43] was used for PDE4 gene comparisons across vertebrate taxa with respect to exon presence/conservation. In each case, the human PDE4 gene of interest (PDE4A, PDE4B, PDE4C, or PDE4D) was used as the reference gene, in the forward orientation. Multi PIP maker offers an additional function in which user defined exon positions are displayed. Exon positions (coordinates) within each human PDE4 gene were determined using the human gene sequence and conserved splice variant amino termini, facultative (not present in all forms) and constitutive exons (present in all forms) within the two sequence BLAST (bl2seq) function from NCBI http://blast.ncbi.nlm.nih.gov/Blast.cgi and then formatted into an exon file for use in Multi PipMaker.

## Authors' contributions

All authors have read and approved the final manuscript. KRJ compiled sequence data, participated in data analysis, designed the study, conceived and drafted the manuscript. JNJ performed the phylogenetic analysis, designed the study, edited and helped design the manuscript. RSD conceived the project and provided input on manuscript drafting.

## Supplementary Material

Additional file 1 **Supplemental Table S1**. Supplemental Table S1 is a table listing the PDE4 Sequences used in this study. These sequences were obtained from a search of NCBI and ENSEMBL genome databases. Species abbreviations consist of the first letter of the genus, and first three letters of the species. Accession numbers for the mRNA and protein sequences are provided, along with the gene accession number. Click here for file

Additional file 2 **Supplemental Figure S1**. Protein Multiple Sequence Alignment Excluding the Amino Terminus From PDE4 gene isoforms. Supplemental Figure S1 is a clustalW alignment of the PDE4 proteins from all four gene isoforms. Highly conserved regions are the conserved region found in long forms (CRFL)-upstream conserved region 1 (UCR1) which contains a PKA phosphorylation motif from residues 36-39, linker region 1 (LR1), upstream conserved region 2 (UCR2) which contains the amino terminus for truncated super-short forms, linker region 2 (LR2), catalytic domain, which contains the metal-dependent phosphohydrolase motif from residues 356-609 and a Kinase Interaction Motif (KIM) from residues 484-497, and carboxy termini which contains an ERK phosphorylation site from residues 621-623 and "FQF" motif from 660-662. Click here for file

Additional file 3 **Supplemental Figure S2**. Phylogenetic classification of PDE4 Splice Variants Used in This Study. Supplemental Figure S2 is a Bayesian phylogenetic tree constructed using PDE4 catalytic domain amino acid sequences from each PDE4 gene isoform, from each taxa used in this study. Click here for file

Additional file 4 **Supplemental Table S2**. Supplemental Table S2 lists orthologous PDE4 splice variants with different designations in nomenclature. Three PDE4A and three PDE4 D splice variants have homologous amino termini but have different names between humans and rodents. Click here for file

Additional file 5 **Supplemental Figure S3**. PDE4 Exon Formation Through Sequence Drift. Supplemental Figure S3 is a multiple sequence alignment of flanking and coding nucleotide sequences orthologous to the H. sapiens splice variant PDE4A3/10, showing the formation of this exon occurred through sequence drift. Click here for file

Additional file 6 **Supplemental Figure S4**. PDE4 Splice Variant Promoter Formation Through Sequence Drift. Supplemental Figure S4 is a multiple sequence alignments of the PDE4D6 upstream flanking region, PDE4B5 and PDE4D6 5'exons, and corresponding amino acid sequences. Click here for file
