# Supplementary material for: An evolutionary analysis of cAMP-specific Phosphodiesterase 4 alternative splicing
Source: BMC Evol Biol. 2010 Aug 11;10:247. doi: 10.1186/1471-2148-10-247 (PMC2929239; doi:10.1186/1471-2148-10-247)
Supplement: Additional file 1 — Supplemental Table S1. Supplemental Table S1 is a table listing the PDE4 Sequences used in this study. These sequences were obtained from a search of NCBI and ENSEMBL genome databases. Species abbreviations consist of the first letter of the genus, and first three letters of the species. Accession numbers for the mRNA and protein sequences are provided, along with the gene accession number. [file 1471-2148-10-247-S1.DOC]

**SI Table 1.** PDE4 Sequences were obtained from a search of NCBI and ENSEMBL genome databases. Species abbreviations consist of the first letter of the genus, and first three letters of the species. Accession numbers for the mRNA and protein sequences are provided, along with the gene accession number. As indicated in the form classification, splice variants were grouped as long, short, super-short, or truncated super-short. If multiple ESTs were found, EST support was considered strong, and if the splice variant 5’ exon was identified in genomes of more than two species, the exon is considered to have multi-genome conservation. In cases where amino termini could not be determined, the designation “N.A.” is given for multi-species conservation

| **Species** | ***Splice Variant*** | Gene Accession Number | Form Classification | EST Support | Multi-Genome Conservation |
| --- | --- | --- | --- | --- | --- |
| **Cint** | ***PDE4i*** | ENSCING00000007423 |  |  |  |
| Transcript | ENSCINT00000015269 |  |  |  |  |
| Protein | ENSCINP00000015269 |  | Long | Strong | No |
|  | ***PDE4ii*** |  |  |  |  |
| Transcript | ENSCINT00000015277 |  |  |  |  |
| Protein | ENSCINP00000015277 |  | Long | Strong | No |
| **Gacu** | ***4A1*** | Gene 2 of 2: ENSGACG00000013825 |  |  |  |
| Transcript | ENSGACT00000018291 |  |  |  |  |
| Protein | ENSGACP00000018256 |  | Long | Weak | N.A. |
|  | ***4A2*** | Gene 1 of 2: ENSGACG00000019526 |  |  |  |
| Transcript | ENSGACT00000025861 |  |  |  |  |
| Protein | ENSGACP00000025810 |  | Long | Weak | Yes |
|  | ***4B1*** | Gene 2 of 2: ENSGACG00000016235 |  |  |  |
| Transcript | ENSGACT00000021460 |  |  |  |  |
| Protein | ENSGACP00000021419 |  | Long | Weak | N.A. |
|  | ***4B2*** | Gene 1 of 2: ENSGACG00000007263 |  |  |  |
| Transcript | ENSGACT00000009640 |  |  |  |  |
| Protein | ENSGACP00000009620 |  | Long | Weak | N.A. |
|  | ***4C1*** | Gene 1 of 2: ENSGACG00000008183 |  |  |  |
| Transcript | ENSGACT00000010856 |  |  |  |  |
| Protein | ENSGACP00000010833 |  | Long | Weak | N.A. |
|  | ***4C2*** | Gene 2 of 2: ENSGACG00000015515 |  |  |  |
| Transcript | ENSGACT00000020506 |  |  |  |  |
| Protein | ENSGACP00000020467 |  | Long | Weak | N.A. |
| **Species** | ***Splice Variant*** | Gene Accession number | Form Classification | EST Support | Multi-Genome Conservation |
|  | ***4D*** | Gene 1 of 1:  ENSGACG00000007191 |  |  |  |
| Transcript | ENSGACT00000009546 |  |  |  |  |
| Protein | ENSGACP00000009526 |  | Long | Weak | Yes |
| **Olat** | ***4A1*** | Gene 1 of 2: ENSORLG00000005439 |  |  |  |
| Transcript | ENSORLT00000006850 |  |  |  |  |
| Protein | ENSORLP00000006849 |  | Long | Strong | N.A. |
|  | ***4A2*** | Gene 2 of 2: ENSORLG00000009983 |  |  |  |
| Transcript | ENSORLT00000012530 |  |  |  |  |
| Protein | ENSORLP00000012529 |  | Long | Strong | Yes |
|  | ***4B1*** | Gene 1 of 2: ENSORLG00000010308 |  |  |  |
| Transcript | ENSORLT00000012920 |  |  |  |  |
| Protein | ENSORLP00000012919 |  | Long | Strong | N.A. |
|  | ***4B2*** | Gene 1 of 2:  ENSORLG00000010308 |  |  |  |
| Transcript | ENSORLT00000012922 |  |  |  |  |
| Protein | ENSORLP00000012921 |  | Super-Short | Strong | Yes |
|  | ***4B3*** | Gene 2 of 2: ENSORLG00000009796 |  |  |  |
| Transcript | ENSORLT00000012296 |  |  |  |  |
| Protein | ENSORLP00000012295 |  | Long | Strong | N.A. |
|  | ***4B4*** | Gene 2 of 2:  ENSORLG00000009796 |  |  |  |
| Transcript | ENSORLT00000012300 |  |  |  |  |
| Protein | ENSORLP00000012299 |  | Super-Short | Strong | Yes |
|  | ***4C1*** | Gene 1 of 1: ENSORLG00000008341 |  |  |  |
| Transcript | ENSORLT00000010476 |  |  |  |  |
| Protein | ENSORLP00000010475 |  | Long | Weak | No |
|  | ***4D1*** | Gene 1 of 1:  ENSORLG00000004643 |  |  |  |
| Transcript | ENSORLT00000005843 |  |  |  |  |
| Protein | ENSORLP00000005842 |  | Long | N.A. | N.A. |
| **Drer** | ***4A*** | Gene: 1 of 1 NCBI: LOC572061 |  |  |  |
| Transcript | XM_695720 |  |  |  |  |
| Protein | XP_700812 |  | Long | Strong | Yes |
|  |  |  |  |  |  |
|  |  |  |  |  |  |
| **Species** | ***Splice Variant*** | Gene Accession Number | Form Classification | EST Support | Multi-Genome Conservation |
|  | ***4B1*** |  |  |  |  |
| Transcript | XM_703863 | Gene: 1 of 2 NCBI:  LOC565706  ENSDARG00000032868 |  |  |  |
| Protein | XP_708955 |  | Long | Strong | No |
|  | ***4B2*** |  |  |  |  |
| Transcript | ENSDART00000085631 | Gene: 2 of 2  ENSDARG00000074233 |  |  |  |
| Protein | ENSDARP00000080066 |  | Long | Weak | No |
|  | ***4C1*** | Gene 1 of 2  ENSDARG00000013221 |  |  |  |
| Transcript | ENSDART00000002096 |  |  |  |  |
| Protein | ENSDARP00000012865 |  | Long | N.A. | N.A. |
|  | ***4C2*** | Gene 2 of 2  ENSDARG00000002411 |  |  |  |
| Transcript | ENSDART00000023820 |  |  |  |  |
| Protein | ENSDARP00000009455 |  | Long | N.A. | N.A. |
|  | ***4D1*** | Gene 1 of 1  LOC566998  ENSDARG00000032761 |  |  |  |
| Transcript | XM_690282 |  |  |  |  |
| Protein | XP_695374.3 |  | Long | Strong | No |
|  | ***4D2*** | Gene  LOC565259 |  |  |  |
| Transcript | XM_688538 |  |  |  |  |
| Protein | XP_693630 |  | Long | Strong | No |
|  | ***4D3*** | Gene  LOC792687 |  |  |  |
| Transcript | XM_001332019 |  |  |  |  |
| Protein | XP_001332055 |  | Long | Strong | No |
| **Trub** | ***4A1*** | Gene 1 of 2: ENSTRUG00000015440 |  |  |  |
| Transcript | ENSTRUT00000026107 |  |  |  |  |
| Protein | ENSTRUP00000026002 |  | Long | Weak | Yes |
|  | ***4A2*** | Gene 2 of 2: ENSTRUG00000010334 |  |  |  |
| Transcript | ENSTRUT00000039616 |  |  |  |  |
| Protein | ENSTRUP00000039475 |  | Long | Strong | Yes |
|  |  |  |  |  |  |
|  |  |  |  |  |  |
| **Species** | ***Splice Variant*** | Gene Accession Number | Form Classification | EST Support | Multi-Genome Conservation |
| **Trub** | ***4B1*** | Gene 1 of 2: ENSTRUG00000001133 |  |  |  |
| Transcript | ENSTRUT00000002643 |  |  |  |  |
| Protein | ENSTRUP00000002629 |  | Long | Strong | No |
|  | **4B2** | Gene 1 of 2: ENSTRUG00000001133 |  |  |  |
| Transcript | ENSTRUT00000002645 |  |  |  |  |
| Protein | ENSTRUP00000002631 |  | Super-Short | Strong | Yes |
|  | ***4B3*** | Gene 2 of 2: ENSTRUG00000018562 |  |  |  |
| Transcript | ENSTRUT00000047697 |  |  |  |  |
| Protein | ENSTRUP00000047536 |  | Long | Yes | N.A. |
|  | ***4C1*** | Gene 1 of 2: ENSTRUG00000011028 |  |  |  |
| Transcript | ENSTRUT00000027941 |  |  |  |  |
| Protein | ENSTRUP00000027831 |  | Long | Strong | N.A. |
|  | ***4C2*** | Gene 2 of 2: ENSTRUG00000006287 |  |  |  |
| Transcript | ENSTRUT00000015425 |  |  |  |  |
| Protein | ENSTRUP00000015356 |  | Long | Strong | N.A. |
|  | ***4D1*** | Gene 1 of 1:  ENSTRUG00000013545 |  |  |  |
| Transcript | ENSTRUT00000034687 |  |  |  |  |
| Protein | ENSTRUP00000034561 |  | Long | Strong | N.A. |
|  | ***4D2*** |  |  |  |  |
| Transcript | ENSTRUT00000034688 |  |  |  |  |
| Protein | ENSTRUP00000034562 |  | Long | Strong | N.A. |
|  | ***4D3*** |  |  |  |  |
| Transcript | ENSTRUT00000034689 |  |  |  |  |
| Protein | ENSTRUP00000034563 |  | Long | Weak | N.A. |
|  | ***4D4*** |  |  |  |  |
| Transcript | ENSTRUT00000034690 |  |  |  |  |
| Protein | ENSTRUP00000034564 |  | Long | Strong | N.A. |
| **Xtro** | ***4A*** | LOC495146 |  |  |  |
| Transcript | NM_001094841 |  |  |  |  |
| Protein | NP_001088310 |  | Long | Strong | No |
|  | ***4B1*** | MGC83972 |  |  |  |
| Transcript | NM_001092664 |  |  |  |  |
| Protein | NP_001086133 |  | Long | Strong | Yes |
|  |  |  |  |  |  |
|  |  |  |  |  |  |
| **Species** | ***Splice Variant*** | Gene Accession Number | Form Classification | EST Support | Multi-Genome Conservation |
| **Xtro** | ***4B2*** | MGC147384 |  |  |  |
| Transcript | NM_001079395 |  |  |  |  |
| Protein | NP_001072863 |  | Short | Strong | Yes |
|  | ***4C*** |  |  |  |  |
| Transcript | BC129020 | cDNA clone IMAGE:7685049 |  |  |  |
| Protein | No Protein Information |  | Long | Strong | N.A. |
|  | ***4D*** | ENSXETG00000018854 |  |  |  |
| Transcript | ENSXETT00000040889 |  |  |  |  |
| Protein | ENSXETP00000040889 |  | Long | Weak | N.A. |
| **Acar** | ***4A1*** | ENSACAG00000008734 |  |  |  |
| Transcript | ENSACAT00000008965 |  |  |  |  |
| Protein | ENSACAP00000008777 |  | Long | Strong | N.A. |
|  | ***4B1*** | ENSACAG00000010815 |  |  |  |
| Transcript | ENSACAT00000010881 |  |  |  |  |
| Protein | ENSACAP00000010660 |  | Long | Strong | N.A. |
|  | ***4D1*** | ENSACAG00000009088 |  |  |  |
| Transcript | ENSACAT00000009268 |  |  |  |  |
| Protein | ENSACAP00000009075 |  | Long | Strong | N.A. |
| **Cfam** | ***4A*** | ENSCAFG00000017790 |  |  |  |
| Transcript | ENSCAFT00000028214 |  |  |  |  |
| Protein | ENSCAFP00000026237 |  | Long | Weak | Yes |
|  | ***4B*** | LOC479540 (NCBI) |  |  |  |
| Transcript | XM_536678 |  |  |  |  |
| Protein | XP_536678 |  | Long | Strong | No |
|  | ***4C1*** | LOC609938 (NCBI) |  |  |  |
| Transcript | XM_847290 |  |  |  |  |
| Protein | XP_852383 |  | Long | Strong | No |
|  | ***4C2*** |  |  |  |  |
| Transcript | ENSCAFT00000023694 |  |  |  |  |
| Protein | ENSCAFP00000021997 |  | Long | Strong | Yes |
|  | ***4C3*** |  |  |  |  |
| Transcript | ENSCAFT00000023697 |  |  |  |  |
| Protein | ENSCAFP00000021999 |  | Long | Strong | Yes |
|  | ***4D1*** | LOC487221 (NCBI) |  |  |  |
| Transcript | ENSCAFT00000011395 |  |  |  |  |
| Protein | ENSCAFP00000010556 |  | Super-Short | Strong | Yes |
|  | ***4D2*** |  |  |  |  |
| Transcript | XM_544349 |  |  |  |  |
| Protein | XP_544349 |  | Long | Strong | No |
| **Species** | ***Splice Variant*** | Gene Accession Number | Form Classification | EST Support | Multi-Genome Conservation |
| **Sscr** | ***4A1*** | ENSSSCG00000013641 |  |  |  |
| Transcript | NM_001123159 |  |  |  |  |
| Protein | NP_001116631 |  | Super-Short | Strong | Yes |
|  | ***4A2*** |  |  |  |  |
| Transcript | ENSSSCT00000014899 |  |  |  |  |
| Protein | ENSSSCP00000014498 |  | Long | Strong | Yes |
|  | ***4B1*** | ENSSSCG00000003805 |  |  |  |
| Transcript | NM_001130019 |  |  |  |  |
| Protein | NP_001123491 |  | Long | Strong | Yes |
|  | ***4B2*** |  |  |  |  |
| Transcript | NM_001130018 |  |  |  |  |
| Protein | NP_001123490 |  | Short | Strong | Yes |
|  | ***4B3*** |  |  |  |  |
| Transcript | EU189937 |  |  |  |  |
| Protein | ABZ03973 |  | Long | Strong | Yes |
|  | ***4C*** | ENSSSCG00000013898 |  |  |  |
| Transcript | ENSSSCT00000015184 |  |  |  |  |
| Protein | ENSSSCP00000014778 |  | Fragment | Strong | N.A. |
|  | ***4D*** | ENSSSCP00000017937 |  |  |  |
| Transcript | ENSSSCT00000018436 |  |  |  |  |
| Protein | ENSSSCP00000017937 |  | Long | Strong | N.A. |
| **Btau** | ***4A1*** | ENSBTAG00000012032 |  |  |  |
| Transcript | NM_001101081 |  |  |  |  |
| Protein | NP_001094551 |  | Super-short | Strong | Yes |
|  | ***4A2*** |  |  |  |  |
| Transcript | BC118116 |  |  |  |  |
| Protein | AAI18117 |  | Long | Strong | N.A. |
|  | ***4B1*** | ENSBTAG00000008636 |  |  |  |
| Transcript | NM_001102546 |  |  |  |  |
| Protein | NP_001096016 |  | Long | Strong | Yes |
|  | ***4B2*** |  |  |  |  |
| Transcript | ENSBTAT00000043622 |  |  |  |  |
| Protein | ENSBTAP00000041181 |  | Long | Weak | N.A. |
|  | ***4B3*** |  |  |  |  |
| Transcript | BC133342 |  |  |  |  |
| Protein | AAI33343 |  | Super-short | Strong | Yes |
|  | ***4C*** | ENSBTAG00000010652 |  |  |  |
| Transcript | XM_602953 |  |  |  |  |
| Protein | XP_602953 |  | Long | Strong | Yes |
|  |  |  |  |  |  |
| **Species** | ***Splice Variant*** | Gene Accession Number | Form Classification | EST Support | Multi-Genome Conservation |
| **Btau** | ***4D*** | ENSBTAG00000000494 |  |  |  |
| Transcript | XM_588063 |  |  |  |  |
| Protein | XP_588063 |  | Long | Strong | Yes |
| **Mdom** | ***4A*** | LOC100012755 (NCBI) |  |  |  |
| Transcript | XM_001367083 |  |  |  |  |
| Protein | XP_001367120 |  | Long | Strong | Yes |
|  | ***4B*** | LOC100030953 (NCBI) |  |  |  |
| Transcript | XM_001380295 |  |  |  |  |
| Protein | XP_001380332 |  | Long | Strong | Yes |
|  | ***4C1*** | ENSMODG00000003263 |  |  |  |
| Transcript | ENSMODT00000004072 |  |  |  |  |
| Protein | ENSMODP00000003986 |  | Long | Weak | N.A. |
|  | ***4C2*** |  |  |  |  |
| Transcript | ENSMODT00000033910 |  |  |  |  |
| Protein | ENSMODP00000032331 |  | Long | Weak | N.A. |
|  | ***4D1*** | LOC100032166 (NCBI) |  |  |  |
| Transcript | ENSMODT00000024848 |  |  |  |  |
| Protein | ENSMODP00000024415 |  | Long | Strong |  |
|  | ***4D2*** |  |  |  |  |
| Transcript | ENSMODT00000024849 |  |  |  |  |
| Protein | ENSMODP00000024416 |  | Truncated Super-Short | Strong | Yes |
|  | ***4D3*** |  |  |  |  |
| Transcript | ENSMODT00000024850 |  |  |  |  |
| Protein | ENSMODP00000024417 |  | Super-Short | Strong | Yes |
|  | ***4D4*** |  |  |  |  |
| Transcript | ENSMODT00000024845 |  |  |  |  |
| Protein | ENSMODP00000024412 |  | Long | Strong | Yes |
|  | ***4D5*** |  |  |  |  |
| Transcript | XM_001381210 |  |  |  |  |
| Protein | XP_001381247 |  | Long | Strong |  |
| **Ecab** | ***4A1*** | LOC100147080 (NCBI) |  |  |  |
| Transcript | XR_044570  (miscellaneous mRNA) |  |  |  |  |
| Protein |  |  | Long | Strong | No |
|  | ***4B1*** | ENSECAG00000011003 |  |  |  |
| Transcript | XM_001500300 |  |  |  |  |
| Protein | XP_001500350 |  | Long | Strong | Yes |
|  |  |  |  |  |  |
|  |  |  |  |  |  |
| **Species** | ***Splice Variant*** | Gene Accession Number | Form Classification | EST Support | Multi-Genome Conservation |
| **Ecab** | ***4B2*** |  |  |  |  |
| Transcript | XM_001500306 |  |  |  |  |
| Protein | XP_001500356 |  | Long | Strong | Yes |
|  | ***4C*** | ENSECAG00000008059 |  |  |  |
| Transcript | ENSECAT00000008974 |  |  |  |  |
| Protein | ENSECAP00000006774 |  | Long | Strong | Yes |
|  | ***4D1*** | ENSECAG00000013771 |  |  |  |
| Transcript | XM_001494659 |  |  |  |  |
| Protein | XP_001494709 |  | Long | Strong | Yes |
|  | ***4D2*** |  |  |  |  |
| Transcript | XM_001494679 |  |  |  |  |
| Protein | XP_001494729 |  | Long | Strong | Yes |
|  | ***4D3*** |  |  |  |  |
| Transcript | XM_001494702 |  |  |  |  |
| Protein | XP_001494752 |  | Long | Strong | Yes |
|  | ***4D4*** |  |  |  |  |
| Transcript | XM_001494744 |  |  |  |  |
| Protein | XP_001494794 |  | Long | Strong | Yes |
|  | ***4D5*** |  |  |  |  |
| Transcript | ENSECAT00000014827 |  |  |  |  |
| Protein | ENSECAP00000011866 |  | Long | Strong | No |
| **Rnor** | ***4A1*** | ENSRNOG00000020828 |  |  |  |
| Transcript | L27062 |  |  |  |  |
| Protein | AAA56859 |  | Super-short | Strong | Yes |
|  | ***4A10*** |  |  |  |  |
| Transcript | ENSRNOT00000061099 |  |  |  |  |
| Protein | ENSRNOP00000057814 |  | Long | Strong | Yes |
|  | ***4A3*** |  |  |  |  |
| Transcript | CH473993 |  |  |  |  |
| Protein | EDL78317 |  | Long | Strong | No |
|  | ***4A4*** |  |  |  |  |
| Transcript | NM_013101 |  |  |  |  |
| Protein | NP_037233 |  | Long | Strong | Yes |
|  | ***4A5*** |  |  |  |  |
| Transcript | ENSRNOT00000041381 |  |  |  |  |
| Protein | ENSRNOP00000039312 |  | Dead-short | Strong | No |
|  | ***4B1*** | ENSRNOG00000005905 |  |  |  |
| Transcript | ENSRNOT00000007738 |  |  |  |  |
| Protein | ENSRNOP00000007738 |  | Long | Strong | Yes |
|  |  |  |  |  |  |
| **Species** | ***Splice Variant*** | Gene Accession Number | Form Classification | EST Support | Multi-Genome Conservation |
| **Rnor** | ***4B2*** |  |  |  |  |
| Transcript | ENSRNOT00000057676 |  |  |  |  |
| Protein | ENSRNOP00000054485 |  | Short | Strong | Yes |
|  | ***4B3*** |  |  |  |  |
| Transcript | NM_017031 |  |  |  |  |
| Protein | NP_058727 |  | Long | Strong | Yes |
|  | ***4B4*** |  |  |  |  |
| Transcript | AF202733 |  |  |  |  |
| Protein | AAL31764 |  | Long | Strong | No |
|  | ***4B5v1*** |  |  |  |  |
| Transcript | ENSRNOT00000057675 |  |  |  |  |
| Protein | ENSRNOT00000054484 |  | Super-Short | Strong | Yes |
|  | ***4B5v2*** |  |  |  |  |
| Transcript | ENSRNOT00000057674 |  |  |  |  |
| Protein | ENSRNOP00000054483 |  | Super-Short |  |  |
|  | ***4C1*** | ENSRNOG00000019518 |  |  |  |
| Transcript | XM_214325 |  |  |  |  |
| Protein | XP_214325 |  | Long | Strong | No |
|  | ***4C2*** |  |  |  |  |
| Transcript | ENSRNOT00000026457 |  |  |  |  |
| Protein | ENSRNOP00000026457 |  | Long | Strong | Yes |
|  | ***4C3*** |  |  |  |  |
| Transcript | NM_201607 |  |  |  |  |
| Protein | NP_963901 |  | Long | Strong | No |
|  | ***4D1*** | ENSRNOG00000042536 |  |  |  |
| Transcript | AH000839 |  |  |  |  |
| Protein | AAA18924 |  | Short | Strong | Yes |
|  | ***4D2*** |  |  |  |  |
| Transcript | NM_001113329 |  |  |  |  |
| Protein | NP_001106800 |  | Long | Strong | Yes |
|  | ***4D3*** |  |  |  |  |
| Transcript | NM_017032 |  |  |  |  |
| Protein | NP_001106803 |  | Long | Strong | Yes |
|  | ***4D4*** |  |  |  |  |
| Transcript | NM_001113328 |  |  |  |  |
| Protein | NP_001106799 |  | Long | Strong | Yes |
|  | ***4D5*** |  |  |  |  |
| Transcript | NM_001113332 |  |  |  |  |
| Protein | NP_058728 |  | Long | Strong | Yes |
|  |  |  |  |  |  |
| **Species** | ***Splice Variant*** | Gene Accession Number | Form Classification | EST Support | Multi-Genome Conservation |
| **Rnor** | ***4D7*** |  |  |  |  |
| Transcript | NM_001113334 |  |  |  |  |
| Protein | NP_001106805 |  | Long | Strong | Yes |
|  | ***4D9*** |  |  |  |  |
| Transcript | AY388961 |  |  |  |  |
| Protein | AAQ90405 |  | Long | Strong | Yes |
|  | ***4D*** |  |  |  |  |
| Transcript | CH473955 |  |  |  |  |
| Protein | EDM10320 |  | Long | Strong | No |
| **Mmus** | ***4A10*** | ENSMUSG00000032177 |  |  |  |
| Transcript | CH466522 |  |  |  |  |
| Protein | EDL25164 |  | Long | Strong | Yes |
|  | ***4A1*** |  |  |  |  |
| Transcript | NM_019798 |  |  |  |  |
| Protein | NP_062772 |  | Super-Short | Strong | Yes |
|  | ***4A5*** |  |  |  |  |
| Transcript | NM_183408 |  |  |  |  |
| Protein | NP_899668 |  | Long | Strong | Yes |
|  | ***4A6*** |  |  |  |  |
| Transcript | ENSMUST00000069577 |  |  |  |  |
| Protein | ENSMUSP00000066294 |  | Long | Strong | No |
|  | ***4B3*** | ENSMUSG00000028525 |  |  |  |
| Transcript | NM_019840 |  |  |  |  |
| Protein | NP_062814 |  | Long | Strong | Yes |
|  | ***4B5*** |  |  |  |  |
| Transcript | ENSMUST00000106901 |  |  |  |  |
| Protein | ENSMUSP00000102514 |  | Super-Short | Strong | Yes |
|  | ***4B5v2*** |  |  |  |  |
| Transcript | ENSMUST00000097949 |  |  |  |  |
| Protein | ENSMUSP00000095561 |  | Super-Short | Strong | No |
|  | ***4Bd*** |  |  |  |  |
| Transcript | ENSMUST00000106904 |  |  |  |  |
| Protein | ENSMUSP00000102517 |  | Long | Strong | No |
|  | ***4Be*** |  |  |  |  |
| Transcript | ENSMUST00000097950 |  |  |  |  |
| Protein | ENSMUSP00000095562 |  | Short | Strong | Yes |
|  | ***4B*** |  |  |  |  |
| Transcript | AK051102 |  |  |  |  |
| Protein | BAC34527 |  | Super-Short | Strong |  |
|  |  |  |  |  |  |
| **Species** | ***Splice Variant*** | Gene Accession Number | Form Classification | EST Support | Multi-Genome Conservation |
| **Mmus** | ***4Bg*** |  |  |  |  |
| Transcript | ENSMUST00000106911 |  |  |  |  |
| Protein | ENSMUSP00000102524 |  | Long | Strong | Yes |
|  | ***4C1*** | ENSMUSG00000031842 |  |  |  |
| Transcript | ENSMUST00000034307 |  |  |  |  |
| Protein | ENSMUSP00000034307 |  | Long | Strong | Yes |
|  | ***4D4*** | ENSMUSG00000021699 |  |  |  |
| Transcript | ENSMUST00000120671 |  |  |  |  |
| Protein | ENSMUSP00000112991 |  | Long | Strong | Yes |
|  | ***4Dc*** |  |  |  |  |
| Transcript | ENSMUST00000119507 |  |  |  |  |
| Protein | ENSMUSP00000114089 |  | Long | Strong | No |
|  | ***4D6*** |  |  |  |  |
| Transcript | ENSMUST00000117420 |  |  |  |  |
| Protein | ENSMUSP00000113610 |  | Super-Short | Strong | Yes |
|  | ***4D2v3*** |  |  |  |  |
| Transcript | ENSMUST00000119672 |  |  |  |  |
| Protein | ENSMUSP00000113567 |  | Truncated Super-Short | Strong | Yes |
|  | ***4D1*** |  |  |  |  |
| Transcript | ENSMUST00000120664 |  |  |  |  |
| Protein | ENSMUSP00000113024 |  | Short | Strong | Yes |
|  | ***4D10*** |  |  |  |  |
| Transcript | ENSMUST00000117879 |  |  |  |  |
| Protein | ENSMUSP00000112774 |  | Truncated Super-Short | Strong | No |
|  | ***4D9*** |  |  |  |  |
| Transcript | ENSMUST00000074103 |  |  |  |  |
| Protein | ENSMUSP00000073742 |  | Long | Strong | Yes |
|  | ***4D7*** |  |  |  |  |
| Transcript | NM_011056 |  |  |  |  |
| Protein | NP_035186 |  | Long | Strong | No |
|  | ***4D11*** |  |  |  |  |
| Transcript | EU489880 |  |  |  |  |
| Protein | ACA66114 |  | Long | Strong | No |
|  | ***4D*** |  |  |  |  |
| Transcript |  |  |  |  |  |
| Protein | CAQ51655 |  | Long | Strong | Yes |
|  |  |  |  |  |  |
|  |  |  |  |  |  |
| **Species** | ***Splice Variant*** | Gene Accession Number | Form Classification | EST Support | Multi-Genome Conservation |
| **Cjac** | ***4A1*** | ENSCJAG00000006698 |  |  |  |
| Transcript | ENSCJAT00000013111 |  |  |  |  |
| Protein | ENSCJAT00000012437 |  | Super-Short | Strong | Yes |
|  | ***4A2*** |  |  |  |  |
| Transcript | ENSCJAT00000013135 |  |  |  |  |
| Protein | ENSCJAP00000012461 |  | Long | Strong | Yes |
|  | ***4A3*** |  |  |  |  |
| Transcript | ENSCJAT00000013136 |  |  |  |  |
| Protein | ENSCJAP00000012462 |  | Long | Strong | No |
|  | ***4A4*** |  |  |  |  |
| Transcript | ENSCJAT00000013137 |  |  |  |  |
| Protein | ENSCJAP00000012463 |  | Long | Strong | Yes |
|  | ***4A5*** |  |  |  |  |
| Transcript | ENSCJAT00000013150 |  |  |  |  |
| Protein | ENSCJAP00000012475 |  | Long | Strong | Primate |
|  | ***4B1*** | ENSCJAG00000017022 |  |  |  |
| Transcript | ENSCJAT00000033140 |  |  |  |  |
| Protein | ENSCJAP00000031353 |  | Super-Short | Strong | Yes |
|  | ***4B2*** |  |  |  |  |
| Transcript | ENSCJAT00000033209 |  |  |  |  |
| Protein | ENSCJAP00000031417 |  | Long | Strong | Yes |
|  | ***4B3*** |  |  |  |  |
| Transcript | ENSCJAT00000033215 |  |  |  |  |
| Protein | ENSCJAP00000031423 |  | Long | Strong | Yes |
|  | ***4C1*** | ENSCJAG00000014122 |  |  |  |
| Transcript | ENSCJAT00000027433 |  |  |  |  |
| Protein | ENSCJAP00000025959 |  | Truncated Super-Short | Strong | Primate |
|  | ***4C2*** |  |  |  |  |
| Transcript | ENSCJAT00000027462 |  |  |  |  |
| Protein | ENSCJAP00000025987 |  | Long | Strong | Yes |
|  | ***4C3*** |  |  |  |  |
| Transcript | ENSCJAT00000027471 |  |  |  |  |
| Protein | ENSCJAP00000025994 |  | Long | Strong | Yes |
|  | ***4C4*** |  |  |  |  |
| Transcript | ENSCJAT00000027474 |  |  |  |  |
| Protein | ENSCJAP00000025996 |  | Long | Strong | Yes |
|  | ***4C5*** |  |  |  |  |
| Transcript | ENSCJAT00000027433 |  |  |  |  |
| Protein | ENSCJAP00000025959 |  | Truncated Super-Short | Strong | Primates |
| **Species** | ***Splice Variant*** | Gene Accession Number | Form Classification | EST Support | Multi-Genome Conservation |
| **Cjac** | ***4D1*** | ENSCJAG00000015215 |  |  |  |
| Transcript | ENSCJAT00000029722 |  |  |  |  |
| Protein | ENSCJAP00000028126 |  | Long | Strong | Yes |
|  | ***4D2*** |  |  |  |  |
| Transcript | ENSCJAT00000029727 |  |  |  |  |
| Protein | ENSCJAP00000028130 |  | Long | Strong | N.A. |
| ***Hsap*** | ***4A1*** | ENSG00000065989 |  |  |  |
| Transcript | NM_001111307 |  |  |  |  |
| Protein | NP_001104777 |  | Long | Strong | Yes |
|  | ***4A2*** |  |  |  |  |
| Transcript | NM_001111308 |  |  |  |  |
| Protein | NP_001104778 |  | Long | Strong | No |
|  | ***4A3/10*** |  |  |  |  |
| Transcript | NM_001111309 |  |  |  |  |
| Protein | NP_001104779 |  | Long | Strong | Yes |
|  | ***4A4*** |  |  |  |  |
| Transcript | NM_006202 |  |  |  |  |
| Protein | NP_006193 |  | Super-Short | Strong | Yes |
|  | ***4A8*** |  |  |  |  |
| Transcript | AY593872 |  |  |  |  |
| Protein | AAT00628 |  | Long | Strong | Primate |
|  | ***4B1*** | ENSG00000184588 |  |  |  |
| Transcript | NM_001037341 |  |  |  |  |
| Protein | NP_001032418 |  | Long | Strong | Yes |
|  | ***4B2*** |  |  |  |  |
| Transcript | NM_001037339 |  |  |  |  |
| Protein | NP_001032416 |  | Short | Strong | Yes |
|  | ***4B3*** |  |  |  |  |
| Transcript | NM_001037340 |  |  |  |  |
| Protein | NP_001032417 |  | Long | Strong | Yes |
|  | ***4B5*** |  |  |  |  |
| Transcript | EF595686 |  |  |  |  |
| Protein | ABQ85407 |  | Super-Short | Strong | Yes |
|  | ***4C1*** | ENSG00000105650 |  |  |  |
| Transcript | NM_000923 |  |  |  |  |
| Protein | NP_000914 |  | Long | Strong | Yes |
|  |  |  |  |  |  |
|  |  |  |  |  |  |
|  |  |  |  |  |  |
| **Species** | ***Splice Variant*** | Gene Accession Number | Form Classification | EST Support | Multi-Genome Conservation |
| **Hsap** | ***4C2*** |  |  |  |  |
| Transcript | NM_001098819 |  |  |  |  |
| Protein | NP_001092289 |  | Long | Strong | No |
|  | ***4C3*** |  |  |  |  |
| Transcript | NM_001098818 |  |  |  |  |
| Protein | NP_001092288 |  | Long | Strong | Yes |
|  | ***4C4*** |  |  |  |  |
| Transcript | ENST00000336173 |  |  |  |  |
| Protein | ENSP00000336624 |  | Truncated Super-Short | Strong | Primate |
|  | ***4D1v1*** | ENSG00000113448 |  |  |  |
| Transcript | HSU50157 |  |  |  |  |
| Protein | AAA97890 |  | Short | Strong | Yes |
|  | ***4D1v2*** |  |  |  |  |
| Transcript | NM_001104631 |  |  |  |  |
| Protein | NP_001098101 |  | Long | Strong | Yes |
|  | ***4D2v1*** |  |  |  |  |
| Transcript | AF012074 |  |  |  |  |
| Protein | AAC00070 |  | Truncated Super-Short | Strong | Yes |
|  | ***4D3*** |  |  |  |  |
| Transcript | NM_006203 |  |  |  |  |
| Protein | NP_006194 |  | Long | Strong | Yes |
|  | ***4D5*** |  |  |  |  |
| Transcript | AF012073 |  |  |  |  |
| Protein | AAC00069 |  | Long | Strong | Yes |
|  | ***4D6*** |  |  |  |  |
| Transcript | AF536975 |  |  |  |  |
| Protein | AAN10117 |  | Super-Short | Strong | Yes |
|  | ***4D7*** |  |  |  |  |
| Transcript | AF536976 |  |  |  |  |
| Protein | AAN10118 |  | Long | Strong | Yes |
|  | ***4D8*** |  |  |  |  |
| Transcript | AF536977 |  |  |  |  |
| Protein | AAN10119 |  | Long | Strong | Yes |
|  | ***4D9*** |  |  |  |  |
| Transcript | AY245867 |  |  |  |  |
| Protein | AAP75761 |  | Long | Strong | Yes |
