# Supplementary material for: An evolutionary analysis of cAMP-specific Phosphodiesterase 4 alternative splicing
Source: BMC Evol Biol. 2010 Aug 11;10:247. doi: 10.1186/1471-2148-10-247 (PMC2929239; doi:10.1186/1471-2148-10-247)
Supplement: Additional file 2 — Supplemental Figure S1. Protein Multiple Sequence Alignment Excluding the Amino Terminus From PDE4 gene isoforms. Supplemental Figure S1 is a clustalW alignment of the PDE4 proteins from all four gene isoforms. Highly conserved regions are the conserved region found in long forms (CRFL)-upstream conserved region 1 (UCR1) which contains a PKA phosphorylation motif from residues 36-39, linker region 1 (LR1), upstream conserved region 2 (UCR2) which contains the amino terminus for truncated super-short forms, linker region 2 (LR2), catalytic domain, which contains the metal-dependent phosphohydrolase motif from residues 356-609 and a Kinase Interaction Motif (KIM) from residues 484-497, and carboxy termini which contains an ERK phosphorylation site from residues 621-623 and "FQF" motif from 660-662. [file 1471-2148-10-247-S2.PPT]

## Slide 1
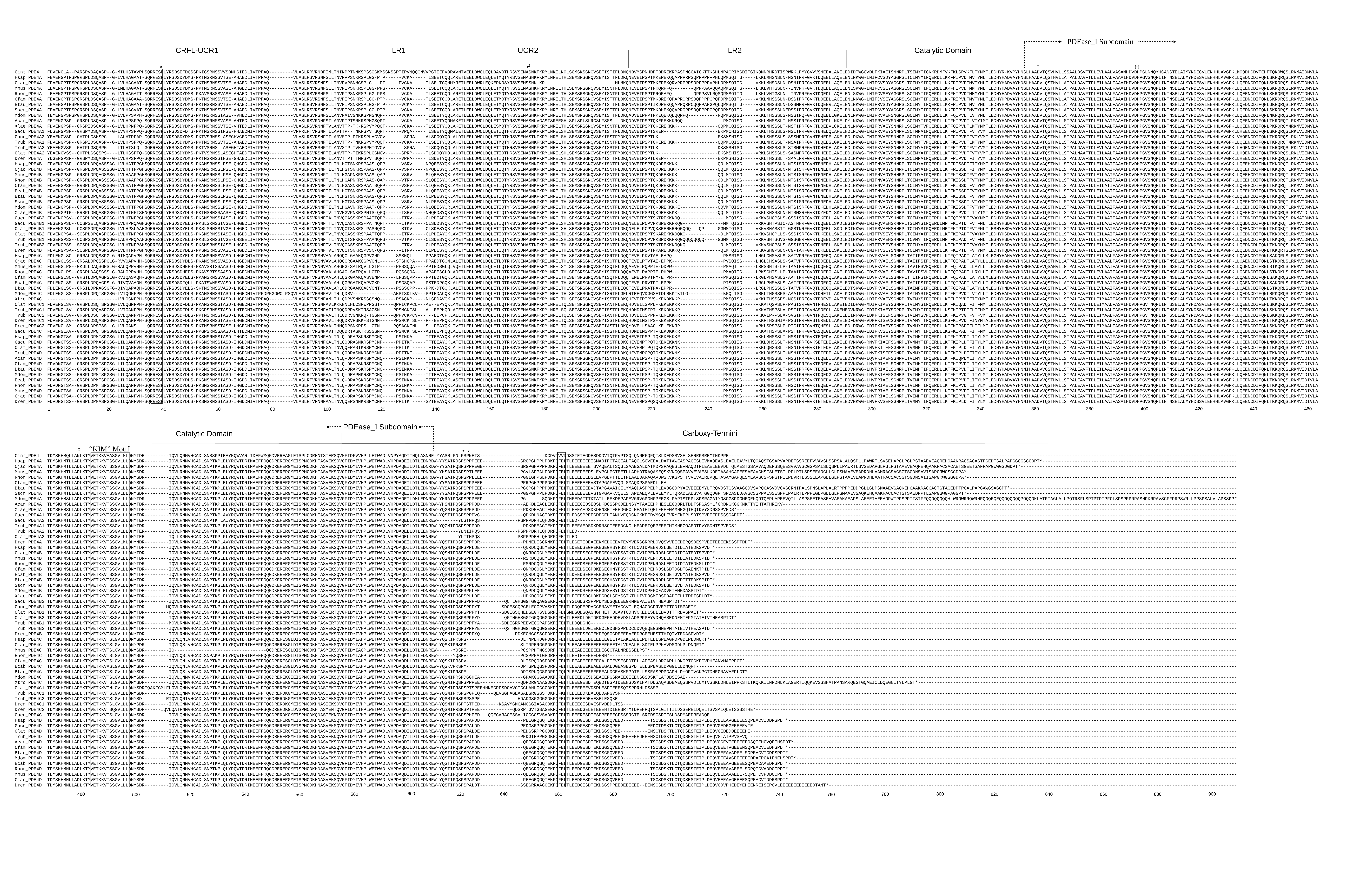

PDEase_I Subdomain
CRFL-UCR1
LR1
UCR2
LR2
Catalytic Domain
#
‡
Cint_PDE4 FDVENGLA--PARSPVDAQASP--G-MILHSTAVPHSQRRESFLYRSDSEFDQSSPKIGSRNSSVVSDMHGIEDLIVTPFAQ---------VLASLRRVRNDFIMLTNINPPTNNKSPSSQGKMSSNSSPTIPVNQQGNVVPGTEEFVQRAVNTVEELDWCLEQLDAVQTHRSVSEMASNKFKRMLNKELNQLSGMSKSGNQVSEFISTIFLDNQNDVMSPNHDPTDDREKRPASPNCGAIGKTTKSHLNPAGRIMGDITGIKQMNRHRDTISRWRKLPMYGVVVSNEEALAKELEDIDTWGGVDLFKIAEISNNRPLTSIMYTICKKRDMFVKFKLSPVKFLTYMMTLEDHYR-KVPYHNSLHAADVTQSVHVLLSSAALDSVFTDLEVLAALVASAMHDVDHPGLNNQYHCANSTELAIMYNDECVLENHHLAVGFKLMQQDHCDVFEHFTQKQWQSLRKMAIDMVLA
Hsap_PDE4A FEAENGPTPSPGRSPLDSQASP--G-LVLHAGAAT-SQRRESFLYRSDSDYDMS-PKTMSRNSSVTSE-AHAEDLIVTPFAQ---------VLASLRSVRSNFSLLTNVPVPSNKRSPLGG-PTP------VCKA-----TLSEETCQQLARETLEELDWCLEQLETMQTYRSVSEMASHKFKRMLNRELTHLSEMSRSGNQVSEYISTTFLDKQNEVEIPSPTMKEREKQQAPRPRPSQPPPPPVPHLQPMSQITG-----LKKLMHSNSLN-NSNIPRFGVKTDQEELLAQELENLNKWG-LNIFCVSDYAGGRSLTCIMYMIFQERDLLKKFRIPVDTMVTYMLTLEDHYHADVAYHNSLHAADVLQSTHVLLATPALDAVFTDLEILAALFAAAIHDVDHPGVSNQFLINTNSELALMYNDESVLENHHLAVGFKLLQEDNCDIFQNLSKRQRQSLRKMVIDMVLA
Cjac_PDE4A FDAENGPTPSPGRSPLDSQASP--G-LVLHAGAAT-SQRRESFLYRSDSDYDMS-PKTMSRNSSVTSE-AHAEDLIVTPFAQ---------VLASLRSVRSNFSLLTNVPVPSNNGSPLG---PT-----PVCKA-----TLSE-TCQHMYRETLEELDWRLEQKEPKQSYRSVSEMSSHK-KR--------------------------MLNKQNEVEIPSPTMKEREKQRVPRPRPSQPPPPPVPHLQPMSQITG-----VKKLMHSDSLN-DSNIPRFGVKTDQEELLAQELENLNKWG-LNIFCVSDYAGGRSLTCIMYMIFQERDLLKKFRIPVDTMVTYMLTLEDHYHADVAYHNSLHAADVLQSTHVLLATPALDAVFTDLEILAALFAAAIHDVDHPGVSNQFLINTNSELALMYNDESVLENHHLAVGFKLLQEDNCDIFQNLSKRQRQSLRKMVIDMVLA
Mmus_PDE4A LEAENGPTPSPGRSPLDSQASP--G-LMLHAGAAT-SQRRESFLYRSDSDYDMS-PKTMSRNSSVASE-AHGEDLIVTPFAQ---------VLASLRNVRSNFSLLTNVPIPSNKRSPLGG-PPS------VCKA-----TLSEETCQQLARETLEELDWCLEQLETMQTYRSVSEMASHKFKRMLNRELTHLSEMSRSGNQVSEYISNTFLDKQHEVEIPSPTPRQRPFQ--------QPPPAAVQQAQPMSQITG-----LKKLVHTGSLN--INVPRFGVKTDQEDLLAQELENLSKWG-LNIFCVSEYAGGRSLSCIMYTIFQERDLLKKFHIPVDTMMTYMLTLEDHYHADVAYHNSLHAADVLQSTHVLLATPALDAVFTDLEILAALFAAAIHDVDHPGVSNQFLINTNSELALMYNDESVLENHHLAVGFKLLQEENCDIFQNLSKRQRQSLRKMVIDMVLA
Rnor_PDE4A LEAENGPTPSPGRSPLDSQASP--G-LVLHAGATT-SQRRESFLYRSDSDYDMS-PKAVSRSSSVASE-AHAEDLIVTPFAQ---------VLASLRSVRSNFSLLTNVPIPSNKRSPLGG-PPS------VCKA-----TLSEETCQQLARETLEELDWCLEQLETMQTYRSVSEMASHKFKRMLNRELTHLSEMSRSGNQVSEYISNTFLDKQNEVEIPSPTPRQRAFQ--------QPPPSVLRQSQPMSQITG-----LKKLVHTGSLN--TNVPRFGVKTDQEDLLAQELENLSKWG-LNIFCVSEYAGGRSLSCIMYTIFQERDLLKKFHIPVDTMMMYMLTLEDHYHADVAYHNSLHAADVLQSTHVLLATPALDAVFTDLEILAALFAAAIHDVDHPGVSNQFLINTNSELALMYNDESVLENHHLAVGFKLLQEENCDIFQNLSKRQRQSLRKMVIDMVLA
Cfam_PDE4A FEAENGPTPSPGRSPLDSQASP--G-LVLHAGAAA-SQRRESFLYRSDSDYDMS-PKTMSRNSSVTSE-AHAEDLIVTPFAQ---------VLASLRSVRSNFSLLTNVPIPSNKRSPLGG-PTP------VCKA-----TLSEETCQQLARETLEELDWCLEQLETMQTYRSVSEMASHKFKRMLNRELTHLSEMSRSGNQVSEYISTTFLDKQNEVEIPSPTMKDREKQPAPRQRPSQQPPPPGPQFQPMSQITG-----VKKLMHSSSLN-DSSIPRFGVKTDQEELLAQELENLNKWG-LNIFCVSEYAGGRSLSCIMYTIFQERDLLKKFRIPVDTMVTYMLTLEDHYHPDVAYHNSLHAADVLQSTHVLLATPALDAVFTDLEILAALFAAAIHDVDHPGVSNQFLINTNSELALMYNDESVLENHHLAVGFKLLQEDNCDIFQNLSKRQRQSLRKMVIDMVLA
Btau_PDE4A LEAENGPTPSPGRSPLDSQASP--G-LVLHAGAAT-SQRRESFLYRSDSDYDMS-PKTMSRNSSVTSE-AHAEDLIVTPFAQ---------VLASLRSVRSNFSLLTNVPIPSNKRSPLGG-PTP------VCKA-----TLSEETCQQLARETLEELDWCLEQLETMQTYRSVSEMASHKFKRMLNRELTHLSEMSRSGNQVSEYISTTFLDKRNEVEIPSPTIKDREKQQAPRQRPCQQPPAPGPQLQPMSQITG-----VKKLMHSSSLN-DSSMPRFGVKTDQEERLAQELENLNKWG-LNIFRVSDYAGGRSLSCIMYTIFQERDLLKKFRIPVDTMVTYMLTLEDHYHPDVAYHNSLHAADVLQSTHVLLATPALDAVFTDLEILAALFAAAIHDVDHPGVSNQFLINTNSELALMYNDESVLENHHLAVGFKLLQEDNCDIFQNLGKRQRQSLRKMVIDMVLA
Sscr_PDE4A FEAENGPTPSPGRSPLDSQASP--G-LVLHAGVAT-SQRRESFLYRSDSDYDMS-PKTMSRNSSVTSE-AHAEDLIVTPFAQ---------VLASLRSVRSNFSLLTNVPIPSNKRSPLGG-PTP------VCKA-----TLSEETCQQLARETLEELDWCLEQLETMQTYRSVSEMASHKFKRMLNRELTHLSEMSRSGNQVSEYISTTFLDKQNEVDIPSPTMKDHEKQQAPRQRPSQQPPPPGPQFQPMSQITG-----VKKLMHSSSLNEDSSIPRFGVKTDQEELLAQELENLNKWG-LNIFCVSDYAGGRSLSCIMYTIFQERDLLKKFRIPVDTMVTYMLTLEDHYHPDVAYHNSLHAADVLQSTHVLLATPALDAVFTDLEILAALFAAAIHDVDHPGVSNQFLINTNSELALMYNDESVLENHHLAVGFKLLQEDNCDIFQNLSKRQRQSLRKMVIDMVLA
Mdom_PDE4A IEMENGPSPSPGRSPLDSQASP--G-LVLPPSAPH-SQRRESFLYRSDSDYDMS-PKTMSRNSSIASE--VHEDLIVTPFAQ---------VLASLRSVRSNFSLLANVPAIVGNKKSPMGNQP------AVCKA-----TLSEETYQQLARETLEELDWCLEQLETMQTYRSVSEMASHKFKRMLNRELTHLSEMSRSGNQVSEYISTTFLDKQADVEIPPPTPKEQEKQLQQRPQ-----------RQPMSQISG-----VKKLTHSSSLS-NSGIPQFGVKTDQEELLGKELENLNKWG-LNIFRVAEFSNGRSLGCIMYTIFQERDLLKTFQIPVDTLVTYMLTLEDHYHADVAYHNSLHAADVLQSTHVLLATPALDAVFTDLEILAALFAAAIHDVDHPGVSNQFLINTNSELALMYNDESVLENHHLAVGFKLLQEENCDIFQNLNKRQRQSLRRMVIDMVLA
Acar_PDE4A FEIENGPSP--GRSPLDSQASP--G-LVLHPSFPQ-SQRRESFLYRSDSDYDMS-PKTMSRNSSVASE-AHTEDLIVTPFAQ---------VLASLRSVRNNFSILANVPTPTSNKRSPMGSQPT------VCKA-----TLSEETYQQMAKETLEELDWCLDQLETIQTYRSVSEMASNKVGASISREESHLSPLSPLSLRCSLFSSS---DKQNDVEIPSPTQKEREKKKRQQ---------------PMCQISG-----VKKLMHSSSLT-NSSIPRFGVKTDQEDLLNKELDYLNKWG-LNIFRVAEYSNNRSLSCIMYTIFQERDLLKTFKIPVDTLVTYIMTLEDHYHADVAYHNSLHAADVTQSTHVLLSTPALDAVFTDLEILAAIFAAAIHDVDHPGVSNQFLINTNSELALMYNDESVLENHHLAVGFKLLQEENCDIFQNLTKRQRQTLRKMVIDMVLA
Xlae_PDE4A FDVENGPSP--GRSPIDSQASP--G-LVLHPNFPQ-SQRRESFLYRSDSDYDMS-PKTMSRNSSVTSE-VHTEDLIVTPFAQ---------VLASLRSVRNNFTVLANVTTP-TK-RSPVMPQQT------VCKA-----TLSEETYQQLAKETLEELDWCLDQLESMQTYRSVSEMASNKFKRMLNRELTHLSEMSRSGNQVSEYISNTFLDKQNEVEIPSPTQKEREKKKK---------------QQPMCQISG-----VKKLMHSSSLT-NSTIPRFGVKTDQEEVLCKELDNLNKWG-LNIFRVAEYSNNRPLSCIMYTVFQERELLKTFQIPVDTLMTYMMTLEDHYHADVAYHNSLHAADVTQSTHVLLSTPALDAVFTDLEILAALFAAAIHDVDHPGVSNQFLINTNSELALMYNDESVLENHHLAVGFKLLQEENCDIFQNLPKRQRQMMRKMVIDMVLA
Gacu_PDE4A1 FDSENGPSP--GRSPMDSQASP--G-LVVHPSFPQ-SQRRESFLYRSDSDFDTS-PKTMSRNSSINSE-RHAEDMIVTPFAQ---------VRFRLRTVRSNFTILAVTTP--TNKRSPVTSQPT------VPQA-----TLSEETYQQMALETLEELDWCLDQLETIQTHRSVSEMASNKFKRMLNRELSHLSEMSRSGNQVSEYISTTFLDKQNEVEIPSPTSRER--------------------EKPMCHISG-----VKKLTHSSSLS-NSITPRFGVKTEHEDQLARELNDLNIWG-LNIFRVAEYSNNRPLSCTMFAIFQERDLLKTFRIPVDTFVTYVMTLEDHYHANVAYHNSLHAADVTQSTHVLLSTPALDAVFTDLEILAALFAAAIHDVDHPGVSNQFLINTNSELALMYNDESVLENHHLAVGFKLLHEENCDIFQNLSKRQRQSLRKLVIDMVLA
Gacu_PDE4A2 YEAENGVSP--GHTPLGSHSPG----LALHTPFAP-GQRRESFLYRSDSDYDMS-PKTVSRNSSLASEGHVGEDFIVTPFAQ---------VLASLRSVRSNFTILANVSTP-PIKRSPLAGVCV-------SPRA----ALSDQQYQQLALDTLEELDWCLDQLETIQTHRSVSEMASTKFKRMLNRELSHLSEMSRSGNQVSEYISSTFMDKQNDVEIPSPTLK----------------------EKSMSHISG-----VRKLSHSSSLS-SSSMPRFGVNTEHEDELAKELEDLDKWS-FNIFRVAEFSNNRPLSCIMYTIFQERELLKTFRIPVETFVTYVMTLEDHYHENIPYHNSLHAADVAQSTHVLLSTPALDAVFTDLEILAALFAAAIHDVDHPGVSNQFLINTNSELALMYNDESVLENHHLAVGFKLVHQENCDIFQNLTKRQRQSLRKLVIDMVLA
Trub_PDE4A1 FDVENGPSP--GRSPIDSQASP--G-LVLHPSFPQ-SQRRESFLYRSDSDYDMS-PKTMSRNSSVTSE-AHAEDLIVTPFAQ---------VLASLRSVRNNFTILANVTTP-TNKRSPVMPQQT------VCKA-----TLSEETYQQLAKETLEELDWCLDQLETMQTYRSVSEMASNKFKRMLNRELTHLSEMSRSGNQVSEYISNTFLDKQNEVDIPSPTQKEREKKKK---------------QQPMCQISG-----VKKLMHSSSLT-NSAIPRFGVKTDQEESLGKELDNLNKWG-LNIFRVAEYSNNRPLSCTMYTVFQERELLKTFKIPVDTLMTYMMTLEDHYHADVAYHNSLHAADVTQSTHVLLSTPALDAVFTDLEILAALFAAAIHDVDHPGVSNQFLINTNSELALMYNDESVLENHHLAVGFKLLQEENCDIFQNLTKRQRQTMRKMVIDMVLA
Trub_PDE4A2 YEAENGVSP--GHTPLGSQSPG----LTLHTSLQ--GQRRESFLYRSDSDYDMS-PKTVSRNS-LASEGHTAEDFIVTPFAQ---------VLASLRSVRSNFTILANVSTP-TVKRSPMTGVCV-------SPRA----TLSDQQYQQLALDTLEELDWCLDQLETIQTHRSVSEMASNKFKRMLNRELSHLSEMSRSGNQVSEYISSTFLDKQNEVEIPSPTLK----------------------DKSMSHISG-----VRKLSHSSSLS-STSMPRFGVNTDHEDELAKELEDLDKWS-FNIFKVAEFSNNRPLSCIMYAIFQERELLKTFRIPVDTFVTYVMTLEDHYHSNVAYHNSLHAADVTQSTHVLLSTPALDAVFTDLEVLAALFAAAIHDVDHPGVSNQFLINTNSELALMYNDESVLENHHLAVGFKLLHQENCDIFQNLTKRQRQSLRKLVIDIVLA
Olat_PDE4A2 YEAENGVSS--GHTPLGSQSPS----LTLHSSFTQ-GQRRESFLYRSDSDYDMS-PKTVSRNSSLASEGHTAEDFIVTPFAQ---------VLASLRSVRSNFTILANVTTP-TIKRSPLGGMCV------SPRP-----TLSDQQYHQLALDTLEELDWCLDQLETIQTHRSVSEMASTKFKRMLNRELSHLSEMSRSGNQVSEYISSTFMDKQNEVEIPSPTLK----------------------EKSMSHISG-----VRKLSHSSSLS-SASMPRFGVNTDHEDELAKELEDLDKWS-FNVFKVAEYSNNRPLSCIMYAIFQERELLKTFRIPVDTFVTYVMTLEDHYHGNVAYHNSLHAADVTQSTHVLLSTPALNAAFTDLEILAALFAAAIHDVDHPGVSNQFLINTNSELALMYNDESVLENHHLAVGFKLLHQENCDIFQNLTKRQRQSLRKLVIEMVLA
Drer_PDE4A YDGENGPSP--GRSPMDSQASP--G-LVLHPSFPQ-SQRRESFLYRSDSDYDMS-PKTMSRNSSINSE-GHAEDLIVTPFAQ---------VLASLRTVRSNFTILANVTTPTTTMRSPVTSQPT------VPPA-----TLSDETYQQLARETLEELDWCLDQLETIQTHRSVSEMASNKFKRMLNRELSHLSEMSRSGNQVSEYISTTFLDKQNEVEIPSPTLRER--------------------EKPMSHISG-----VKKLTHSSSLT-SAALPRFGVKTEQEDALARELNDLNKWG-LNIFHVAEFSNNRPLSCIMFAIFQERDLLKTFRIPVDTFITYVMTLEDHYHANVAYHNSLHAADVTQSTHVLLSTPALDAVFSDLEILAALFAAAIHDVDHPGVSNQFLINTNSELALMYNDESVLENHHLAVGFKLLHEENCDIFQNLTKRQRQSLRKLVIDMVLA
Hsap_PDE4B FDVENGPSP--GRSPLDPQASSSAG-LVLHATFPGHSQRRESFLYRSDSDYDLS-PKAMSRNSSLPSE-QHGDDLIVTPFAQ---------VLASLRSVRNNFTILTNLHGTSNKRSPAAS-QPP------VSRV-----NPQEESYQKLAMETLEELDWCLDQLETIQTYRSVSEMASNKFKRMLNRELTHLSEMSRSGNQVSEYISNTFLDKQNDVEIPSPTQKDREKKKK---------------QQLMTQISG-----VKKLMHSSSLN-NTSISRFGVNTENEDHLAKELEDLNKWG-LNIFNVAGYSHNRPLTCIMYAIFQERDLLKTFRISSDTFITYMMTLEDHYHSDVAYHNSLHAADVAQSTHVLLSTPALDAVFTDLEILAAIFAAAIHDVDHPGVSNQFLINTNSELALMYNDESVLENHHLAVGFKLLQEEHCDIFMNLTKKQRQTLRKMVIDMVLA
Cjac_PDE4B FDVENGPSP--GRSPLDPQASSSSG-LVLHTTFPGHSQRRESFLYRSDSDYDLS-PKAMSRNSSLPSE-QHGDDLIVTPFAQ---------VLASLRSVRNNFTILTNLHGTSNKRSPAAS-QPP------VSRV-----NPQEESYQKLAMETLEELDWCLDQLETIQTYRSVSEMASNKFKRMLNRELTHLSEMSRSGNQVSEYISNTFLDKQNDVEIPSPTQKDREKKKK---------------QQLMTQISG-----VKKLMHSSSLN-NTSISRFGVNTENEDHLAKELEDLNKWG-LNIFNVAGYSHNRPLTCIMYAIFQERDLLKTFRISSDTFITYMMTLEDHYHSDVAYHNSLHAADVAQSTHVLLSTPALDAVFTDLEILAAIFAAAIHDVDHPGVSNQFLINTNSELALMYNDESVLENHHLAVGFKLLQEEHCDIFMNLTKKQRQTLRKMVIDMVLA
Mmus_PDE4B FDVENGPSP--GRSPLDPQAGSSSG-LVLHAAFPGHSQRRESFLYRSDSDYDLS-PKAMSRNSSLPSE-QHGDDLIVTPFAQ---------VLASLRSVRNNFTLLTNLHGAPNKRSPAAS-QAP------VSRV-----SLQEESYQKLAMETLEELDWCLDQLETIQTYRSVSEMASNKFKRMLNRELTHLSEMSRSGNQVSEYISNTFLDKQNDVEIPSPTQKDREKKKK---------------QQLMTQISG-----VKKLMHSSSLN-NTSISRFGVNTENEDHLAKELEDLNKWG-LNIFNVAGYSHNRPLTCIMYAIFQERDLLKTFKISSDTFVTYMMTLEDHYHSDVAYHNSLHAADVAQSTHVLLSTPALDAVFTDLEILAAIFAAAIHDVDHPGVSNQFLINTNSELALMYNDESVLENHHLAVGFKLLQEEHCDIFQNLTKKQRQTLRKMVIDMVLA
Rnor_PDE4B FDVENGPSP--GRSPLDPQASSSSG-LVLHAAFPGHSQRRESFLYRSDSDYDLS-PKAMSRNSSLPSE-QHGDDLIVTPFAQ---------VLASLRIVRNNFTLLTNLHGAPNKRSPAAS-QAP------VTRV-----SLQEESYQKLAMETLEELDWCLDQLETIQTYRSVSEMASNKFKRMLNRELTHLSEMSRSGNQVSEYISNTFLDKQNDVEIPSPTQKDREKKKK---------------QQLMTQISG-----VKKLMHSSSLN-NTSISRFGVNTENEDHLAKELEDLNKWG-LNIFNVAGYSHNRPLTCIMYAIFQERDLLKTFKISSDTFVTYMMTLEDHYHSDVAYHNSLHAADVAQSTHVLLSTPALDAVFTDLEILAAIFAAAIHDVDHPGVSNQFLINTNSELALMYNDESVLENHHLAVGFKLLQEEHCDIFQNLTKKQRQTLRKMVIDMVLA
Cfam_PDE4B FDVENGPSP--GRSPLDPQASSSSG-LVLHATFPGHSQRRESFLYRSDSDYDLS-PKAMSRNSSLPSE-QHGDDLIVTPFAQ---------VLASLRSVRNNFTVLTNLHGASNKRSPAATSQPP------VSRV-----NLQEESYQKLAMETLEELDWCLDQLETIQTYRSVSEMASNKFKRMLNRELTHLSEMSRSGNQVSEYISNTFLDKQNDVEIPSPTQKDREKKKK---------------QQLMTQISG-----VKKLMHSSSLN-NTSISRFGVNTENEDHLAKELEDLNKWG-LNIFNVAGYSHNRPLTCIMYAIFQERDLLKTFKISSDTFVTYMMTLEDHYHSDVAYHNSLHAADVAQSTHVLLSTPALDAVFTDLEILATIFAAAIHDVDHPGVSNQFLINTNSELALMYNDESVLENHHLAVGFKLLQEEHCDIFQNLTKKQRQTLRKMVIDMVLA
Ecab_PDE4B FDVENGPSP--GRSPLDPQASSSSG-LVLHATFPGHSQRRESFLYRSDSDYDLS-PKAMSRNSSLPSE-QHGDDLIVTPFAQ---------VLASLRSVRNNFTILTNLHGTSNKRSPAAS-QPP------VSRV-----HLQEESYQKLAMETLEELDWCLDQLETIQTYRSVSEMASNKFKRMLNRELTHLSEMSRSGNQVSEYISNTFLDKQNDVEIPSPTQKDREKKKK---------------QQLMTQISG-----VKKLMHSSSLN-NTSISRFGVNTENEDHLAKELEDLNKWG-LNIFNVAGYSHNRPLTCIMYAIFQERDLLKTFKISSDTFVTYMMTLEDHYHSDVAYHNSLHAADVAQSTHVLLSTPALDAVFTDLEILAAIFAAAIHDVDHPGVSNQFLINTNSELALMYNDESVLENHHLAVGFKLLQEEHCDIFQNLTKKQRQTLRKMVIDMVLA
Btau_PDE4B FDVENGPSP--GRSPLDPQASSSSG-LVLHATFPGHSQRRESFLYRSDSDYDLS-PKAMSRNSSLPSE-QHGDDLIVTPFAQ---------VLASLRSVRNNFTLLTNLHGTSNKRSPAAG-QPS------VSRV-----NLPEESYQKLAMETLEELDWCLDQLETIQTYRSVSEMASNKFKRMLNRELTHLSEMSRSGNQVSEYISNTFLDKQNDVEIPSPTQKDREKKKK---------------QQLMTQISG-----VKKLMHSSSLN-NTSISRFGVNTENEDHLAKELEDLNKWG-LNIFNVAGYSHNRPLTCIMYAIFQERDLLKTFKISSDTFVTYMMTLEDHYHADVAYHNSLHAADVAQSTHVLLSTPALDAVFTDLEILAAIFAAAIHDVDHPGVSNQFLINTNSELALMYNDESVLENHHLAVGFKLLQEEHCDIFQNLTKKQRQTLRKMVIDMVLA
Sscr_PDE4B FDVENGPSP--GRSPLDPQASSSSG-LVLHATFPGHSQRRESFLYRSDSDYDLS-PKAMSRNSSLPSE-QHGDDLIVTPFAQ---------VLASLRSVRNNFTVLTNLHGTSNKRSPAAS-QPP------VSRV-----NLPEESYQKLAMETLEELDWCLDQLETIQTYRSVSEMASNKFKRMLNRELTHLSEMSRSGNQVSEYISNTFLDKQNDVEIPSPTQKDREKKKK---------------QQLMTQISG-----VKKLMHSSSLN-NTSISRFGVNTENEDHLAKELEDLNKWG-LNIFNVAGYSHNRPLTCIMYAIFQERDLLKTFKISSDTLVTYMMTFEDHYHSDVAYHNSLHAADVAQSTHVLLSTPALDAVFTDLEILAAIFAAAIHDVDHPGVSNQFLINTNSELALMYNDESVLENHHLAVGFKLLQEEHCDIFQNLTKKQRQTLRKMVIDMVLA
Mdom_PDE4B FDVENGPSP--GRSPLDPQAGSSSG-LVLHTTFPGHSQRRESFLYRSDSDYDLS-PKAMSRNSSLPSE-QHGDDLIVTPFAQ---------VLASLRSVRNNFTILTNLHGAVNKRSPAAT-QPP------VSRV-----NLQEESYQRLAMETLEELDWCLDQLETIQTYRSVSEMASNKFKRMLNRELTHLSEMSRSGNQVSEYISNTFLDKQNEVEIPSPTQKDREKKKKE--------------QQVMTQISG-----VKKLMHSSSLN-NTSISRFGVKTEKEDHLAKELEDLNKWG-LNIFKVAGYSHNRPLTCIMYAIFQERDLLKTFKISSDTFVTYMMTLEDHYHSDVAYHNSLHAADVAQSTHVLLSTPALDAVFTDLEILAAIFAAAIHDVDHPGVSNQFLINTNSELALMYNDESVLENHHLAVGFKLLQEEHCDIFQNLTKKQRQTLRKMVIDMVLA
Xlae_PDE4B FDVENGPTP--GRSPLDHQASPGSG-LVLHTNFTSHNQRRESFLYRSDSDYDLS-PKTMSRNSSAASE-QHGDDLIVTPFAQ---------VLASLRSVRNNFTVLTNVHGVPNKRSPMTS-QPQ------ISRV-----NHQEDSYQKIAMDTLEELDWCLDQLETIQTYRSVSEMASNKFKRMLNRELTHLSEMSRSGNQVSEYISSTFLDKQNDVEIPSPTQKDREKKKK---------------QQLMTQISG-----VKKLKHSSSLN-NTSMSRFGVKTDYEDMLSKELEDLNKWG-LNIFKVASYSCNRPLTCIMYAIFQERDLLKTFKIPVDTLITYTMTLEDHYHSDVAYHNSLHAADVTQSTHVLLSTPALDAVFTDLEILAAIFAAAIHDVDHPGVSNQFLINTNSELALMYNDESVLENHHLAVGFKLLQEEHCDIFQNLTKKQRQSLRKMVIDLVLA
Gacu_PDE4B2 FDVENGPSV--GCSPLDPQASPGSG-LVLHTNFPGHNQRRESFLYRSDSDYDLS-PKSMSRNSSIASE-LHGDDLIVTPFAQ---------VLASLRSVRNNFTALTNVQCASSKRSPAATTQPP------ITRV-----CLPDEAFQKLAMETMEELDWCLDQLETIQTYRSVSDMASNKFKRMLNRELTHLSEMSRSGNQVSEFISNTFLDKQNEVEIPSPTSKTREKKKQQ----------------LMTQISG-----VKKVSHGPSLS-GSSISRFGVKTDKEELLAKELEDLNKWG-LNIFTVSEYSHNRPLTCIMYAIFQERDLLKTFQIPVDTFVAYMMTLEDHYHSDVAYHNSLHAADVAQSTHILLSTPALDAVFTDLEILAAIFAAAIHDVDHPGVSNQFLINTNSELALMYNDESVLENHHLAVGFKLLQEDNCDIFQNLTKKQRQSLRKMAIDMVLA
Gacu_PDE4B1 FEGENGPSL--CCSPSELQASPGSG-LVLHPNQAGHGQRRESFLYRSDSDYELS-PKSLSRNSSIVGE-LHGEDLIVTPFAQ---------VLASLRTVRNNFTTLTNVQCASNKRS-PATNQPT------LTKV-----CLSDDSYQKLAMETMEELDWCLDQLETIQTYRSVSDMASNKFKRMLNRELSHLSEMSRSGNQVSEYISNTFLEKQNELELPCPVPKSRERKRRQG---------------MMTQISG-----VRKVSHTPSIC-ASTNNRFGVKTDQEELLSKDLEGINKWG-LNIFQVAEHSRNRPLTSIMYSIFQERDLMRTFKIPTDTFVTFMLTLEDHYHSDVAYHNSLHAADVAQSTHILLSTPALDEVFTDLEILAAIFAAAIHDVDHPGVSNQFLINTNSELALMYNDESVLENHHLAVGFKLLQGDNCDIFQNLTKKQKQTLRRMVIDMVLA
Olat_PDE4B1 FEVENGPSL--CCSPSDPQASPGSG-LVLHPSLAAHGQRRESFLYRSDSDYELS-PKSLSRNSSIVGE-LHGEDLIVTPFAQ---------VLASLRTVRNNFTTLTNVQCTSNKRS-PASNQPC------STKV-----CLSDESYQKLAMETMEELDWCLDQLETIQTYRSVSDMASNKFKRMLNRELSHLSEMSRSGNQVSEYISNTFLDKQNELELPCPVQKSRERKRRQGQQQ---QP-----GGMMTQISG-----VKKVSNASSIT-GGSTNRFGVKTDQEELLSKDLEDINKWG-LNIFRVAEHSHNRPLTCIMYSIFQERDLMRTFKIPTDTFVTFMLTLESHYHSDVAYHNSLHAADVAQSTHILLSTPALDAVFTDLEILAAIFAAAIHDVDHPGVSNQFLINTNSELALMYNDESVLENHHLAVGFKLLQEENCDIFQNLTKKQKQTLRRMVIDMVLA
Olat_PDE4B2 FDVENGPSA--SCSPLDPQASPGSG-LVLHTNFPGHNQRRESFLYRSDSDYELS-PKSMSRNSSIASE-LHGDDLIVTPFAQ---------VLASLRSVRNNFTVLTNVQCASSKRSPAATTQPP------ITRV-----CLPDEAYQKLAVETMEELDWCLDQLETIQTYRSVSDMASNKFKRMLNRELTHLSEMSRSGNQVSEFISNTFLDKQNDVEIPSPTSKAREKKKQQKQ-------------QLMTQISG-----VKKVSHGPLLS-SSSISRFGVKTDKEELLSKELEELNKWG-LNIFTVSEYSNNRPLTCIMYAIFQERDLLKTFKIPADTFVAYMMTLEDHYHSDVAYHNSLHAADVAQSTHILLSTPALDAVFTDLEILAAIFAAAIHDVDHPGVSNQFLINTNSELALMYNDESVLENHHLAVGFKLLQEDNCDIFQNLTKKQRQSLRKMVIDMVLA
Trub_PDE4B1 FEGENGPSS--CCSPSDPQASPGSG-LALHPNQAAHGQRRESFLYRSDSDYELS-PKSLSRNSSIVGE-LHSEELIVTPFAQ---------VLASLRIVRNNFTTLTNVQCTSFKKS-PAANQPS------VTKV-----CLSDESYQKLAMETMEELDWCLDQLETIQTYRSVSDMASNKFKRMLNRELSHLSEMSRSGNQVSEYISNTFLDKQNELEVPCPVPKSRDRKRRQGQQQQQQQQ-----GGMMTQISG-----VRKVSHTSGVS-GGSGNRFGVKTDQEELLSKDLEDINKWG-LNIFRVAEHSHNRPLTCVMYTIFQERDLMRTFKIPADTFVTFMLTLESHYHSDVAYHNSLHAADVAQSTHILLSTPALDAVFTDLEILAAIFAAAIHDVDHPGVSNQFLINTNSELALMYNDESVLENHHLAVGFKLLQEDNCDIFQNLTKKQRQTLRRMVIDMVLA
Trub_PDE4B2 FDVENGPSS--SCSPLDPQASPGSG-LVLHTNFPSHSQRRESFLYRSDSDYDLS-PKSMSRNSSIASE-LHGDDLIVTPFAQ---------VLASLRSVRNNFTVLTNVQCASSKRSPAATTQPP------FTRV-----CLPDEAYQKLAMETMEELDWCLDQLETIQTYRSVSDMASTKFKRMLNRELTHLSEMSRSGNQVSEFISNTFLDKQNEVEIPSPTSKTREKKKQQKQ-------------QLMTQISG-----VKKVSHGPSLS-SSSISRFGVKTDNEELLSKELENLNKWG-LNIFTVSEYSHNRPLTCIMYAIFQERDLLKTFKIPVDTFVAYMMTLEDHYHSDVAYHNSLHAADVAQSTHILLSTPALDAVFTDLEILAAIFAAAIHDVDHPGVSNQFLINTNSELALMYNDESVLENHHLAVGFKLLQEDNCDLFQNLTKKQRQSLRKMVIDMVLA
Drer_PDE4B FDVENGPSA--SCSPLDPQASPGSG-LVLHTNFPGHNQRRESFLYRSDSDYDLS-PKSMSRNSSVASE-LHGDDLIVTPFAQ---------VLASLRSVRNNFTVLTNVQCTSNKRSPVSN-QPS------ITRV-----CLPDESYQKLAVETMEELDWCLDQLETIQTYRSVSDMASNKFKRMLNRELTHLSEMSRSGNQVSEFISNTFLDKQNDVEIPSPTPKAREKKKKQ---------------QLMTQISG-----VKKVSHGPSLN-CG-IARFGVKTDKEDLLSKELEDLNRWG-LNIFTVSEYSNNRPLTCIMYAIFQERDLLKTFKIPTDTFVTYMMTLEDHYHQDVAYHNSLHAADVAQSTHILLSTPALDAVFTDLEILAAIFAAAIHDVDHPGVSNQFLINTNSELALMYNDESVLENHHLAVGFKLLQEDNCDIFQNLTKKQRTSLRRMVIDMVLA
Hsap_PDE4C FDLENGLSC--GRRALDPQSSPGLG-RIMQAPVPH-SQRRESFLYRSDSDYELS-PKAMSRNSSVASD-LHGEDMIVTPFAQ---------VLASLRTVRSNVAALARQQCLGAAKQGPVGNP------SSSNQL-----PPAEDTGQKLALETLDELDWCLDQLETLQTRHSVGEMASNKFKRILNRELTHLSETSRSGNQVSEYISRTFLDQQTEVELPKVTAE-EAPQ-------------------PMSRISG-----LHGLCHSASLS-SATVPRFGVQTDQEEQLAKELEDTNKWG-LDVFKVAELSGNRPLTAIIFSIFQERDLLKTFQIPADTLATYLLMLEGHYHANVAYHNSLHAADVAQSTHVLLATPALEAVFTDLEILAALFASAIHDVDHPGVSNQFLINTNSELALMYNDASVLENHHLAVGFKLLQAENCDIFQNLSAKQRLSLRRMVIDMVLA
Cjac_PDE4C FDLENGLSS--GRSALDPQSSPGLG-RVVQAPVHH-SQRRESFLYRSDSDYDLS-PKAMSRNSSVASD-LHGEDMIVTPFAQ---------VLASLRTVRSNVAVLAHQQCRGAAKQGPVGNL------STSHQPA----PPAEDTGQMLALETLDELDWCLDQLETLQTRHSVGEMASNKFKRILNRELTHLSETSRSGNQVSEYISQTFLDQQTEVELPTVTAE-EPPK-------------------PVSQISG-----LHGLCHSASLS-SATVPRFGVQTEQEELLAKELEDTNKWG-LDVFKVAELSGNRPLTAIIFSIFQRRDLLKTFQIPADTLATYLLLLEGHYHADVAYHNSLHAADVAQSTHVLLATPALEAVFTDLEVLAALFASAIHDVDHPGVSNQFLINTNSELALMYNDASVLENHHLAVGFKLLQAENCDIFQNLSTKQRLSLRRMVIDMVLA
Mmus_PDE4C FDLENGLPG--GKGLLDAQSGPSLG-RALQPPVHH-VQRRESFLYRSDSDHEPS-PKAVSRTSSAASD-LHGEDMIVTPFAQ---------VLASLRTVRNNVAALAHGPG-SATRQVLLGTP------PHSSQQA----APTEDSGLQLVQETLEELDWCLEQLETLQTRRSVGEMASNKFKRMLNRELSYLSETSRSGNQVSEYISQTFLDQQAEVELPQPPTE-DDPW-------------------PMAQITE-----LRRSSHTS-LP-TAAIPRFGVQTDQEEQLAKELEDTNKWG-LDVFKVAELSGNRPLTAVIFSVFQERDLLKTFQIPADTLLAYLLTLEGHYHSDVAYHNSMHAADVVQSAHVLLGTPALEAVFTDLEVLAAIFACAIHDVDHPGVSNQFLINTNSELALMYNDSSVLENHHLAVGFKLLQGENCDIFRNLSTKQRLSLRRMVIDMVLA
Rnor_PDE4C FDLENGLPS--GRGPLDAQSGSSLG-RALQPPVHH-GQRRESFLYRSDSDHEPS-PKAVSRTSSAASD-LHGEDMIVTPFAQ---------VLASLRTVRSNVAALAHGAG-SATRQALLGTP------PQSSQQA----APAEESGLQLAQETLEELDWCLEQLETLQTRRSVGEMASNKFKRMLNRELTHLSETSRSGNQVSEYISQTFLDQQAEVELPAPPTE-DHPW-------------------PMAQITG-----LRKSCHTS-LP-TAAIPRFGVQTDQEEQLAKELEDTNKWG-FDVFKVAELSGNRPLTAVIFSVLQERDLLKTFQIPADTLLRYLLTLEGHYHSNVAYHNSIHAADVVQSAHVLLGTPALEAVFTDLEVLAAIFACAIHDVDHPGVSNQFLINTNSELALMYNDSSVLENHHLAVGFKLLQGENCDIFQNLSTKQKLSLRRMVIDMVLA
Cfam_PDE4C FDLENGLSC--GRSTLDPQAGPGLG-RVIQASAQH-SQRRESFLYRSDSDYELS-PKAMSRNSSVASD-LHGEDMIVTPFAQ---------VLASLRTVRSNVAALAHLQGRGAAKQASVENP------LFGSQPP----PPTEDTGQKLALETLDELDWCLDQLETLQTRHSVGEMASNKFKRMLNRELTHLSETSRSGNQVSEYISQTFLDQQTEMELPRVTPM-ETPR-------------------PMSQISG-----LRGLPHSASLS-AATIPRFGVQTDQEGQLAKELEDTNKWG-LDVFKVAELSGNRPLTTIIFSIFQERDLLKTFQIPADTLATYLLMLESHYHADVAYHNSLHAADVAQSTHVLLATPALEAVFTDLEVLAAVFASAIHDVDHPGVSNQFLINTNSELALMYNDASVLENHHLAVGFKLLQAENCDIFQNLSAKQRQSLRRMVIDMVLA
Ecab_PDE4C FDLENGLSS--GRSPLDPQAGPSLG-RIVQVAAQH-SQRRESFLYRSDSDFQLL-PKATSWNSSVASD-LQGEDMIVTPFAQ---------VLASLRTVRSNVAALAHLQGRGATKQAPVGKP------PSGSQAP----PSTEDPGQKLALETLDELDWCLDQLETLQTRHSVGEMASNKFKRMLNRELTHLSETSRSGNQVSEYISRTFLDQQTEVELPRVTPT-EPPK-------------------PISQISG-----LRGLPHSASLS-AATFPRFGVQTDQEGQLAKELEDTNKWG-LDVFKVAELSGNRPLTAIIFSIFQERDLLKTFQIPADTLVTYLLTLEGHYRADVAYHNSLHAADVAQSTHVLLATPALQAVFTDLEVLAAIFASAIHDVDHPGVSNQFLINTNSELALMYNDASVLENHHLAVGFKLLQAENCDIFQNLSAKQRLSLRRMVIDMVLA
Btau_PDE4C FDLENGLSC--GRSILDPRAGSGFG-QIVQAPAQH-SQRRESFLYRSDSDYELS-SKTMSRNSSVASD-LHGEDLIVTPFAQ---------VLASLRTVRSNVAALARLQDRGAAKQACVCNT------PSGSQPP----PPK-DTGQKLALETLDELDWCLDQLETLQTRHSVGEMASNKFKRMLNRELTHLSETSRSGNQVSEYISQTFLEQQTEVELPRATPA-EPPR-------------------PVSQISS-----LRGLPHSSSLS-TATVPRFGVQTDQEGQLAKELEDTSKWG-LDVFKVAELSGNRPLTAIMFSIFQERDLLKTFQIPADTLATYLLMLEGHYHNDVAYHNSLHAADVAQSTHVLLATPALEAVFTDLEVLAAIFASAIHDVDHPGVSNQFLINTNSELALMYNDASVLENHHLAVGFKLLQAENCDIFQNLSTKQRLSLRKMVIDIVLA
Mdom_PDE4C FDLENGLSS--GRSPLDPQTSPGSG-LVIQGNFPH-SQRRESFLYRSDSDYDLS-PKAMSRNSSTASD-LHGEDMIVTPFDPGGGWCLPSQVLASLRTVRSNFAVLTRLQDRV---------------AKPTSDLKV---PPTEDACQKLAMETLEELDWCLDQLETLQTRHSVSEMASNKFKRMLNRELTHLSETSRSGNQVSEYISRTFLGELRTREQVDGGSETDLRKKTKTLG-------------KGQLISG-----VRKLTHGSSFS-AAAIPRFGVKTDHETLLAKELKDTNKWG-LDVFKVAELSGNRPLTAIMYSIFQERDLLKTFRIPVDTLVTYLLTLEDHYHADVAYHNSIHAADVAQSTHVLLSTPALEAVFTDLEILAAIFASAIHDVDHPGVSNQFLINTNSELALMYNDASVLENHHLAVGFKLLQEDNCDIFQNLRPKQRQSLRKMVIDMVLA
Xtro_PDE4C --------------------------LVLQGNFPH-SQRRESFLYRSDSDYDLS-PKAMSRNSSIASD-LHGEDMIVTPFAQ---------VLASLRTVRSNFAMLTHLQDRVSNKRSSGSNQ------PSACKP-----NLSEDAVQKLAIETLEELDWCLDQLETLQTRHSVSEMASNKFKRMLNRELTHLSETSRSGNQVSEYISSTFLDKQHEVEIPTPVS-KEKDKKKR----------------PMSQISG-----VKKLTHSSSFS-NCSIPRFGVKTEQEVPLAKEVENINKWG-LDIFKVAEYSGNRPLTVIMYSIFQERDLLKTFHIPVDTFITYMMTLEDHYHSDVAYHNNIHAADVAQSTHVLLSTPALEAVFTDLEIMAAIFASAIHDVDHPGVSNQFLINTNSELALMYNDASVLENHHLAVGFKLLQEENCDIFQNLTKKQRQTLRKMVIDMVLA
Olat_PDE4C1 FDVENGLSV--GRSPLDSQTSPGSG-LVLQGNFPH-SQRRESFLYRSDSDFDLS-PKAMSRNSSTASD-LHGEDMIVTPFAQ---------VLASLRTVRSNFAVLKKKNNLHLCSRWPPGST-----QPPICKPCL---AE--EPYQKLAMETLEELDWCLDQLETLQTRHSVSDMASNKFKRMLNRELTQLSETSRSGNQVSEFIANTFLEKQHDVEILSPPL-KEKEKKKR----------------PMSQISG-----VKKATQSPSLP-PASISRFGVNTPHESLLAKEIEDIDRWG-MDIFKIAEYSGNRPLTVIMYSIFQERDLLKTFKIQADTFITFMMTLEDHYHADVAYHNNIHAADVVQSTHVLLSTPALEAVFTDLEIMAALFASAIHDVDHPGVTNQFLINTSSELALMYNDASVLENHHLAVGFKLLQEENCDIFENLSKKQKDSLRKMVIDMVLA
Trub_PDE4C1 FDVENGLSV--GRSPLDPQASPGSG-LVIQANFPH-SQRRESFLYRSDSDFDLS-PKGPSRNSSTASD-LHTEDMIVTPFAQ---------VLASLRTVRGNFAIITNQQDRPVSKTRSAGSN-----PPSMCKTSL---A--EEPHQQLAIETLDELDWCLEQLETLKTRHSVSEMASNKFKRMLNRELSQLSETSRSGNQVSEFISSTFLEKQHDMDIMSTPT-KEKDKKKR----------------PMSQISG-----VKKATHSPSLA-PSTIPRFGVNASQEGLLAKEMEDVNRWG-IDIFKIAEYSGRRPLTVTMYTIFQERELLKSFKIPTDTFLTFMMTLEDHYHADVAYHNNIHAADVVQSTHVLLSTPALEAVFTDLEILAALFASAIHDVDHPGVSNQFLINTNSELALMYNDSSVLENHHLAVGFKLLQEDNCDIFQNLSKKQRQSLRKMVIDMVLA
Trub_PDE4C2 FDVENGLSV--GRSPLDSQTSPGSG-LVLQANFPH-SQRRESFLYRSDSDFDLS-PKTMSRNSSTASD-LHGEDMIVTPFAQ---------VLASLRTVRGNFVALTHLQDRVGNKRQ-TGSN-----QPPVCKPCV---T--EEPCPKLALETLEELDWCLDQLETLQTRHSVSEMASNKFKRMLNRELTQLSETSRSGNQVSEFIANTFLEKQHDVEILSPPP-KEREKKKR----------------PMSQISG-----VKKVIP--SLA-SVSIPRFGVNTPQESQLAKELEEINRWG-LDMFKISEFSGNRPLTVIMYSVFQERDLLKTFKIPVGTFVTFVMTLEDHYHGDVAYHNNIHAADVVQSTHVLLSTPALEAVFTDLEIMAALFASAIHDVDHPGVTNQFLINTSSELALMYNDASVLENHHLAVGFKLLQEENCDIFQNLSKKQKDSLRKMVIDMVLA
Drer_PDE4C1 FDVENGLSV--GRSPLDSQASPGSG-LVLQANFPH-SQRRESFLYRSDSDFDLS-PKAMSRNSSTASE-LHGEDMIVTPFAQ---------VLASLRTVRSNFAVLTHQQDRVPSKK-STGNN-----PPSMCKTSL---P--EEPFQKLAVETLDELDWCLEQLETLKTRHSVSEMASNKFKRMLNRELTQLSETSKSGNQVSEYISSTFLEKQHDMDIMSTPS-KEKEKKKR----------------PMSQISG-----VKKPTHSSNIA-PSSIPRFGVSTNREDLLAKELEDFDRWG-VDMFKISECSGNRPLTVAMYTTFQERDLLKSFKIPVDTFINYMTALEENYRSDVAYHNSIHAADVVQSTHVLLSTPALEAVFTDLEILAAMFASAIHDVDHPGVSNQFLINTNSELALMYNDSSVLENHHLAVGFKLLQEENCDIFQNLTKKQRQSLRKMVIDMVLA
Drer_PDE4C2 FDVENGLSM--GRSSLDPSPSS--G-LVLQANS----QRRESFLYRSDSDFDLS-PKTMSRNSSTASE-LHGEDMIVTPFAQ---------VLASLRTVRGNVAALTHMQDRSNKRPS--GTN-----PQSACKTNL---S--DEAYQKLTVETLEELDWCLDQLETLQTRHSVSEMASNKFKRMLNRELTQLSETSRSGNQVSEFIASTILQKQYDVELLSAAC-KE-EKKRR----------------PMSQISG-----VRKLSPSPSLP-PTCIPRFGVNTQHESLLAKELEDLDRWG-IDIFKIAEYSGNRPLTVMMYTIFQERDLLKTFKIPSDTFLTFLMTLEDHYHADVAYHNNIHAADVVQSTHVLLSTPALEDVFTDLEIMAALFASAIHDVDHPGVTNQFLINTNSELALMYNDASVLENHHLAVGFKLLQEENCDIFCNLSKKQRQSLRQMTIDMVLA
Gacu_PDE4C FDVENGLAV--GRSPLDPQTSPGSGGLVLQANFPH-SQRRESFLYRSDSDFDLS-PKGPSRNSSAASD-LHTEDMIVTPFAQ---------VLASLRTVRSNFAVITDQQDRTASKTRSSGSN-----PPSMCKTSL---AGTEEPHQQLAIETLDELDWCLEQLETLKTRHSVSEMASNKFKRMLNRELTQLSETSRSGNQVSEFISSTFLEKQHDMDIMSPPT-KEKDKKKR----------------PMSQISG-----VKKATHSPSLA-PSTIPRFGVNASQEGLLAKELEEVNRWG-IDIFKVSEYSGNRPLTVTMYTIFQERDLLKSFKIPADTFITFMMTLEDHYHADVAYHNNIHAADVVQSTHVLLSTPALEAVFTDLEILAALFSSAIHDVDHPGVSNQFLINTNSELALMYNDSSVLENHHLAVGFKLLQEDNCDIFQNLGKKQRQSLRKIVIDMVLA
Hsap_PDE4D FDVDNGTSA--GRSPLDPMTSPGSG-LILQANFVH-SQRRESFLYRSDSDYDLS-PKSMSRNSSIASD-IHGDDLIVTPFAQ---------VLASLRTVRNNFAALTNLQ-DRAPSKRSPMCNQ-----PSINKA-----TITEEAYQKLASETLEELDWCLDQLETLQTRHSVSEMASNKFKRMLNRELTHLSEMSRSGNQVSEFISNTFLDKQHEVEIPSP-TQKEKEKKKR----------------PMSQISG-----VKKLMHSSSLT-NSSIPRFGVKTEQEDVLAKELEDVNKWG-LHVFRIAELSGNRPLTVIMHTIFQERDLLKTFKIPVDTLITYLMTLEDHYHADVAYHNNIHAADVVQSTHVLLSTPALEAVFTDLEILAAIFASAIHDVDHPGVSNQFLINTNSELALMYNDSSVLENHHLAVGFKLLQEENCDIFQNLTKKQRQSLRKMVIDIVLA
Gacu_PDE4D FDVDNGTSS--GRSPLDPMASPGSG-LILQANFVH-SQRRESFLYRSDSDYDLS-PKSMSRNSSIASD-IHGDDMIVTPFAQ---------VLASLRTVRNNFGALTNLQQDRASNKRSPMCNP-----PPITKT-----TFTEEAYQKLATETLEELDWCLDQLETLQTRHSVSEMASNKFKRMLNRELTHLSEMSRSGNQVSEFISSTFLDKQHEVEMPTPQTQKEKEKKNK----------------PMSQIIG-----VKKLQHSSSLT-NSNIPRFGVKSETEDELAKELEHVNKWG-RNVFKIAEFSGNRPLTVMMYTIFQERDLLKTFKIPLDTFITYLMTLEDHYHGDVAYHNNIHAADVTQSTHVLLSTPALEAVFTDLEILAAIFASAIHDVDHPGVSNQFLINTNSELALMYNDSSVLENHHLAVGFKLLQEENCDIFQNLNKKQRQSLRKMVIDIVLA
Olat_PDE4D FDVDNGTSS--GRSPLDPMASPGSG-LILQANFVH-SQRRESFLYRSDSDYDLS-PKSMSRNSSIASD-IHGDDMIVTPFAQ---------VLASLRTVRNNFGALTNVQQERASTKRSPMCNP-----PPITKT-----TFTEEAYQKLATETLEELDWCLDQLETLQTRHSVSEMASNKFKRMLNRELTHLSEMSRSGNQVSEFISSTFLDKQHEVEMPTPQTQKEKEKKNK----------------PMSQISG-----VKKLQHSSSLT-NSNIPRFGVKTETEDELAKELEHVNKWG-LNVFKITEFSGNRPLTVMMHTIFQERDLLKTFKIPLDTFITYLLTLEDHYHGDVAYHNNIHAADVTQSTHVLLSTPALEAVFTDLEILAAIFASAIHDVDHPGVSNQFLISTNSELALMYNDSSVLENHHLAVGFKLLQEENCDIFQNLTKKQRQSLRKMVIDIVLA
Trub_PDE4D FDVDNGTSS--GRSPLDPMASPGSG-LILQANFVH-SQRRESFLYRSDSDYDLS-PKSMSRNSSIASD-IHGDDMIVTPFAQ---------VLASLRTVRNNFGALTNLQQDRASNKRSPMCNP-----PPITKT-----TFTEEAYQKLATETLEELDWCLDQLETLQTRHSVSEMASNKFKRMLNRELTHLSEMSRSGNQVSEFISSTFLDKQHEVEMPCPQTQKEKEKKNK----------------PMSQISG-----VKKLQHSSSLT-NSNIPRFG-KTETEDELAKELEHVNKWG-LNVFKISEFSGNRPLTVMMYTIFQERDLLKTFKIPLDTFITYLMTLEDHYHGDVAYHNNIHAADVTQSTHVLLSTPALEAVFTDLEILAAIFASAIHDVDHPGVSNQFLINTNSELALMYNDSSVLENHHLAVGFKLLQDENCDIFQNLTKKQRQLLRKMVIDIVLA
Acar_PDE4D FDVDNGTSS--GRSPLDPMTSPGSG-LILQANFVH-SQRRESFLYRSDSDYDLS-PKSMSRNSSIASD-IHGDDLIVTPFAQ---------VLASLRTVRNNFAALTNLQ-DRAPSKRSPMCNQ-----PSINKA-----TITEEAYQKLASETLEELDWCLDQLETLQTRHSVSEMASNKFKRMLNRELTHLSEMSRSGNQVSEYISNTFLDKQHEVEIPSP-TQKEKEKKKR----------------PMSQISG-----VKKLMHSSSLT-NSSIPKFGVKTDQEDILAKELEDVNKWG-LQVFKIAEFSGNRPLTVIMYTIFQERELLKTFKIQPDMLITYLMTLEDHYHADVAYHNNIHAADVVQSTHVLLSTPALEAVFTDLEILAAIFASAIHDVDHPGVSNQFLINTNSELALMYNDSSVLENHHLAVGFKLLQEENCDIFQNLTKKQRQSLRKMVIDIVLA
Cfam_PDE4D FDVDNGTSA--GRSPLDPMTSPGSG-LILQANFVH-SQRRESFLYRSDSDYDLS-PKSMSRNSSIASD-IHGDDLIVTPFAQ---------VLASLRTVRNNFAALTNLQ-DRAPSKRSPMCNQ-----PSINKA-----TITEEAYQKLASETLEELDWCLDQLETLQTRHSVSEMASNKFKRMLNRELTHLSEMSRSGNQVSEYISNTFLDKQHEVEIPSP-TQKEKEKKKR----------------PMSQISG-----VKKLMHSSSLT-NSSIPRFGVKTEQEDVLAKELEDVNKWG-LHVFRIAELSGNRPLTVIMHTIFQERDLLKTFKIPVDTLITYLMTLEDHYHADVAYHNNIHAADVVQSTHVLLSTPALEAVFTDLEILAAIFASAIHDVDHPGVSNQFLINTNSELALMYNDSSVLENHHLAVGFKLLQEENCDIFQNLTKKQRQSLRKMVIDIVLA
Btau_PDE4D FDVDNGTSS--GRSPLDPMTSPGSG-LILQANFVH-SQRRESFLYRSDSDYDLS-PKSMSRNSSIASD-IHGDDLIVTPFAQ---------VLASLRTVRNNFAALTNLQ-DRAPSKRSPMCNQ-----PSINKA-----TLTEEAYQKLASETLEELDWCLDQLETLQTRHSVSEMASNKFKRMLNRELTHLSEMSRSGNQVSEYISNTFLDKQHEVEIPSP-TQKEKEKKKR----------------PMSQISG-----VKKLMHSSSLT-NSSIPRFGVKTEQEDVLAKELEDVNKWG-LHVFRIAELSGNRPLTVIMHTIFQERDLLKTFKIPVDTLITYLMTLEDHYHADVAYHNNIHAADVVQSTHVLLSTPALEAVFTDLEILAAIFASAIHDVDHPGVSNQFLINTNSELALMYNDSSVLENHHLAVGFKLLQEENCDIFQNLTKKQRQSLRKMVIDIVLA
Mdom_PDE4D FDVDNGTSS--GRSPLDPMTSPGSG-LILQANFVH-SQRRESFLYRSDSDYDLS-PKSMSRNSSIASD-IHGDDLIVTPFAQ---------VLASLRTVRNNFAALTNLQ-DRAPNKRSPMCNQ-----PSINKA-----TITEEAYQKLASETLEELDWCLDQLETLQTRHSVSEMASNKFKRMLNRELTHLSEMSRSGNQVSEFISSTFLDKQHEVEIPSP-TQKEKEKKKR----------------PMSQISG-----VKKLMHSSSLT-NSSIPRFGVKTDQEDGLAKELEDVNKWG-LHVFKIAEFSGNRPLTVIMHTIFQERDLLKTFKIPVDTLITYLMTLEDHYHADVAYHNNIHAADVVQSTHVLLSTPALEAVFTDLEILAAIFASAIHDVDHPGVSNQFLINTNSELALMYNDSSVLENHHLAVGFKLLQEENCDIFQNLTKKQRQSLRKMVIDIVLA
Ecab_PDE4D FDVDNGTSS--GRSPLDPMTSPGSG-LILQANFVH-SQRRESFLYRSDSDYDLS-PKSMSRNSSIASD-IHGDDLIVTPFAQ---------VLASLRTVRNNFAALTNLQ-DRAPSKRSPMCNQ-----PSINKA-----TITEEAYQKLASETLEELDWCLDQLETLQTRHSVSEMASNKFKRMLNRELTHLSEMSRSGNQVSEYISNTFLDKQHEVEIPSP-TQKEKEKKKR----------------PMSQISG-----VKKLMHSSSLT-NSSIPRFGVKTEQEDVLAKELEDVNKWG-LHVFRIAELSGNRPLTVIMHTIFQERDLLKTFKIPVDTLITYLMTLEDHYHADVAYHNNIHAADVVQSTHVLLSTPALEAVFTDLEILAAIFASAIHDVDHPGVSNQFLINTNSELALMYNDSSVLENHHLAVGFKLLQEENCDIFQNLTKKQRQSLRKMVIDIVLA
Rnor_PDE4D FDVDNGTSA--GRSPLDPMTSPGSG-LILQANFVH-SQRRESFLYRSDSDYDLS-PKSMSRNSSIASD-IHGDDLIVTPFAQ---------VLASLRTVRNNFAALTNLQ-DRAPSKRSPMCNQ-----PSINKA-----TITEEAYQKLASETLEELDWCLDQLETLQTRHSVSEMASNKFKRMLNRELTHLSEMSRSGNQVSEYISNTFLDKQHEVEIPSP-TQKEKEKKKR----------------PMPQISG-----VKKLMHSSSLT-NSCIPRFGVKTEQEDVLAKELEDVNKWG-LHVFRIAELSGNRPLTVIMHTIFQERDLLKTFKIPVDTLITYLMTLEDHYHADVAYHNNIHAADVVQSTHVLLSTPALEAVFTDLEILAAIFASAIHDVDHPGVSNQFLINTNSELALMYNDSSVLENHHLAVGFKLLQEENCDIFQNLTKKQRQSLRKMAIDIVLA
Mmus_PDE4D FDVDNGTSA--GRSPLDPMTSPGSG-LILQANFVH-SQRRESFLYRSDSDYDLS-PKSMSRNSSIASD-IHGDDLIVTPFAQ---------VLASLRTVRNNFAALTNLQ-DRAPSKRSPMCNQ-----PSINKA-----TITEEAYQKLASETLEELDWCLDQLETLQTRHSVSEMASNKFKRMLNRELTHLSEMSRSGNQVSEYISNTFLDKQHEVEIPSP-TQKEKEKKKR----------------PMSQISG-----VKKLMHSSSLT-NSCIPRFGVKTEQEDVLAKELEDVNKWG-LHVFRIAELSGNRPLTVIMHTIFQERDLLKTFKIPVDTLITYLMTLEDHYHADVAYHNNIHAADVVQSTHVLLSTPALEAVFTDLEILAAIFASAIHDVDHPGVSNQFLINTNSELALMYNDSSVLENHHLAVGFKLLQEENCDIFQNLTKKQRQSLRKMVIDIVLA
Cjac_PDE4D FDVDNGTSA--GRSPLDPMTSPGSG-LILQANFVH-SQRRESFLYRSDSDYDLS-PKSMSRNSSIASD-IHGDDLIVTPFAQ---------VLASLRTVRNNFAALTNLQ-DRAPSKRSPMCNQ-----PSINKA-----TITEEAYQKLASETLEELDWCLDQLETLQTRHSVSEMASNKFKRMLNRELTHLSEMSRSGNQVSEYISNTFLDKQHEVEIPSP-TQKEKEKKKR----------------PMSQISG-----VKKLMHSSSLT-NSSIPRFGVKTEQEDVLAKELEDVNKWG-LHVFRIAELSGNRPLTVIMHTIFQERDLLKTFKIPVDTLITYLMTLEDHYHADVAYHNNIHAADVVQSTHVLLSTPALEAVFTDLEILAAIFASAIHDVDHPGVSNQFLINTNSELALMYNDSSVLENHHLAVGFKLLQEENCDIFQNLTKKQRQSLRKMVIDIVLA
Drer_PDE4D FDVDNGTSS--GRSPLDPMASPGSG-LILQANFVH-SQRRESFLYRSDSDYDLS-PKSMSRNSSIASD-IHGDDMIVTPFAQ---------VLASLRTVRNNFAALTNVQQERSNNKRSPMCNP-----PPITKT-----SYTEEAYQKLATETLEELDWCLDQLETLQTRHSVSEMASNKFKRMLNRELTHLSEMSRSGNQVSEYISSTFLDKQNEVEMPSPQSQKDKEKKKR----------------PMSQISG-----VKKLTHSSSLT-NSNIPRFGVKTETEDELAKELEDVNKWG-LNVFKVSEFSGNRPLTVMMYTIFQERDLLKTFKIPLDTFITYLMTLEDHYHADVAYHNNIHAADVTQSTHVLLSTPALEAVFTDLEILAAIFASAIHDVDHPGVSNQFLINTNSELALMYNDSSVLENHHLAVGFKLLQEENCDIFQNLTKKQRQSLRKMVIDIVLA
Cint_PDE4 TDMSKHMQLLANLKTMVETKKVAASGVLMLDNYTDR---------IQVLQNMVHCADLSNSSKPIEAYKQWVARLIDEFWMQGDVEREAGLEISPLCDRHNTSIERSQVMFIDFVVHPLLETWADLVNPYAQDIINQLASNRE-YYASRLPNLPSPNETS------------------------DCDVTVVDDSSTETEGDESDDDVIQTPVPTSQLQNNRFQFQISLDEDSSVSELSERRKSREMTNKPPR--------------------------------------------------------------------------------------------------------------------------------------------------------------------------------------------
Hsap_PDE4A TDMSKHMTLLADLKTMVETKKVTSSGVLLLDNYSDR---------IQVLRNMVHCADLSNPTKPLELYRQWTDRIMAEFFQQGDRERERGMEISPMCDKHTASVEKSQVGFIDYIVHPLWETWADLVHPDAQEILDTLEDNRDW-YYSAIRQSPSPPPEEE--------------SRGPGHPPLPDKFQFELTLEEEEEEEISMAQIPCTAQEALTAQGLSGVEEALDATIAWEASPAQESLEVMAQEASLEAELEAVYLTQQAQSTGSAPVAPDEFSSREEFVVAVSHSSPSALALQSPLLPAWRTLSVSEHAPGLPGLPSTAAEVEAQREHQAAKRACSACAGTFGEDTSALPAPGGGGSGGDPT*------------------------------------------------------------------------
Cjac_PDE4A TDMSKHMTLLADLKTMVETKKVTSSGVLLLDNYSDR---------IQVLRNMVHCADLSNPTKPLELYRQWTDRIMAEFFQQGDRERERGMEISPMCDKHTASVEKSQVGFIDYIVHPLWETWADLVHPDAQEILDTLEDNRDW-YYSAIRQSPSPPPEGE--------------SRGPGHPPPPDKFQFELTLEEEEEEETSVAQEALTSQGLSAAEGALDATMDPSPAQESLEVMAQDTPLEAELEEVDLTQLAESTGSAPVAQDEFSSQEESVVAVSCGSPSALSLQSPLLPAWRTLSVSEDAPGLPGLPSTAAEVEAQREHQAAKRACSACAETSGEETSAFPAPGWWGSDGDPT*--------------------------------------------------------------------------------
Mmus_PDE4A TDMSKHMTLLADLKTMVETKKVTSSGVLLLDNYSDR---------IQVLRNMVHCADLSNPTKPLELYRQWTDRIMAEFFQQGDRERERGMEISPMCDKHTASVEKSQVGFIDYIVHPLWETWADLVHPDAQDILDTLEDNRDW-YHSAIRQSPSPTLEEE--------------PGVLSDPALPDKFQFELTLEEEDEEDSLEVPGLPCTEETLLAPHDTRAQAMEQSKVKGQSPAVVEVAESLKQETASAHGAPEESAEAVGHSFSLETSILPDLRTLSPSEEAQGLLGLPSMAAEVEAPRDHLAAMRACSACSGTSGDNSAVISAPGRWGSGGDPA*----------------------------------------------------------------------------------------------------
Rnor_PDE4A TDMSKHMTLLADLKTMVETKKVTSSGVLLLDNYSDR---------IQVLRNMVHCADLSNPTKPLELYRQWTDRIMAEFFQQGDRERERGMEISPMCDKHTASVEKSQVGFIDYIVHPLWETWADLVHPDAQDILDTLEDNRDW-YHSAIRQSPSPPLEEE--------------PGGLGHPSLPDKFQFELTLEEEEEEDSLEVPGLPTTEETFLAAEDARAQAVDWSKVKGPSTTVVEVAERLKQETASAYGAPQESMEAVGCSFSPGTPILPDVRTLSSSEEAPGLLGLPSTAAEVEAPRDHLAATRACSACSGTSGDNSAIISAPGRWGSGGDPA*----------------------------------------------------------------------------------------------------
Cfam_PDE4A TDMSKHMTLLADLKTMVETKKVTSSGVLLLDNYSDR---------IQVLRNMVHCADLSNPTKPLELYRQWTDRIMAEFFQQGDRERERGMEISPMCDKHTASVEKSQVQFYSPIVHPLWETWADLVHPDAQEILDTLEDNRDW-YYSAIRQSPSPPPEEE--------------PRRPGHPPPPDKFQFELTLEEEEEEEVSTAPGAFEVQGLSMAQDPSPAEDLLEA-------------------------------------------------------------------------------------------------------------------------------------------------------------------------------------------------------------------
Btau_PDE4A TDMSKHMTLLADLKTMVETKKVTSSGVLLLDNYSDR---------IQVLRNMVHCADLSNPTKPLELYRQWTDRIMAEFFQRGDRERERGMEISPMCDKHTASVEKSQVGFIDYIVHPLWETWADLVHPDAQEILDTLEDNRDW-YYSAIRQSPSPPPEEE--------------PGGPGHPPPPDKFQFELTLDEEEEEEVCTAPGAVAIQELYMAQDASPPEDPLEVDGQDPYAEVEIEEMYLTRQVDSTGSVAAGQDVSVPQGASVDVCVGCRNIPALSPNSLAPLALRTPPPPEDDPGLLGLPSMAAEVGAQKEHQAAKRACCACTGTAGEDPTPGALPAPGAWGSAGGPT*------------------------------------------------------------------------------------
Sscr_PDE4A TDMSKHMTLLADLKTMVETKKVTSSGVLLLDNYSDR---------IQVLRNMVHCADLSNPTKPLELYRQWTDRIMAEFFQQGDRERERGMEISPMCDKHTASVEKSQVGFIDYIVHPLWETWADLVHPDAQEILDTLEDNRDW-YYSAIRQSPSPPPEEE--------------PGGPGHPPLPDKFQFELTLEEEEEEEVSTGPGAVKVQELSTAPDAEQPLEVEEMYLTQRADLADSVATGGQDGPTSPDASLDAVGCSSPPALSSESFPLPALRTLPPPEGDPGLLGLPSMAAEVGAQKEHQAAKRACCACTGTSAEDPPTLSAPGGWGPAGGPT*----------------------------------------------------------------------------------------------------
Mdom_PDE4A TDMSKHMSLLADLKTMVETKKVTSSGVLLLDNYTDR---------IQVLRNMVHCADLSNPTKPLALYRQWTDRIMEEFFRQGDKERERGMEISPMCDKHTASVEKSQVGFIDYIVHPLWETWADLVHPDAQEILDMLEDNRDW-YHRAIPQSPSPPPEEP--------------PG-----LSQDKFQFELIHEEDATTTKTATLLEEKDEPAPEVGRVGPGHGPEEGSLPAPISTRPLSPSRAGAIYQSCGSPDGMEQEKQQTQEPLAPEEVQILLASPSEETEASEAVAEAKAEAPSLAEEEIAEEAQPWTPPSPPTTSTFFQQQQQQQQHLWRQWRRQWRHRQQQEQEQQQQQQQQPQQQQKLATRTAGLALLPQTRSFLSPTPTPIPFCLSPSPRPNPASHPKRPAVSCFFPRPSWRLLPPSPSALVLAPSSPP*
Acar_PDE4A TDMSKHMSLLADLKTMVETKKVTSSGVLLLDNYTDR---------IQVLRNMVHCADLSNPTKPLELYRQWTDRIMEEFFRQGDKERERGMEISPMCDKHTASVEKSQVGFIDYIVHPLWETWADLVHPDAQDILDTLEDNRDW-YHSMIPQSPSPPPDD----------------AEKDDEACLEKFQFELTLEEEGEDSEQSDKDCSSPGDEDNSYYTAAEEHPHESLEGQDRLTDDTSPGAS*L*GRDNGKGGKNKTTYIHTATHREKV------------------------------------------------------------------------------------------------------------------------------------------------------------------------
Xlae_PDE4A TDMSKHMSLLADLKTMVETKKVTSSGVLLLDNYTDR---------IQVLRNMVHCADLSNPTKPLELYRQWTDRILEEFFRQGDKERERGMEISPMCDKHTASVEKSQVGFIDYIVHPLWETWSDLVHPDAQDILDTLEDNRDW-YQGMIPQSPSPPPDD----------------PDKDEEACIEKFQFELTLEEEAEDSDKDRNSGIEEEDGHCLHEATEIQELEEEFMAMHEGQTEQTDVYSDNSSPVEDS*-----------------------------------------------------------------------------------------------------------------------------------------------------------------------------------------
Gacu_PDE4A1 TDMSKHMSLLADLKTMVETKKVTSSGVLLLDHYTDR---------IQVLRNMVHCADLSNPTKPLAVYRQWTERIMEEFFRQGDKERERGMEISPMCDKHTASVEKSQVGFIDYIVHPLWETWGDLVHPDAQDILDTLEDNRDW-YQSTIPQSPSPPPVC----------------QDKDLNACIDKFQFELTLEDSSPREEGDEGEHTANHVEQDCNGKKEEDVMGQLEVRYEKERLSDTSPVEEEEDSSSQAEDT*--------------------------------------------------------------------------------------------------------------------------------------------------------------------------------------
Gacu_PDE4A2 TDMSKHMTLLADLKTMVETKKVTSSGVLLLDHYTER---------IQVLRNMVHCADLSNPTKTLAIYRQWTERIMEEFFRQGDKERERGMEISAMCDKHTASVEKSQVGFIDYIVHPLWETWADLVHPDAQELLDTLEENREW--------YLSTMPQS--------------PSPPPDRHLQHDRFQFELTLED----------------------------------------------------------------------------------------------------------------------------------------------------------------------------------------------------------------------------------------------------
Trub_PDE4A1 TDMSKHMSLLADLKTMVETKKVTSSGVLLLDNYTDR---------IQVLRNMVHCADLSNPTKPLELYRQWTDRILEEFFRQGDKERERGMEISPMCDKHTASVEKSQVGFIDYIVHPLWETWADLVHPDAQDILDTLEDNRDW-YQGMIPQSPSPPPDD----------------PDKDEEACIEKFQFELTLEEEAEDSDKDRNSGIEEEDGNCLHEAPEIQEPEEEFMTMHEGQAEQTDVYSDNTSPVEDS*-----------------------------------------------------------------------------------------------------------------------------------------------------------------------------------------
Trub_PDE4A2 TDMSKHMTLLADLKTMVETKKVTSSGVLLLDHYTER---------IQVLRNMVHCADLSNPTKTLQLYRQWTERIMEEFFRQGDKERERGMEISAMCDKHTASVEKSQVGFIDYIVHPLWETWADLVHPDAQELLDTLEENRNW--------YLNIIPQS--------------PSPPPDRHLQHDRFQFELTLED----------------------------------------------------------------------------------------------------------------------------------------------------------------------------------------------------------------------------------------------------
Olat_PDE4A2 TDMSKHMTLLADLKTMVETKKVTSSGVLLLDHYTER---------IQLLKNMVHCADLSNPTKPLPLYRQWTERIMEEFFRQGDKERERGMEISAMCDKHTASVEKSQVGFIDYIVHPLWETWADLVHPDAQELLDTLEENREW--------YLTTMPQS--------------PSPPPDRHLQHDRFQFEITLED----------------------------------------------------------------------------------------------------------------------------------------------------------------------------------------------------------------------------------------------------
Drer_PDE4A TDMSKHMSLLADLKTMVETKKVTSSGVLMLDHYNDR---------IQVLRNMVHCADLSNPTKPLAVYRQWTERIMEEFFQQGDKERERGMEISPMCDKHTASVEKSQVGFIDYIVHPLWETWGDLVHPDAQEILDTLEDNRDW-YQSTIPQSPSPPPDR----------------PDNELESCRNKFQFELTLEGETEDEAEEKMEDGEEVTEVMVERSGRRRLQVQSVVEEEDERQSDESPVEETEEEEKSSSPTDDT*-----------------------------------------------------------------------------------------------------------------------------------------------------------------------------------
Hsap_PDE4B TDMSKHMSLLADLKTMVETKKVTSSGVLLLDNYTDR---------IQVLRNMVHCADLSNPTKSLELYRQWTDRIMEEFFQQGDKERERGMEISPMCDKHTASVEKSQVGFIDYIVHPLWETWADLVQPDAQDILDTLEDNRNW-YQSMIPQSPSPPLDE----------------QNRDCQGLMEKFQFELTLDEEDSEGPEKEGEGHSYFSSTKTLCVIDPENRDSLGETDIDIATEDKSPVDT*-------------------------------------------------------------------------------------------------------------------------------------------------------------------------------------------------
Cjac_PDE4B TDMSKHMSLLADLKTMVETKKVTSSGVLLLDNYTDR---------IQVLRNMVHCADLSNPTKSLELYRQWTDRIMEEFFQQGDKERERGMEISPMCDKHTASVEKSQVGFIDYIVHPLWETWADLVQPDAQDILDTLEDNRNW-YQSMIPQSPSPPLDE----------------QNRDCQGLMEKFQFELTLDEEDSEGPEREGEGHSYFSSTKTLCVIDPENRDSLGETDIDIATEDTSPVDT*-------------------------------------------------------------------------------------------------------------------------------------------------------------------------------------------------
Mmus_PDE4B TDMSKHMSLLADLKTMVETKKVTSSGVLLLDNYTDR---------IQVLRNMVHCADLSNPTKSLELYRQWTDRIMEEFFQQGDKERERGMEISPMCDKHTASVEKSQVGFIDYIVHPLWETWADLVQPDAQDILDTLEDNRNW-YQSMIPQSPSPPLDE----------------RSRDCQGLMEKFQFELTLEEEDSEGPEKEGEGHSYFSSTKTLCVIDPENRDSLEETDIDIATEDKSPIDT*-------------------------------------------------------------------------------------------------------------------------------------------------------------------------------------------------
Rnor_PDE4B TDMSKHMSLLADLKTMVETKKVTSSGVLLLDNYTDR---------IQVLRNMVHCADLSNPTKSLELYRQWTDRIMEEFFQQGDKERERGMEISPMCDKHTASVEKSQVGFIDYIVHPLWETWADLVQPDAQDILDTLEDNRNW-YQSMIPQSPSPPLDE----------------RSRDCQGLMEKFQFELTLEEEDSEGPEKEGEGPNYFSSTKTLCVIDPENRDSLEETDIDIATEDKSLIDT*-------------------------------------------------------------------------------------------------------------------------------------------------------------------------------------------------
Cfam_PDE4B TDMSKHMSLLADLKTMVETKKVTSSGVLLLDNYTDR---------IQVLRNMVHCADLSNPTKSLELYRQWTDRIMEEFFQQGDKERERGMEISPMCDKHTASVEKSQVGFIDYIVHPLWETWADLVQPDAQDILDTLEDNRNW-YQSMIPQSPSPPLDE----------------QNRDCQGLMEKFQFELTLEEEDSEGPDKEGEGHNYFSSTKTLCVIDPEHRDSLGDTDGDTGAENKTPIDT*-------------------------------------------------------------------------------------------------------------------------------------------------------------------------------------------------
Ecab_PDE4B TDMSKHMSLLADLKTMVETKKVTSSGVLLLDNYTDR---------IQVLRNMVHCADLSNPTKSLELYRQWTDRIMEEFFQQGDKERERGMEISPMCDKHTASVEKSQVGFIDYIVHPLWETWADLVQPDAQDILDTLEDNRNW-YQSMIPQSPSPPLDE----------------QNRDCQGLMEKFQFELTLDEEDSEGPEKEGEGHSYFSSTKTLCVIDPESRDSLGETGVDMATEDKSPVDT*-------------------------------------------------------------------------------------------------------------------------------------------------------------------------------------------------
Btau_PDE4B TDMSKHMSLLADLKTMVETKKVTSSGVLLLDNYTDR---------IQVLRNMVHCADLSNPTKSLELYRQWTDRIMEEFFQQGDKERERGMEISPMCDKHTASVEKSQVGFIDYIVHPLWETWADLVQPDAQDILDTLEDNRNW-YQSMIPQSPSPPLDE----------------QNRDCQGLMEKFQFELTLEEEDSEGPEKDGEGHSYFGSTKTLCVIDPENRDPLGETEVDITTEDKSPIDT*-------------------------------------------------------------------------------------------------------------------------------------------------------------------------------------------------
Sscr_PDE4B TDMSKHMSLLADLKTMVETKKVTSSGVLLLDNYTDR---------IQVLRNMVHCADLSNPTKSLELYRQWTDRIMEEFFQQGDKERERGMEISPMCDKHTASVEKSQVGFIDYIVHPLWETWADLVQPDAQDILDTLEDNRNW-YQSMIPQSPSPPLDE----------------QNRDCQGLMEKFQFELTLEEEDSEGPEKEGEGHGYFGSTKTLCVIDPENRDSLGETGVDTATEDKSPTDT*-------------------------------------------------------------------------------------------------------------------------------------------------------------------------------------------------
Mdom_PDE4B TDMSKHMGLLADLKTMVETKKVTSSGVLLLDNYTDR---------IQVLRNMVHCADLSNPTKSLELYRQWTDRIMEEFFQQGDKERERGMEISPMCDKHTASVEKSQVGFIDYIVHPLWETWADLVQPDAQDILDTLEDNRNW-YQSMIPQSPSPPLEE----------------QNPDCQGLMEKFQFELTLEEEDSEGPEKEGDSVSYLGSTKTLCVIDPEPCEADVETEMGDASPIDT*-----------------------------------------------------------------------------------------------------------------------------------------------------------------------------------------------------
Xlae_PDE4B TDMSKHMSLLADLKTMVETKKVTSSGVLLLDNYTDR---------IQVLRNMVHCADLSNPTKSLELYRQWTDRIMEEFFQQGDRERERGMEISPMCDKHTASVEKSQVGFIDYIVHPLWETWGDLVQPDAQDILDTLEDNRNW-YQSMIPQSPSPLLDE----------------HDKDCQGLSEKFHFELTLEEEDSDGHDKDGDCLSFYSSTKTLHIVDQGMEDSPDADTELLTDDTSPLDT*--------------------------------------------------------------------------------------------------------------------------------------------------------------------------------------------------
Gacu_PDE4B2 TDMSKHMSLLADLKTMVETKKVTSSGVLLLDNYTDR---------IQVLRNMVHCADLSNPTKSLELYRQWTDRIMEEFFHQGDRERERGMEISPMCDKHTASVEKSQVGFIDYIVHPLWETWADLVHPDAQDILDTLEDNRNW-YQSMIPQSPSPPFFD---------QCTLGHGGGTGGQAGGEKFQFELTYSLGDSRSPPPDYSDGQELEEGRMMEPAIEIVTHEASPTDT*-------------------------------------------------------------------------------------------------------------------------------------------------------------------------------------------------------------
Gacu_PDE4B1 TDMSKHMSLLANLKTMVETKKVTSSGVLLLDNYTDR--------MQQVLRNMVHCADLSNPTKPLDLYRQWTDRIMEEFFHQGDRERDRGMEISPMCDKHTASVERTQVGFIDYIVHPLWETWADLVHPDAQDILDTLEDNRNW-YQRMIPQSPSPPFYT--------SDGESGQPGELEGGPVASKFQFELTLDDQDERDAGGENAVMETAGGVILEQHACDGDRVEMTTCDISPAET*--------------------------------------------------------------------------------------------------------------------------------------------------------------------------------------------------------
Olat_PDE4B1 TDMSKHMSLLANLKTMVETKKVTSSGVLLLDNYTDR---------MQVLRNMVHCADLSNPTKPLDLYRQWTDRIMDEFFHQGDRERERGMEISPMCDKNTASVERTQVGFIDYIVHPLWETWADLVHPDAQDILDTLEDNRNW-YQSMIPQSPSPPFYT--------SDGEGSQHEDSEGRSVDSRFQFDLSMDSQDSQAGHGHHETTDLAVTCDHVNKEDLSDLEDVDTTTRDVSPAET*------------------------------------------------------------------------------------------------------------------------------------------------------------------------------------------------------
Olat_PDE4B2 TDMSKHMSLLADLKTMVETKKVTSSGVLLLDNYTDR---------IQVLRNMVHCADLSNPTKPLELYRQWTDRIMDEFFHQGDRERERGMEISPMCDKHTASVEKSQVGFIDYIAHPLWETWADLVHPDAQDILDTLEDNRNW-YQSMIPQSPSPPFYD---------QGTHGHSGGTGGQGGGDKFQFDVTLEEEDLDGIDRDGEGEDDEVDSLADSPPPEYVDNQASEDNEMIEPMTAIEIVTHEASPTDT*-----------------------------------------------------------------------------------------------------------------------------------------------------------------------------------------
Trub_PDE4B1 TDMSKHMSLLADLKTMVETKKVTSSGVLLLDNYTDR---------MQVLRNMVHCADLSNPTKPLDLYRQWTDRIMDEFFHQGDRERERGMEISPMCDKHTASVERTQVGFIDYIVHPLWETWADLVHPDAQDILDTLEDNRNW-YQSMIPQSPSPPFYA--------SDDEGRRPEEVEGGPAPSKFQFELTLDDQDGHG-----------------------------------------------------------------------------------------------------------------------------------------------------------------------------------------------------------------------------------------------
Trub_PDE4B2 TDMSKHMSLLADLKTMVETKKVTSSGVLLLDNYTDR---------IQVLRNMVHCADLSNPTKSLELYRQWTDRIMDEFFHQGDRERERGMEISPMCDKHTASVEKSQVGFIDYIVHPLWETWADLVHPDAQDILDTLEDNRNW-YQSMIPQSPSPPFYE---------QSTHGHGGGTGGQGGGEKFQFELTLEEEELDGIEKECLGDSHSPPLDCLDVQEQEGSMMEPMTAIEIVTHEASPTDT*-------------------------------------------------------------------------------------------------------------------------------------------------------------------------------------------------
Drer_PDE4B TDMSKHMSLLADLKTMVETKKVTSSGVLLLDNYTDR---------IQVLRNMVHCADLSNPTKSLELYRQWTDRIMEEFFHQGDRERERGMEISPMCDKHTASVEKSQVGFIDYIVHPLWETWADLVHPDAQDILDTLEDNRNW-YQSMIPQSPSPPFYQ-------------PDKEGNGGSSGPDKFQFELTLEEEDSEGTEKDEQSQGDEEEEAEEDRGEEMESTTHIQIVTEDASPVDT*-----------------------------------------------------------------------------------------------------------------------------------------------------------------------------------------------------
Hsap_PDE4C TDMSKHMNLLADLKTMVETKKVTSLGVLLLDNYSDR---------IQVLQNLVHCADLSNPTKPLPLYRQWTDRIMAEFFQQGDRERESGLDISPMCDKHTASVEKSQVGFIDYIAHPLWETWADLVHPDAQDLLDTLEDNREW-YQSKIPRSPS--------------------DLTNPERDGPDRFQFELTLEEAEEEDEEEEEEGEETALAKEALELPDTELLSPEAGPDPGDLPLDNQRT*---------------------------------------------------------------------------------------------------------------------------------------------------------------------------------------------------
Cjac_PDE4C TDMSKHMNLLADLKTMVETKKVTSLGVLLLDNYPDR---------IQVLQSLVHCADLSNPTKPLPLYRQWTDRIMAEFFQQGDRERESGLDISPMCDKHTASVEKSQVGFIDYIAHPLWETWADLVHPDAQDLLDTLEDNREW-YQSKIPRSPS--------------------SLTNPERGGPDKFQFGLPLEEAEEEEEEEEEEGEETALVKEALELSDTELPPKAVDSGDLPLDNQRT*-----------------------------------------------------------------------------------------------------------------------------------------------------------------------------------------------------
Mmus_PDE4C TDMSKHMSLLADLKTMVETKKVTSLGVLLLDNYSDR---------IQ----------------------------------QGDRERESGLDISPMCDKHTASMEKSQVGFIDYIAQPLWETWADLVHPDAQELLDTLEDNREW------YQSRI--------------------PCSPPHTMGSDRFKFELTLEEAEEEEEEEDEGQCTALNRESSELPST*-------------------------------------------------------------------------------------------------------------------------------------------------------------------------------------------------------------------------
Rnor_PDE4C TDMSKHMSLLADLKTMVETKKVTSLGVLLLDNYSDR---------IQVLQSLVHCADLSNPAKPLPLYRQWTERIMAEFFQQGDRERESGLDISPMCDKHTASVEKSQVGFIDYIAHPLWETWADLVHPDAQELLDTLEDNREW------YQSRV--------------------PCSPPHAIGPDRFKFELTLEETEEEEEEDERH*----------------------------------------------------------------------------------------------------------------------------------------------------------------------------------------------------------------------------------------
Cfam_PDE4C TDMSKHMNLLADLKTMVETKKVTSLGVLLLDNYSDR---------IQVLQNLVHCADLSNPTKPLELYRRWTDRIMAEFFQQGDRERESGLDISPMCDKHTASVEKSQVGFIDYIAHPLWETWADLVHPDAQDLLDTLEDNREW-YQSKIPRSPV--------------------DLTSPQQDSPDRFHFELTLEEAEEEEEEEGALDTEVSESPDTELLAPEASLDRGAPLLDNQRTGGKPCVDHEANVMAEPFGT*--------------------------------------------------------------------------------------------------------------------------------------------------------------------------------------
Ecab_PDE4C TDMSKHMSLLADLKTMVETKKVASLGVLLLDNYSDR---------IQVLQNLVHCADLSNPTKPLPLYRQWTDRIMAEFFQQGDRERESGLDVSPMCDKHTASVEKSQVGFIDYIAHPLWETWADLVHPDAQDLLDTLEDNREW-YQSKVPRSPM--------------------DPTSPEQGSPDRFQFELTLDEAEEEKEAEEEGALDGEASESPDTELLSPEASLDPGGLLLDNQRT--------------------------------------------------------------------------------------------------------------------------------------------------------------------------------------------------------
Btau_PDE4C TDMSKHMNLLADLKTMVETKKVTSLGVLLLDNYSDR---------IQVLQSLVHCADLSNPTKPLPLYRQWTDRIMAEFFQQGDSERESGLDISPMCDKHTASVEKSQVGFIDYIAHPLWETWADLVHPDAQDLLDTLEDNREW-YQSKIPRSPE--------------------DPTSPKQGSPDRFQFQLPLEEAEEEEEEEEEALDGEASKSPDTELLSSEASPDPGAPHLDYQRTVGKPCTDHEGNAVAEPLGT*-------------------------------------------------------------------------------------------------------------------------------------------------------------------------------------
Mdom_PDE4C TDMSKHMNLLADLKTMVETKKVTSLGVLLLDNYSDR---------IQVLQNMVHCADLSNPTKPLELYRQWTDRIMVEFFQQGDREREKGIEISPMCDKHTASVEKSQVGFIDYIAHPLWETWADLVHPDAQEILDTLEDNREW-YQSMIPRSPDGGHEA----------------GPAKGGGAADKFQFELTLEEEEGESDSEAEEPGSRAEEGEEENSGSDSKTLATDDSESAE------------------------------------------------------------------------------------------------------------------------------------------------------------------------------------------------------------
Xtro_PDE4C TDMSKHMNLLADLKTMVETKKVTSLGVLLLDNYSDR---------IQVLQNMVHCADLSNPTKPLEIYRQWTDRIIVEFFHQGDREREKGMEISPMCDKHNASVEKSQVGFIDYIVHPLWETWADLVHPDAQEILDTLEDNREW-YQSMIPQSPSPPPEE---------------QDPDRGNAAGDKFQFELTLEEEGESDTEQEDTESPIDEENSDSKIHATDDSAQASDEAEQSSPVDLCMTVSSKLDHLEIPPKSTLTKQKKILNFDNLKLAGERTIQQKEVSSSHATPANSARQEGTGQAEICLDQEGNITYLPLGT*----------------------------------------------------------------------------------------------------------------------
Olat_PDE4C1 TDMSKHINFLADMKTMVETKKVTNLGVLLLDNYSDRIQAKFGMLFLQVLQNMVHCADLSNPTKPLELYRKWTDRIMVELFTQGDREREKGMEISPMCDKQNASIEKTQVGFIDYVVHPLWETWADLVHPDAQEILDTLEDNREW-YQSMIPRSPSPTSPEEHHNEGRPSDGAVGTGGLAHLGGGGDKFQFELTLEEEEEEVDSDLESPIEEESQTSRDRHLDSSSP----------------------------------------------------------------------------------------------------------------------------------------------------------------------------------------------------------------------
Trub_PDE4C1 TDMSKHMNLLADLKTMVETKKVTSLGVLLLDNYSDR---------IQVLQNMVHCADLSNPTKPLELYRQWTDRIMVEFFTQGDRERDKGMEISPMCDKQNASIEKNQVGFIDYIVHPLWETWADLVHPDAQEILDTLEDNREW-YQSMIPRSPSPGPEQ-----QEVGGHAGEASALSRGSGSTDKFQFKLTLEEEEDKEAEQEDAPGVSRF-----------------------------------------------------------------------------------------------------------------------------------------------------------------------------------------------------------------------------------
Trub_PDE4C2 TDMSKHMNYLADMKTMVETKKVTSLGVLLLDNYSD---------RIQVLQNIVHCADLSNPTKPLELYRRWTDRIMREFFTQGDRERDKGMEISPMCDKHNASIEKSQVGFIDYIVHPLWETWADLVHPDAQEILDTLEDNREW-YQSMIPRSPSPSSPE--------------HDAKGSGGGGGDKFQFELTLEEEEEDEVESELESQKE-------------------------------------------------------------------------------------------------------------------------------------------------------------------------------------------------------------------------------------
Drer_PDE4C1 TDMSKHMNLLADLKTMVETKKVTSLGVLLLDNYSDR---------IQVLQNMVHCADLSNPTKPLELYRQWTDRIMVEFFTQGDRERDKGMEISPMCDKHNASIEKSQVGFIDYIVHPLWETWADLVHPDAQEILDTLEDNREW-YQSMIPHSPTSTPED-------KSAVMGMGAMGGGIASAGDKFQFELTLEEEGESDVESPVDEDLTSS-----------------------------------------------------------------------------------------------------------------------------------------------------------------------------------------------------------------------------------
Drer_PDE4C2 TDMSKHMNFLADLKTMVETKKVTSQGVLLLDNYSDR------IQVLQATFNMVHCADLSNPTKPLELYRKWTDRIMVEFFSQGDRERDKGIDVSPMCDKHTASMENTQVGFIDYIIHPLWETWADLVHPDAQDILDTLEDNREW-YQSMIPRSPSPTPEE------------QDSRPTGVTGSAGEKFQFELTLEEEDGELETEEEHTDIERSRTMTDPEHPQTSPLGITTILDSSERELDQELTSVSALQLETSSSSTHE*----------------------------------------------------------------------------------------------------------------------------------------------------------------------------------
Gacu_PDE4C TDMSKHMNLLADLKTMVETKKVTSLGVLLLDNYSDR---------IQVLQNMVHCADLSNPTKPLELYRRWTDRIMVEFFTQGDRERDKGMEISPMCDKQNASIEKNQVGFIDYIVHPLWETWADLVHPDAQEILDTLEDNREW-YQSMIPHSPSPHPED---QQEGARAGESSALIGGGGSVSADKFQFELTLEEERESDTESPPEEEEGFSSSRGTELSRTDSGSRTFSLDSDMAEDREADQE---------------------------------------------------------------------------------------------------------------------------------------------------------------------------------------------------
Hsap_PDE4D TDMSKHMNLLADLKTMVETKKVTSSGVLLLDNYSDR---------IQVLQNMVHCADLSNPTKPLQLYRQWTDRIMEEFFRQGDRERERGMEISPMCDKHNASVEKSQVGFIDYIVHPLWETWADLVHPDAQDILDTLEDNREW-YQSTIPQSPSPAPDD----------------PEEGRQGQTEKFQFELTLEEDGESDTEKDSGSQVEED----------TSCSDSKTLCTQDSESTEIPLDEQVEEEAVGEEEESQPEACVIDDRSPDT*----------------------------------------------------------------------------------------------------------------------------------------------------------------------
Gacu_PDE4D TDMSKHMNLLADLKTMVETKKVTSSGVLLLDNYSDR---------IQVLQNMVHCADLSNPTKPLQLYRQWTDRIMDEFFSQGDRERERGMEISPMCDKHNASVEKSQVGFIDYIVHPLWETWADLVHPDAQDILDTLEDNREW-YQSTIPQSPSPALDD---------------PEDGSRPPGGDKFQFELTLEEDGESDTEKDSGSQPEE----------EEDCTDSKTLCTQDSESTEIPLDEQVGEDEGEEEEEVTE-----------------------------------------------------------------------------------------------------------------------------------------------------------------------------------
Olat_PDE4D TDMSKHMNLLADLKTMVETKKVTSSGVLLLDNYSDR---------IQVLQNMVHCADLSNPTKPLQLYRQWTDRIMEEFFSQGDRERERGMEISPMCDKHNASVEKSQVGFIDYIAHPLWETWADLVHPDAQDILDTLEDNREW-YQSTIPQSPSPALDE---------------PEDGSRPPGGDKFQFELTLEEDGESDTEKDSGSQPEE----------ENSCTDSKTLCTQDSESTEIPLDEQVGEDEDDEEEEHE------------------------------------------------------------------------------------------------------------------------------------------------------------------------------------
Trub_PDE4D TDMSKHMNLLADLKTMVETQKVTSSGVLLLDNYSDR---------IQVLQNMVHCADLSNPTKPLQLYRQWTDRIMEEFFSQGDRERERGMEISPMCDKHNASVEKNQVGFIDYIVHPLWETWADLVHPDAQVILDTLEDNREF-YQSTIPQSPSPTLDE---------------PEDGTRPPGGDKFQFELTLEEDGESDTEKDSGSQPEEDEEEEEEDEEENSCTDSKTLCTQDSESTEIPLDEQVGLATPPVSFVQT------------------------------------------------------------------------------------------------------------------------------------------------------------------------------------
Acar_PDE4D TDMSKHMNLLADLKTMVETKKVTSSGVLLLDNYSDR---------IQVLQNMVHCADLSNPTKPLQLYRQWTDRIMEEFFRQGDRERERGMEISPMCDKHNASVEKSQVGFIDYIVHPLWETWADLVHPDAQDILDTLEDNREW-YQSTIPQSPSPAPDD----------------QEEGRQGQTDKFQFELTLEEDGESDTEKDSGSQVEED----------TSCSDSKTLCTQDSESTEIPLDEQVGGEVEEEEEEEQSQTEHCVQEEHSPDT*--------------------------------------------------------------------------------------------------------------------------------------------------------------------
Cfam_PDE4D TDMSKHMNLLADLKTMVETKKVTSSGVLLLDNYSDR---------IQVLQNMVHCADLSNPTKPLQLYRQWTDRIMEEFFRQGDRERERGMEISPMCDKHNASVEKSQVGFIDYIVHPLWETWADLVHPDAQDILDTLEDNREW-YQSTIPQSPSPAPDE----------------QEEGRQGQTEKFQFELTLEEDGESDTEKDSGSQVEED----------TSCSDSKTLCTQDSESTEIPLDEQVEEETVGEEENSQPEACVIEDHSPDT*----------------------------------------------------------------------------------------------------------------------------------------------------------------------
Btau_PDE4D TDMSKHMNLLADLKTMVETKKVTSSGVLLLDNYSDR---------IQVLQNMVHCADLSNPTKPLQLYRQWTDRIMEEFFRQGDRERERGMEISPMCDKHNASVEKSQVGFIDYIVHPLWETWADLVHPDAQDILDTLEDNREW-YQSTIPQSPSPAPDD----------------QEEGRQGQTEKFQFELTLEEDGESDTEKDSGSQVEED----------TSCSDSKTLCTQDSESTEIPLDEQVEEEAVADEE-SQPEACVIGDPSPDT*----------------------------------------------------------------------------------------------------------------------------------------------------------------------
Mdom_PDE4D TDMSKHMNLLADLKTMVETKKVTSSGVLLLDNYSDR---------IQVLQNMVHCADLSNPTKPLQLYRQWTDRIMEEFFRQGDRERERGMEISPMCDKHNASVEKSQVGFIDYIVHPLWETWADLVHPDAQDILDTLEDNREW-YQSTIPQSPSPAPDD----------------QEEGRQGQTEKFQFELTLEEDGESDTEKDSGSPVEED----------TSCSDSKTLCTQDSESTEIPLDEQVEEEAVGEEEEEEDPAEPCAIENEHSPDT*-------------------------------------------------------------------------------------------------------------------------------------------------------------------
Ecab_PDE4D TDMSKHMNLLADLKTMVETKKVTSSGVLLLDNYSDR---------IQVLQNMVHCADLSNPTKPLQLYRQWTDRIMEEFFRQGDRERERGMEISPMCDKHNASVEKSQVGFIDYIVHPLWETWADLVHPDAQDILDTLEDNREW-YQSTIPQSPSPAPDD----------------QEEGRQGQTEKFQFELTLEEDGESDTEKDSGSQVEED----------TSCSDSKTLCTQDSESTEIPLDEQVEEEAVGEEEESQPEACAAEDRSPDT*----------------------------------------------------------------------------------------------------------------------------------------------------------------------
Rnor_PDE4D TDMSKHMNLLADLKTMVETKKVTSSGVLLLDNYSDR---------IQVLQNMVHCADLSNPTKPLQLYRQWTDRIMEEFFRQGDRERERGMEISPMCDKHNASVEKSQVGFIDYIVHPLWETWADLVHPDAQDILDTLEDNREW-YQSTIPQSPSPAPDD----------------QEDGRQGQTEKFQFELTLEEDGESDTEKDSGSQVEED----------TSCSDSKTLCTQDSESTEIPLDEQVEEEAVAEEE-SQPQTGVADDCCPDT*----------------------------------------------------------------------------------------------------------------------------------------------------------------------
Mmus_PDE4D TDMSKHMNLLADLKTMVETKKVTSSGVLLLDNYSDR---------IQVLQNMVHCADLSNPTKPLQLYRQWTDRIMEEFFRQGDRERERGMEISPMCDKHNASVEKSQVGFIDYIVHPLWETWADLVHPDAQDILDTLEDNREW-YQSTIPQSPSPAPDD----------------QEEGRQGQTEKFQFELTLEEDCESDTEKDSGSQVEED----------TSCSDSKTLCTQDSESTEIPLDEQVEEEAVAEEE-SQPETCVPDDCCPDT*----------------------------------------------------------------------------------------------------------------------------------------------------------------------
Cjac_PDE4D TDMSKHMNLLADLKTMVETKKVTSSGVLLLDNYSDR---------IQVLQNMVHCADLSNPTKPLQLYRQWTDRIMEEFFRQGDRERERGMEISPMCDKHNASVEKSQVGFIDYIVHPLWETWADLVHPDAQDILDTLEDNREW-YQSTIPQSPSPAPDD----------------PEEGRQGQTEKFQFELTLEEDGESDTEKDSGSQVEED----------TSCSDSKTLCTQDSESTEIPLDEQVEEEAVGEEEESQPEACVIDDRSPDT*----------------------------------------------------------------------------------------------------------------------------------------------------------------------
Drer_PDE4D TDMSKHMNLLADLKTMVETKKVTSSGVLLLDNYSDR---------IQVLQNMVHCADLSNPTKPLQLYRQWTDRIMEEFFSQGDRERERGMEISPMCDKHNASVEKSQVGFIDYIVHPLWETWADLVHPDAQDILDTLEDNREW-YQSTIPQSPSPALDT---------------SSEGRRAAGQEKFQFELTLEEDGESDTEKDSGSPPEEDEEEEEE--EENSCSDSKTLCTQDSECTEIPLDEQVGDVPHEDEYEHEENREISEPCVLEEEEEEEEEEEEDTANT*-------------------------------------------------------------------------------------------------------------------------------------------------------
*
‡
‡
160
180
200
220
240
260
280
300
320
340
360
380
400
420
440
460
1
20
40
60
80
100
120
140
PDEase_I Subdomain
Carboxy-Termini
Catalytic Domain
“KIM” Motif
‡
*
*
600
640
800
900
480
540
580
620
660
680
780
820
840
860
880
500
520
560
700
720
740
760
