# Supplementary figures and images for: An evolutionary analysis of cAMP-specific Phosphodiesterase 4 alternative splicing
Source: BMC Evol Biol. 2010 Aug 11;10:247. doi: 10.1186/1471-2148-10-247 (PMC2929239; doi:10.1186/1471-2148-10-247)

## Slide 1
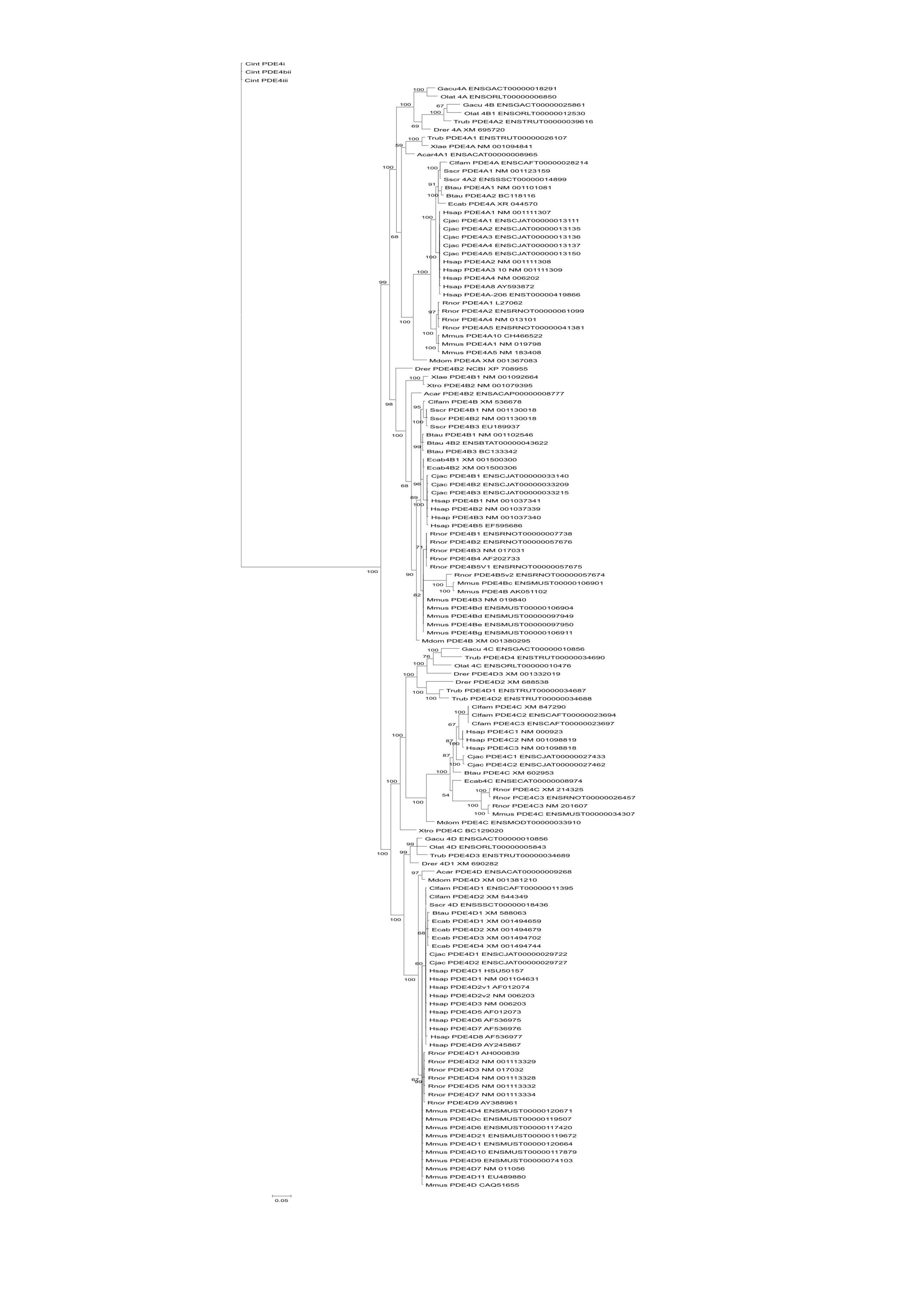

Supplement: Additional file 3 — Supplemental Figure S2. Phylogenetic classification of PDE4 Splice Variants Used in This Study. Supplemental Figure S2 is a Bayesian phylogenetic tree constructed using PDE4 catalytic domain amino acid sequences from each PDE4 gene isoform, from each taxa used in this study. [file 1471-2148-10-247-S3.PPT]

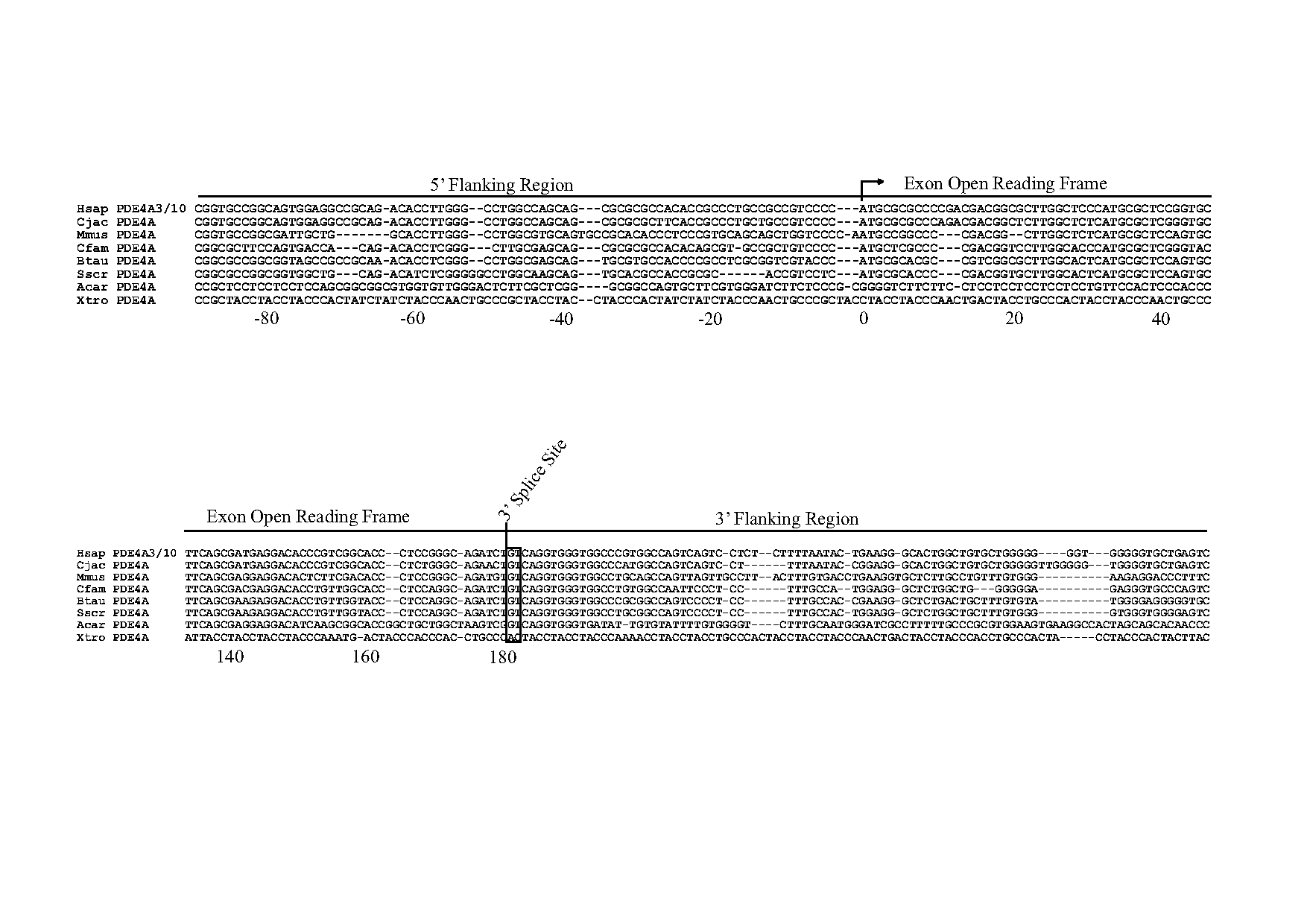

Supplement: Additional file 5 — Supplemental Figure S3. PDE4 Exon Formation Through Sequence Drift. Supplemental Figure S3 is a multiple sequence alignment of flanking and coding nucleotide sequences orthologous to the H. sapiens splice variant PDE4A3/10, showing the formation of this exon occurred through sequence drift. [file 1471-2148-10-247-S5.PNG]

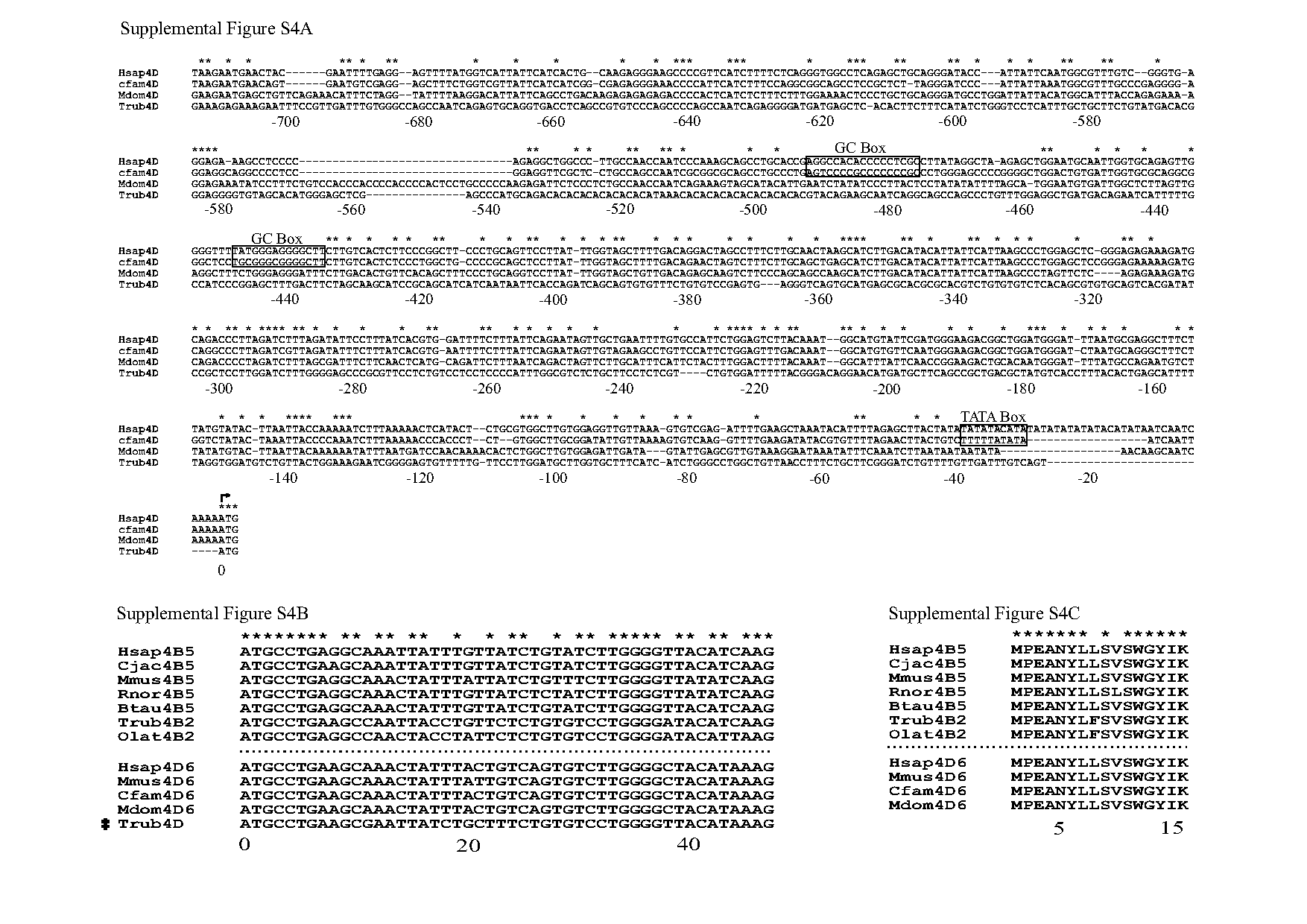

Supplement: Additional file 6 — Supplemental Figure S4. PDE4 Splice Variant Promoter Formation Through Sequence Drift. Supplemental Figure S4 is a multiple sequence alignments of the PDE4D6 upstream flanking region, PDE4B5 and PDE4D6 5'exons, and corresponding amino acid sequences. [file 1471-2148-10-247-S6.PNG]
