# Supplementary material for: An evolutionary analysis of cAMP-specific Phosphodiesterase 4 alternative splicing
Source: BMC Evol Biol. 2010 Aug 11;10:247. doi: 10.1186/1471-2148-10-247 (PMC2929239; doi:10.1186/1471-2148-10-247)
Supplement: Additional file 4 — Supplemental Table S2. Supplemental Table S2 lists orthologous PDE4 splice variants with different designations in nomenclature. Three PDE4A and three PDE4 D splice variants have homologous amino termini but have different names between humans and rodents. [file 1471-2148-10-247-S4.DOC]

SI Table 2. Orthologous PDE4 splice variants with different designations in nomenclature. Three PDE4A and three PDE4D splice variants have homologous amino termini but have different names between humans (Hsap), mouse (Mmus), and rat (Rnor).

| PDE4A Long Form | PDE4A Long Form | PDE4A Super-Short Form | PDE4D Long Form | PDE4D Long Form | PDE4D Long Form |
| --- | --- | --- | --- | --- | --- |
| Hsap4A1 | Hsap4A3/10 | Hsap4A4 | Hsap4D8 | Hsap4D1 | Hsap4D7 |
| Mmus4A5 | Mmus4A10 | Mmus4A1 | Mmus | Mmus4D4 | Mmus4D7 |
| Rnor4A4 | Rnor4A10 | Rnor4A1 | Rnor4D7 | Rnor4D4 | Rnor4D2 |
